# Private sector quality of care for maternal, new-born, and child health in low-and-middle-income countries: a secondary review

**DOI:** 10.3389/fgwh.2024.1369792

**Published:** 2024-04-19

**Authors:** Georgina Morris, Blerta Maliqi, Samantha R. Lattof, Joe Strong, Nuhu Yaqub

**Affiliations:** ^1^ Department of International Development, London School of Economics and Political Science, London, United Kingdom; ^2^ Department of Maternal, Newborn, Child, Adolescent Health and Ageing, World Health Organization, Geneva, Switzerland; ^3^ Regional Office for Africa, World Health Organization, Brazzaville, Congo

**Keywords:** systematic review, private health sector, maternal health, newborn health, child health, quality of care, quality, low-and middle-income countries

## Abstract

The private sector has emerged as a crucial source of maternal, newborn, and child health (MNCH) care in many low- and middle-income countries (LMICs). Quality within the MNCH private sector varies and has not been established systematically. This study systematically reviews findings on private-sector delivery of quality MNCH care in LMICs through the six domains of quality care (QoC) (i.e., efficiency, equity, effectiveness, people-centered care, safety, and timeliness). We registered the systematic review with PROSPERO international prospective register of systematic reviews (registration number CRD42019143383) and followed the Preferred Reporting Items for Systematic reviews and Meta-Analyses (PRISMA) Statement for clear and transparent reporting of systematic reviews and meta-analyses. Searches were conducted in eight electronic databases and two websites. For inclusion, studies in LMICs must have examined at least one of the following outcomes using qualitative, quantitative, and/or mixed-methods: maternal morbidity, maternal mortality, newborn morbidity, newborn mortality, child morbidity, child mortality, service utilization, quality of care, and/or experience of care including respectful care. Outcome data was extracted for descriptive statistics and thematic analysis. Of the 139 included studies, 110 studies reported data on QoC. Most studies reporting on QoC occurred in India (19.3%), Uganda (12.3%), and Bangladesh (8.8%). Effectiveness was the most widely measured quality domain with 55 data points, followed by people-centered care (*n* = 52), safety (*n* = 47), timeliness (*n* = 31), equity (*n* = 24), and efficiency (*n* = 4). The review showed inconsistencies in care quality across private and public facilities, with quality varying across the six domains. Factors such as training, guidelines, and technical competence influenced the quality. There were also variations in how domains like “people-centered care” have been understood and measured over time. The review underscores the need for clearer definitions of “quality” and practical QoC measures, central to the success of Sustainable Development Goals (SDGs) and equitable health outcomes. This research addresses how quality MNCH care has been defined and operationalized to understand how quality is delivered across the private health sector and the larger health system. Numerous variables and metrics under each QoC domain highlight the difficulty in systematizing QoC. These findings have practical significance to both researchers and policymakers.

**Systematic Review Registration:**

https://bmjopen.bmj.com/content/10/2/e033141.long, Identifier [CRD42019143383].

## 1 Introduction

The private health sector—“individuals and organizations that are neither owned nor directly controlled by governments and are involved in the provision of health services” (1) (p. 1)—has emerged as a crucial source of maternal, newborn, and child health (MNCH) care in many low- and middle-income countries (LMICs) (2, 3). Moreover, one in five births in LMICs occurs in the private sector (2). Quality within the private sector varies (4, 5) and has not yet been established systematically for MNCH.

Quality of care (QoC) is of upmost importance to achieving the Sustainable Development Goals (SDGs) and universal health coverage (UHC). Improvements in access to care without appropriate attention to the quality of that care will not produce the desired improvements in MNCH outcomes (6). The need to align the performance of public and private healthcare delivery sectors is particularly important given fragmentation in mixed-health markets (6). The myriad of ways in which researchers and practitioners define quality and select metrics often leads to a reductionist approach. This approach overlooks a more comprehensive picture of “quality” and its multiple facets. It is pertinent that those working towards improving equitable MNCH outcomes gain clarity on what “quality care” means, and how to operationalize QoC.

Despite the importance of QoC in global health discourse, the private sector's delivery of QoC for mothers, newborns, and children in LMICs has not been thoroughly synthesized or reviewed. Dettrick et al. and Berendes et al. reviewed strategies for MCH care, but did not focus on private sector or MNCH quality care (7, 8). Benova et al. compared the equity and quality of childbirth care in private and public sectors across 57 LMICs, but did not extend to antenatal care (ANC) or postnatal care (2). Madhavan and Bishai reviewed evidence on private sector engagement in sexual and reproductive health and maternal and neonatal health services, focusing on equity, quality, and cost-effectiveness (9). Brugha and Zwi provided a framework for improving quality of private sector service delivery in 1998, though this does not relate specifically to MNCH care (10). Finally, Chou et al. estimated the global impact of poor QoC on maternal and neonatal outcomes in 81 LMICs (11). While this study reaffirmed the importance of ensuring quality care for MNCH, it did not contribute towards reviewing available evidence. Assessing quality of private healthcare provision is integral to determining progress towards equitable MNCH outcomes globally (12).

This study systematically reviews findings on private-sector delivery of quality MNCH care in LMICs through the lens of the six domains of quality care (i.e., efficiency, equity, effectiveness, people-centered care, safety, and timeliness), a framework put forward by the Institute of Medicine (13). The impetus behind this study comes from the Network for Improving Quality of Care for Maternal, Newborn and Child Health (the Network). The Network, a partnership of 10 countries (Bangladesh, Côte d’Ivoire, Ethiopia, Ghana, India, Malawi, Nigeria, Sierra Leone, the United Republic of Tanzania, and Uganda) and their technical partners, aims to reduce preventable maternal and newborn deaths and stillbirths (14). Network members recognize the importance of both the public and private health sectors in achieving MNCH goals and delivering high-quality care.

We conducted this systematic review as part of a larger study on private-sector delivery of quality care for MNCH in LMICs (15, 16). This paper aims to answer the primary research question: How effective and efficient is the private sector at delivering QoC? The objectives of this paper are threefold:

1. To explore how effective and efficient the private sector is at delivering quality MNCH care through the six domains of QoC;
2. To unpack and map how researchers have interpreted and operationalized QoC in their studies over time; and
3. To present interventions that have been effective or ineffective at improving quality of private sector MNCH care in various settings and across different facility types [e.g., private, public, non-governmental organization (NGO), faith-based].

## 2 Methods

Guidance from the Preferred Reporting Items for Systematic reviews and Meta-Analyses (PRISMA) Statement for clear and transparent reporting of systematic reviews and meta-analyses informed this research (17, 18). For inclusion in this systematic review, qualitative, quantitative, and/or mixed-methods studies in LMICs must have examined at least one of the following outcomes: maternal morbidity, maternal mortality, newborn morbidity, newborn mortality, child morbidity, child mortality, service utilization, components of QoC (i.e., safety, effectiveness, timeliness, efficiency, equity, people-centered care), and/or experience of care including respectful care (Table 1). Studies must have been published in English, French, German, or Italian. Ethical approval was not required. We registered the search with the PROSPERO international prospective register of systematic reviews (registration number CRD42019143383).

**Table 1:** PICOTS criteria used in the systematic review.

PICOTS	
Populations	Pregnant people, mothers, newborns, and children (aged nine years and under)
Interventions	Delivery of quality maternal, newborn, and/or child health services by the private sector
Control	Not necessary
Outcomes	Quantitative, qualitative, or mixed-methods data on: • Maternal morbidity • Maternal mortality • Newborn morbidity • Newborn mortality • Child morbidity • Child mortality • Components of quality care (i.e., safety, effectiveness, timeliness, efficiency, equity, people-centered care) • Experience of care, including respectful care • Service utilization
Timeframe	1 January 1995 to 30 June 2019
Setting	Low- and middle-income countries

Following our published protocol and data extraction tools (15), SRL conducted searches in eight electronic databases (Cumulative Index to Nursing and Allied Health, EconLit, Excerpta Medica Database, International Bibliography of the Social Sciences, Popline, PubMed, ScienceDirect, and Web of Science) and two websites (Health Care Provider Performance Review and the Maternal healthcare markets Evaluation Team at the London School of Hygiene & Tropical Medicine). She supplemented these searches with hand searching of reference lists and expert-recommended articles. Search terms appear in Table 2, and the full electronic search strategy for each database appears in the protocol (15). SRL completed the searches on 23 June 2020.

**Table 2:** Search terms and their combinations.

1. Private sector	2. Quality of care	3. MNCH
Private sector	Quality	Matern*
For-profit		Pregnan*
For profit		Mother*
Public-private		Newborn*
Private enterprise*		Infant*
NGO		Child*
Non-government*		Pediatric*
		Paediatric*
		Neonat*

*is used at the end of a word when used for search terms broadens a search by finding words that start with the same letters. This helps to find distinctive word stems to retrieve variations of a term.

Quantitative and qualitative data were extracted on the following categories:

• Background information (e.g., author, date, setting, study objective)
• Intervention background information (e.g., implementing agency, geographic level, study population)
• Intervention details (e.g., intervention recipients, nature of intervention, dimensions of quality care)
• Critical outcomes (both quantitative and qualitative):
  ◦ Maternal morbidity
  ◦ Maternal mortality
  ◦ Newborn morbidity
  ◦ Newborn mortality
  ◦ Child morbidity
  ◦ Child mortality
  ◦ Service utilization
  ◦ Experience of care, including respectful care
  ◦ Components of quality care (i.e., safety, effectiveness, timeliness, efficiency, equity, people-centered care)
• Evaluation/study details (e.g., study type, data type, intervention claims, strategy effectiveness, cost data)
• Mechanisms for engaging the private sector in delivering quality MNCH care
• Study quality (qualitative and quantitative)

JS and SRL extracted and quality assessed studies in duplicate using the Effective Public Health Practice Project quality assessment tool for quantitative studies (19) and Miltenburg et al.'s quality assessment tool (based on criteria developed by Walsh and Downe) for qualitative studies (20, 21). The analysis synthesizes data from studies that reported QoC outcomes.

Using a three-stage approach (21), data were coded with descriptive codes that addressed metrics for each QoC outcome. These codes were collated into broader descriptive variables for each of the six quality domains. Table 6 displays the various ways that studies in this systematic review interpreted and operationalized QoC. The multitude of variables under each domain and metrics for each variable reflects the broader concerns in literature that QoC is hard to systematize. Several studies encompassed more than one QoC domain and more than one variable within each domain, meaning that the total number of data points referenced in the tables of results exceed the 110 studies included in this paper. Notably, one of the 19 studies included under “wait times and service readiness” was coded as “timeliness” (22). Yet, this study referred to “travel time to the clinic” which could also be perceive as “geographic access” under the equity domain. Crossovers such as this were unavoidable and reflect the intricate and multifaceted interpretations of QoC.

Variability in study designs, interventions, and outcomes prevented a meta-analysis. We present the descriptive statistics of our findings and illustrate this heterogeneity in QoC outcomes. Quantitative and qualitative findings then appear in a narrative synthesis organized by the six domains of QoC. Detailed summary tables for the included studies, including quality scores, appear in Supplementary Material S1.

## 3 Results

### 3.1 Descriptive statistics

We conducted this systematic review as part of a larger study on private-sector delivery of quality care for MNHC in LMICs (15, 16). The searches and screening were conducted together. The search generated 5,345 items for screening (Supplementary Figure S1). After removing duplicates, the 3,788 remaining items were screened for inclusion based on title and abstract. Where exclusion could not be determined based on title and abstract, we screened the full text. Of 778 full texts screened, 139 studies met all the inclusion criteria and were included in the full systematic review. This paper focuses on the studies which reported outcome data on QoC (*n* = 110) (Table 3). Some studies presented multiple relevant outcomes, so the total number of data points in Table 3 exceeds the number of studies in the final inventory. The remaining findings in this article focus on the 110 studies that reported data on QoC.

**Table 3:** Outcomes of included studies.

Reported study outcomes	Number of studies in final inventory that report the outcome	Number of studies reporting on the study outcome and on quality of care
Maternal morbidity	15	9
Maternal mortality	6	1
Infant morbidity	6	4
Infant mortality	16	12
Child morbidity	14	10
Child mortality	10	7
Quality of care	110	–
Experience of care	45	34
Service utilization	7	5
Infant/child growth^a^	9	6

^a^ Secondary outcome.

Most studies reporting on QoC occurred in India (19.3%), Uganda (12.3%), and Bangladesh (8.8%) (Table 4). Most studies presented quantitative data (74.5%), with 6.4% of studies presenting exclusively qualitative data and 19.1% of studies presenting both quantitative and qualitative data (Table 5). Over half of studies (53.6%) occurred in countries classified as lower-middle-income. The level of geographic coverage varied with over half of studies conducted at the sub-national level (53.6%) and one-fourth of studies conducted at the national level (24.5%).

**Table 4:** Included studies by region and country.

Region/country	Number of studies included in final inventory (%)	Number of studies reporting on quality of care (%)	Region/country	Number of studies included in final inventory (%)	Number of studies reporting on quality of care (%)
**Africa**	**49** **(35.3%)**	**36** **(32.7%)**	**Asia**	**67** **(48.2%)**	**56** **(50.9%)**
Angola	1 (0.7%)	–	Afghanistan	2 (1.4%)	2 (1.8%)
Côte D'Ivoire	1 (0.7%)	1 (0.9%)	Bangladesh	11 (7.9%)	10 (8.8%)
Ethiopia	2 (1.4%)	–	China	2 (1.4%)	2 (1.8%)
Ghana	1 (0.7%)	–	Georgia	1 (0.7%)	1 (0.9%)
Kenya	11 (7.9%)	9 (7.9%)	India	30 (21.6%)	22 (19.3%)
Lesotho	1 (0.7%)	1 (0.9%)	Indonesia	2 (1.4%)	1 (0.9%)
Malawi	3 (2.2%)	3 (2.6%)	Iran	2 (1.4%)	2 (1.8%)
Niger	1 (0.7%)	1 (0.9%)	Jordan	1 (0.7%)	–
Nigeria	3 (2.2%)	1 (0.9%)	Nepal	4 (2.9%)	4 (3.5%)
Tanzania	3 (2.2%)	2 (1.8%)	Pakistan	6 (4.3%)	4 (3.4%)
The Gambia	1 (0.7%)	–	Philippines	2 (1.4%)	1 (0.9%)
Uganda	15 (10.8%)	14 (12.3%)	Sri Lanka	2 (1.4%)	2 (1.7%)
Zambia	2 (1.4%)	2 (1.8%)	Turkey	2 (1.4%)	2 (1.8%)
Multiple countries	4 (2.9%)	4 (3.5%)			
			**Oceania**	**1** **(0.7%)**	**1** **(0.9%)**
**Latin America & Caribbean**	**14** **(10.1%)**	**11** **(10.0%)**	Papua New Guinea	1 (0.7%)	1 (0.9%)
Brazil	5 (3.6%)	2 (1.8%)			
Guatemala	2 (1.4%)	2 (1.8%)	**Cross-Regional Studies**	**8** **(5.78%)**	**6** **(5.5%)**
Haiti	2 (1.4%)	2 (1.8%)			
Mexico	4 (2.9%)	4 (3.5%)			
Multiple countries	1 (0.7%)	1 (0.9%)	**Total**	**139** **(100%)**	**110** **(100%)**

The bold text refers to the sum of the individual countries within the region, presented in bold by region. The percentages refer to the number of studies included in final inventory as a percentage of the total.

**Table 5:** Characteristics of included studies.

Characteristics	Number of studies included in final inventory (%)	Number of studies reporting on quality of care (%)
Methodology		
Randomized controlled trial	1 (0.7%)	1 (0.9%)
Controlled clinical trial	1 (0.7%)	1 (0.9%)
Cohort analytic	10 (7.2%)	10 (9.1%)
Case-control	2 (1.4%)	1 (0.9%)
Controlled (before & after)	7 (5.0%)	7 (6.4%)
Interrupted time series	1 (0.7%)	–
Qualitative	8 (5.8%)	7 (6.4%)
Mixed methods	21 (15.1%)	16 (14.5%)
Regression	55 (39.6%)	41 (37.3%)
Other	31 (22.3%)	24 (21.8%)
Unclear/not specified	2 (1.4%)	2 (1.8%)
Country income group		
Low	33 (23.7%)	28 (25.5%)
Lower-middle	75 (54.0%)	59 (53.6%)
Upper-middle	19 (13.7%)	15 (13.6%)
Multiple	12 (8.6%)	8 (7.3%)
Geographical level		
National	34 (24.5%)	27 (24.5%)
Sub-national (e.g., state, city)	73 (52.5%)	59 (53.6%)
Local (e.g., village)	7 (5.0%)	5 (4.5%)
Health facility	18 (12.9%)	14 (12.7%)
Other	5 (3.6%)	4 (3.6%)
Unclear/not specified	2 (1.4%)	1 (0.9%)
Study population		
Pregnant women	11 (7.9%)	10 (9.1%)
Women during childbirth	2 (1.4%)	1 (0.9%)
Mothers postpartum	12 (8.6%)	11 (10.0%)
Infants	13 (9.4%)	12 (10.9%)
Children	9 (6.5%)	4 (3.6%)
Health care providers	41 (29.5%)	35 (31.8%)
Parents/child caretakers	4 (2.9%)	3 (2.7%)
Multiple answers from list	26 (18.7%)	17 (15.5%)
Other (e.g., urban poor, married women)	20 (14.4%)	16 (14.5%)
Unclear/unspecified	1 (0.7%)	1 (0.9%)
Publication type		
Peer-reviewed journal article	103 (74.1%)	77 (70.0%)
Report	27 (19.4%)	24 (21.8%)
Book or book chapter	1 (0.7%)	1 (0.9%)
Other (e.g., conference paper, abstract)	8 (5.8%)	8 (7.3%)
Implemented a specific intervention beyond the delivery of quality care?		
Yes	58 (41.7%)	47 (42.7%)
No	81 (58.3%)	63 (57.3%)
Type of data		
Quantitative	104 (74.8%)	82 (74.5%)
Qualitative	8 (5.8%)	7 (6.4%)
Both	27 (19.4%)	21 (19.1%)
Longitudinal data?		
Yes	45 (32.4%)	36 (32.7%)
No	90 (64.7%)	72 (65.5%)
Unclear/not specified	4 (2.9%)	2 (1.8%)

About two in five studies (42.7%) assessed a specific intervention or interventions beyond the generic delivery of quality MNCH care services. These interventions most often addressed QoC from the supply side (59.6%) or from both the supply and demand sides (36.2%). Interventions most frequently addressed program management (57.5%), followed by on-site support for quality improvement (53.2%), advocacy (53.2%), learning systems (46.8%), data systems (21.3%), and strategy development (19.2%). Over half intervention studies (55.3%) reported that the intervention had a positive impact. Only 10.6% of studies reported that the interventions had a negative impact, and 31.9% of studies reported that the interventions had a mixed impact.

Three in five studies used the term “quality” without explicitly defining it. Less than half of studies (42.7%) referenced one or more of the six domains within quality. Effectiveness was the most widely measured QoC domain with 55 data points, followed by people-centered care (*n* = 52), safety (*n* = 47), timeliness (*n* = 31), equity (*n* = 24), and efficiency (*n* = 4).

While equity is the domain with the greatest number of articles published in earlier years (2000–2010), the number of studies that measured equity were lower than the number of studies that addressed the other five domains. Equity also has the highest proportion of unspecified variables measured (27%) compared to the other domains (8%–15%). The only exception to this observation is the efficiency domain which had only four articles, two of which did not provide sufficient information to assist in categorizing the variables. Most studies measuring equity were published in 2000–2010. Articles measuring efficiency were most often published from 2010 to 2015. Effectiveness, people-centered care, safety, and timeliness were most often measured in articles published from 2015 to 2020.

### 3.2 Effectiveness

Forty-seven studies reported 55 data points on effective QoC through variables such as quality; coverage; ability to deliver; training, guidelines, and knowledge; technical competence; and hygiene (Table 6). The two primary themes that emerged from these studies address: (1) components and comprehensiveness of care received; and (2) effective training, guidance, and technical quality of caregiving.

**Table 6:** Quality of care measures and metrics organized by the effectiveness domain.

Domain (*n*)	Variable measured	Metric(s) used	% of studies under the domain that measured the variable (*n*)^a^	Most common year of publication in five-year windows (*n*)
Effectiveness (*n* = 47)	Quality	Evidence-based health interventions, completion of recommended clinical practices, overall quality aggregate, quality of information and services in reproductive child health (RCH) services	15% (8)	2015–2020 (3)
	Coverage	Disease management, coverage of recommended antenatal care (ANC) components, full immunization coverage, attended deliveries, components of ANC received	8% (4)	2015–2020 (3)
	Ability to deliver	Shortage of human/physical resources including vaccines, effective responses to emergencies, availability of emergency crash cart (a wheeled cart stocked with equipment, supplies, and drugs for us in resuscitations), “structural quality”	15% (8)	2015–2020 (6)
	Training, guidelines, and knowledge	Number of guidelines and job aids available, supervision	12% (6)	2015–2020 (3)
	Technical competence	Following guidelines, avoiding underuse, family planning technical score, essential newborn care indicators met, knowledge parameters, performance, performance on Balance Scorecard (BSC) domains, qualified providers, technical competence on specific diseases under study: - Treatment of diarrhea with oral rehydration therapy (ORT) and zinc - Acute respiratory infections (ARI) disease management practices - Association between the occurrence of an emergence caesarean section and complete monitoring during labor - Uterine evac with recommended tech - Appropriate knowledge and treatment of diarrhea and pneumonia - A diagnoses and treatment - Quality pregnancy care in terms of blood pressure measured and advice on pregnancy - Vignettes administered concurrently to all public	46% (24)	2015–2020 (12)
	Hygiene	Cases of preventable infection	2% (1)	2015–2020 (1)
	Unspecified		8% (4)	2015–2020 (2)

^a^ The number of data points in column 4 (*n* = 55) exceeds the number of studies that reported on this domain, as some studies examined multiple variables for effectiveness.

#### 3.2.1 Components and comprehensiveness of care received

Comprehensive care received frequently focused on effectively managing and treating conditions such as diarrhea. An evaluation of the World Health Partners Sky program in India, which aimed to increase the basic service delivery of MNCH care, found no effect of the intervention on improving effective treatments for childhood diarrhea or pneumonia (23). At follow-up, a further study of the intervention found that providers were seven percentage points likelier to prescribe the correct treatment in response to pneumonia and seven percentage points less likely to prescribe harmful treatments (24).

Among providers of acute respiratory infection (ARI) management in India, many did not check the respiratory rate and chest in drawing while providing care (25). An intervention that aimed to improve the quality of child healthcare among private providers in India utilized WHO case management guidelines and training to improve the number of providers giving adequate care by 22% (26). Training Community Health Promoters in Uganda to provide services and products under retail price increased the likelihood of caregivers purchasing effective treatments for ARI and diarrhea than those in non-intervention villages, and trained Community Health Promoters were associated with an increase in effective direct treatment of child sickness (27).

Additional studies on comprehensive care components evaluated whether women received full ANC and maternity care. A comparative study of women attending maternity care in China found that fewer women in private facilities had hemoglobin, urine, syphilis, HBV, HIV/AIDS tests, or received iron supplements (28). Only seven women (1%) reported receiving all 16 ANC services from the public sector; no one received all 16 ANC services from the private sector (28). While follow-up care and comprehensive ANC, as recommended by WHO, were an essential component of effectiveness, over-utilization of care had no impact on improved birth weights in a study of six Central and South American counties (29). A comparison of public and private maternity services in India evaluated components of care based on WHO's labor guidelines, finding that both facility types conducted non-evidence-based procedures uncompliant with effective guidance (30). A study of care received by women of reproductive age in India found that 34% underwent a non-evidence-based episiotomy (31).

Similarly, an examination of ANC care in 46 LMICs, using Demographic and Health Survey data, found that the mean score of individual components received was 0.28, with private not-for-profit scoring 0.71, private commercial 0.63, and public providers 0.59 (32). In Uganda, coverage of ANC components differed between public and private sectors, with women receiving an average of 4.8/8 measured components: women received an average of 4.9 components in public facilities and 4.2 components in private facilities (33). However, these were not significantly different. Of the women who received all eight components of care, the proportion was higher for women in the private sector (17.5%) than in the public sector (8.5%) (33). In maternity facilities in India, providers in private facilities conducted 45% of recommended clinical practices compared to providers in public facilities who conducted 33% of recommended clinical practices, a statistically significant difference (5, 34).

The type of provider was also significant in the components and comprehensiveness of care received. In Afghanistan, researchers observed differences in effective care between providers, with physicians providing 2.9 greater odds of quality care than other providers (35). In two linked studies of reproductive healthcare in India, providers who received less medical training were less likely to provide effective care (36). In contrast, well-staffed abortion facilities whose providers had medical degrees and modern equipment ranked the most highly qualified (37).

Provider knowledge about components of care necessary was essential for effective treatment. In India, a study of obstetric emergency care quality across private and public facilities found that providers at higher facilities performed better than public health centers in knowledge of uterine anatomy, performing speculum examinations, knowledge of hemoglobin investigations, and monitoring vital signs (38). In Uganda, public facilities delivered more comprehensive newborn care, with 42.8% of newborns receiving at least eight essential care practices compared to 27.5% of newborns in private facilities (39). In Uganda, treatment of pregnant women according to guidelines occurred at 40.7% of private clinics, 28.2% of drug shops, and 16.7% of pharmacies, with knowledge of people vulnerable to malaria and the availability of a malaria treatment guideline associated with the correct treatment of pregnant women with fevers (40).

Moreover, the effective dissemination of knowledge to care-seekers was reported to ensure effective care. An assessment of the quality of clinical care in Sri Lanka found that public-sector facilities performed better at taking the histories of people seeking care, investigation and management, and examination than private facilities (41). In contrast, private facilities were likelier to provide quality education and information. An evaluation using interviews of clinical franchising programs in Kenya and Ghana indicated that franchise staff conducted tests and procedures that gave care seekers more confidence and prescribed effective medicine, particularly in the case of child sickness (42). Women who used private antenatal care services in Istanbul reported receiving more information about their pregnancy and fetal development than in public hospitals. However, observers of these visits reported lower counseling and information provision than women themselves (43).

Two studies reported on immunization coverage, categorized as a component of effective care. An evaluation of payments by performance among providers in Haiti found that the model of care increased immunization coverage from 13% to 24% (44). A comparative study of Benin, Malawi, and Georgia that explored vaccine provision and ineffective provision found that most facilities offered the full range of NIP vaccines in Malawi and Georgia (45). In contrast, 88% of facilities in Benin provided vaccines appropriate for infants under 6 months, and 70% of facilities provided vaccines appropriate for children older than 6 months (45).

#### 3.2.2 Training, guidance, and technical quality of caregiving

Studies that explore the impact of technical quality found that effective care varied significantly across facility types. However, this was inconsistent, as a study of the effectiveness of public and private reproductive health services in India found that the same proportion of care-seekers in both (44.4%) continued utilization of facilities due to effective care (46).

A comparative study of effective training, guidelines, and knowledge among different facility types in Zambia found that community-based agents performed poorly in providing basic medicines and management (47). At the same time while pharmacies had better medicine availability, they failed on other measures of QoC (47). Shops and traditional practitioners performed poorly across all types. A multi-country review of mission hospitals found that compared to government facilities within the same districts, mission facilities in Malawi, Uganda, and Ghana outscored their government counterparts in the use of standard protocols for laboratory tests, prenatal care, pelvic and other physical assessments, and use of partograms (48). In Guatemala, areas with a mixture of provider delivery models had higher quality prenatal care than regions with direct provider models (49).

Interventions aimed at improving the technical quality of caregiving had mixed results. As an outcome of a four-week course designed to enhance QoC through a clinical skills refresher course, community midwives in Pakistan reported significantly higher competency scores relating to their effective care than those in non-project areas (96.6% and 34.5%, respectively) (50). Among abortion providers in Bangladesh, a training program and regular monitoring visits led to 94% of NGOs providing uterine evacuation with recommended technologies by end-line, compared to 67% of public facilities and 33.3% of private facilities (51). A training program intervention to extend and sustain MNCH services in Georgia found that 51 respondents from participating institutions reported being offered effective newborn care, compared to 31 respondents from non-participating facilities (52).

In Papua New Guinea, respondents cited that training changed emergency obstetric care practices, such as improving the availability of ANC services (53). Observed technical QoC was higher among midwives who self-reported high technical competency in an intervention in Uganda, which ran a one-day training and self-assessment program to improve effective care (54). An accreditation program in the Philippines found that pediatric providers who undertook the intervention had improved their responses to vignette-based assessments of effective caregiving (55).

The nature of training and supervision programs on effective QoC is important. An intervention in India that aimed to support Sky program facilities found inconsistent implementation in training, with only 45% of providers receiving training (56). This inconsistency limited the effectiveness of the intervention. Before this intervention, many SkyCare providers had limited experience working in mental health (57). Similarly, an intervention to train nurse-midwives in Kenya was limited in effectiveness by sufficient post-training support, which impacted infection prevention procedures (58). Moreover, an intervention to improve QoC among private midwives in Uganda found that the presence of guidelines and job aids increased significantly in control clinics and clinics where only midwives were trained than in intervention clinics where midwives and supervisors were trained (59).

An intervention that aimed to strengthen healthcare through results-based financing found that there was no significant association between the occurrence of an emergency Cesarean-section and complete monitoring during labor through the intervention (60). Later assessments of the intervention found some improvements in clinical assessment in the results-based financing clinics, but these improvements declined slightly and were mixed in input-based funding clinics (61, 62).

Other elements of effective care referred to structural components, including human and physical resources, and effective medical practices. An analysis of 86 facilities in Malawi found that both public and private facilities had a shortage of doctors and Clinical Officers; however, private facilities were able to perform better (63). In clinics involved in the Gujarat Health Systems Development Project intervention in India, good physical infrastructure and provisions facilitated effective caregiving (64). Providers at these public facilities were also likelier to report being overworked and struggling with the patient load.

A comparative study of health care providers in Lesotho found that at government clinics, there was less effective triage and poor accessibility to emergency care (e.g., crash carts) in comparison to hospitals managed through public-private partnerships (65). Another comparative study of contracted rural health centers and government rural health centers found that contracted facilities had better equipment and quality, though not staff capacity, training, or knowledge, and thus no differences in the technical process of service provision (66). A case study of healthcare provider quality in maternity homes in Tanzania found evidence of basic drug and equipment shortages in both public and private sectors, impacting the ability to provide quality ANC (67).

Another measure of effective care included hygiene practices: a study of 85 public and private hospitals in India used hand hygiene practices to measure effective infection prevention, finding that 12% of public and 44% of private providers had hand hygiene compliance (68).

### 3.3 Efficiency

Only four studies reported on efficiency as a component of quality care (Table 7). Two of these studies reported on interventions that impacted the efficiency of care.

**Table 7:** Quality of care measures and metrics organized by the efficiency domain.

Domain (*n*)	Variable measured	Metric(s) used	% of studies under the domain that measured the variable (*n*)	Most common year of publication in five-year windows (*n*)
Efficiency (*n* = 4)	Economic efficiency	Economic efficiency of providers of care per capita	25% (1)	2005–2010 (1)
	Antenatal care (ANC) efficiency	Pre-ANC consultation services score, ANC service readiness, whether family planning counseling is routinely conducted during ANC visit	25% (1)	2005–2010 (1)
	Efficiency of care	Service provision delays, patient wait times	50% (2)	2015–2020 (2)

Through the SHOPS program, that aimed to improve and capacity and quality care in health services in Malawi, there were improvements in the management of collecting blood samples and in the restructuring of the outpatient department, which reduced patient waiting times (69). This was achieved through a coupon system that operated on a “first come first served” approach. In a health facility strengthening intervention in Bangladesh, researchers found that information on where to go for pregnancy-related complications increased significantly with capacity building and service provider orientation (70). Moreover, the introduction of vouchers reduced the proportion of women reporting service provision delays.

Two further studies reported on differences between facility types—in an analysis of the 2010 Kenya Service Provision Assessment, researchers found that within management authorities, faith-based organizations performed consistently well in relation to their efficiency (71). In a study of the national expansion of health services in Guatemala, health posts were the most economically efficient providers of care per capita for the populations they cover than other service providers (49).

### 3.4 Equity

Twenty-two studies reported on equitable quality healthcare (Table 8). The majority of these studies (*n* = 11) focused on equitable care for people across different socioeconomic or sociodemographic strata. Ten studies explored the differences in equitable care based on regions and geographic access to different facilities, with additional studies considering equity between facility types.

**Table 8:** Quality of care measures and metrics organized by the equity domain.

Domain (*n*)	Variable measured	Metric(s) used	% of studies under the domain that measured the variable (*n*)^a^	Most common year of publication in five-year windows (*n*)
Equity (*n* = 24)	Geographic access	Travel time to clinic, urban/rural difference, regional immunization rates, quality of care in rural areas, access to care in rural areas	45% (10)	2005–2010 (5)
	Socio-economic status (SES)	ANC use by SES, ANC quality score same at public/private facilities, poor vs non-poor access to care (% that quality as poor, wealth quintile), SES	36% (8)	2010–2015 (4)
	Skin color (secondary)	Skin color paired with SES	5% (1)	2010–2015 (1)
	Age (secondary)	Age paired with SES—Number of youths (15–25 years) accessing RHS	5% (1)	2015–2020 (1)
	Patient sex (secondary)	Patient sex paired with SES	5% (1)	2010–2015 (1)
	Unspecified	Terms such as “accessibility”, “equity in receipt of procedures”, “equity in having a skilled birth attendant”, “equity in use of ANC”, “doctor does not discriminate among patients”	27% (6)	2015–2020 (3)

^a^ The number of data points in column 4 (*n* = 27) exceeds the number of studies that reported on this domain (*n* = 22), as some studies examined multiple variables for equity.

#### 3.4.1 Socioeconomic and sociodemographic factors

Many studies (*n* = 11) reported on the impact of socioeconomic and sociodemographic factors on the QoC received, often illustrating the inequities between different groups in the care that they receive. Such differences were observable between facility and sector types, within facilities, as well as based on provider demographics in addition to care-seekers.

An assessment of 25 health facilities implementing basic health services in Afghanistan revealed that poorer people received higher level of quality care than non-poor in non-governmental facilities, while no differences were observed in relation to government facilities, remoteness, facility type, provision of payments, and training (72). Among urban health centers in India, care was primarily provided for people who were in poorer wealth quintiles, although there were inconsistent eligibility criteria for free services across services (73).

An assessment of outpatient care for children under five at hospitals in each provincial capital in Afghanistan found that when controlling for hospital type and patient sex, the odds of quality care provided by a female provider compared with a male was 5.8 times higher (35). Moreover, among abortion providers in India,.

An analysis of quality care in a nationally representative survey in Indonesia found that in Java-Bali, there were significant differences in care based on wealth quintile, with the poorest households having access to higher-quality prenatal care (74). These differences were not observed in Outer Java-Bali. Within private clinical settings in Mexico, the poorest quartile received significant fewer procedures compared to the wealthiest, wherein increase by one quartile is associated with a 5% increase in procedures received (75). Using DHS and AIDS Indicators Surveys, wealth quintile was related to whether a person was offered an HIV test at ANC check-up, although this was inconsistent across the 18 country contexts examined (76).

An intervention that aimed to improve maternal health in the Philippines through an accreditation and reimbursement program found increased accesses to services among poorer women under the intervention program, with more women accessing family planning and maternal and child health services at private birthing homes without financial constraints (77). The NGO Health Service Delivery Project, which aimed to strengthen NGO capacity to delivery family planning and reproductive and MNCH care in Bangladesh, in order to increase access for poor and underserved populations, reported an increase in 35% of users qualifying as poor in the first year to 42% in the third year (78). The Rural Service Delivery Partnership, a USAID intervention in Bangladesh to improve MNCH care, found that the gap between wealth quintiles access antenatal care was 10 percentage points lower in RSDP areas than control areas, where rich women were three times as likely to use ANC as women in the poorest socioeconomic quintile (79).

Interventions that aim to address inequities were not always successful. An evaluation of three maternal health social franchises in Uganda and India found that although the aim was to serve poorer groups, the users were concentrated in higher wealth quintiles, though content of care did not vary by socio-economic status (80).

#### 3.4.2 Geography and accessibility

Whether a facility was rural or urban, accessible by public transport or on foot, and the distance to facilities create inequities that impact the QoC provided.

The Bangladesh Smiling Sun Franchise Program Impact report found that in rural areas, the poorest quintile experienced the greatest increase in ANC coverage between 2008 and 2011; in 2008 the richest quintile was 2.7 times likelier than the poorest quintile to seek ANC, which had reduced to 1.9 times by 2011 (81). This was similarly observed in comparison areas. Within urban areas, the Smiling Sun provider had a market share reduce from 27% to 24% over the course of the intervention program (81).

A comparison of growth and monitoring programs in Côte D’Ivoire found that the rural government program rated low on technical procedures, though the proportion of respondents who declined to rate the program was twice as high in rural than urban areas (82). In an assessment of prenatal care in four regions in Guatemala, immunization rates in Totonicapán were significantly lower than other regions, while those children in mixed provider catchment areas had better immunization rates than those in direct provider catchment areas (49).

Geographic and accessibility impacted the choices that care-seekers felt they could make. Private clinic users in a study in Kenya reported that their reason for using private clinics over government clinics was better geographic access (83). The NGO Service Delivery Program, implemented in Bangladesh to provide family planning and MCH care, found that travel time to NDSP clinics was 13.1 min, while users of government facilities reported local travel times (22). In addition, accessibility as availability was also cited by care seekers as a reason for using private midwives in an evaluation of an intervention in Uganda, though the proportional increase from baseline to endline was not statistically significant (84). While there was no difference in care based on socio-demographics, QoC among maternity facilities in India was higher on weekdays than weekends (5).

Geographic disparities also created inequities within provider staffing. A case study of maternity home healthcare providers in Tanzania found that an aspect that compounded geographically inequitable care was low staff motivation and high job vacancy rates in rural facilities compared to others (67).

#### 3.4.3 Facility type

Comparisons between facility types and sectors illustrated some of the manifestations of inequities, though these varies depending on the context and care being sought.

A study of ANC provision in Kenya and Namibia indicated that there were no significant different in ANC quality care by facility type (public or private) in either country, although private facilities performed better in Kenya on EmOC and EmNC care, while there were no significant differences in Namibia (85). In Indonesia, private physicians provided above-average quality care in both Java-Bali and Outer Java-Bali, with the exception of prenatal care in the latter, while public facilities offered above-average care in Java-Bali but below average in Outer Java-Bali (74). In a comparative analysis of quality of maternity care across 57 countries, the percentage point difference in having a skilled birth attendant in public hospitals was 3 between poorest and richest wealth quintiles, compared to 2 in private facilities (2). The proportion of births to women in the poorest quintile attended by a doctor was 63% in private facilities and 45% in public facilities.

The relationship between care seekers, type of facility, socio-economics, and geography are interlinked. A study of post-abortion care in the private sector in Pakistan indicated that most poor women went to private and not government hospitals to receive post-abortion care, due to treatment from staff, lack of medicines, and the availability of private facilities at a convenient distance (86). This relationship was also found in a study of birthing experiences in Brazil, where socio-economic and racialized differences in attendance of public and private facilities were measured by differences in attendance rates to certain facilities (87). Women from lower incomes who were attended sterilization and abortion facilities in different primary health center locations in India had their facility choices curtailed due to the cost of care at private facilities (36). Cost of services also led women seeking abortions in India to seek care from community based providers who were less qualified (37).

### 3.5 People-centered care

People-centered care accounts for the preferences, aspirations, and needs of individual service users, as well as the cultures of their communities. Forty-four studies reported on people-centered care; primarily themed around client satisfaction, as well as good care practices, acceptability of care, and people-centered advice (as outlined in Table 9).

**Table 9:** Quality of care measures and metrics organized by the people-centered care domain.

Domain (*n*)	Variable measured	Metric(s) used	% of studies under the domain that measured the variable (*n*)^a^	Most common year of publication in five-year windows (*n*)
People-centered care (*n* = 44)	Continuity of care	Continuity of care, good follow up care, continuous care	7% (4)	2005–2010 (2)
	Client perception of quality	Patient preferences and expectations; quality of services as rated by users; perceptions of quality; perceptions of how polite, friendly and caring the providers and other staff were	17% (9)	2015–2020 (5)
	Patient as an active participator in care	Lactation advice, family planning advice; client discussions, patient acceptability; provider participation in public health and women's organization activities at the community level; mother awareness of growth chart, value of advice; mothers’ and women's’ knowledge; provider-patient communication; education; mothers allowed to eat/change positions during labor; communication to mothers; not forced to accept sterilization	24% (13)	2000–2005 (4)
	Quality of interactions/care	Polite and kind care; privacy, fair charges, good handling of clients; privacy; attitudes of staff, professionalism; receipt of individual newborn care practices; adequate pain management, privacy, non-judgement, attentive; enough beds, privacy; presence of abuse; quality of contact	22% (12)	2000–2005 (3) 2010–2015 (3)
	Counselling	Counselling score; whether received counselling or not	11% (6)	2005–2010 (2) 2010–2015 (2)
	Unspecified		15% (8)	2015–2020 (5)

^a^ The number of data points in column 4 (*n* = 52) exceeds the number of studies that reported on this domain (*n* = 44), as some studies examined multiple variables for people-centered care.

#### 3.5.1 Client satisfaction

Treatment and manner of support staff during care was a significant predictor of satisfaction for women attended child health services, for example, in an analysis of a public-private partnership healthcare facility in India (88). This was also found among women attended maternal and child health services in Kenya, who associated positive quality was clinician attitudes, preferring private facilities (89). An assessment of Guatemala's program to extend basic health services used problem resolution, provision of medicines, waiting more than one hour, and friendliness of services to measure client satisfaction, finding more than 70% of women responded positively to these across all types of providers (49). A study of public and private facilities in India found that QoC indicators that related to patient-centeredness, such as being allowed to eat or change positions during labor, were significantly better in fee-paying, private care, than free care (31).

Client satisfaction and perceptions of people-centered care was cited by respondents in a study of maternity care in Dhaka, Bangladesh as a reason for using private over public hospitals (90). Among post-abortion care providers in Kenya, staff professionalism was associated with choice of health facility among care-seekers (91), and care-seekers in Ghana and Kenya reported using franchised care providers due to higher satisfaction with care through staff interactions, cleanliness, and shorter waiting times (42). Among women seeking maternity care in Kenya, perceptions and experiences of bad interactions and treatment was cited as a reason to avoid government clinics (67).

A number of interventions in this review were designed to improve client satisfaction and user friendless and staff behaviors as a key indicator of QoC. An intervention of private nurse and paramedic services in Nepal, through 7 days of training, increased client satisfaction with the physical appearance of clinics compared to control clinics, while not reporting higher satisfaction with client handling or service charges (92). The introduction of a 3.5-day training orientation with maternal health service providers to improve QoC, and an awareness raising of the intervention communities in Bangladesh found that women reporting being treated in a friendly manner increased to 96% at end-line, and information provision increased significantly (70). From the provider perspective, the abolition of user fees in child health services in Niger was reported by 64% of workers to have had positive impacts on how they treated patients (93). An intervention aimed to accredit private sector health facilities providing abortions in India reported that 46% of clients were highly satisfied with the services, compared to 44% moderately satisfied and 10% with had low rates of satisfaction (94).

Three USAID interventions in Bangladesh were evaluated based on care seeker and provider interactions. The Rural Service Delivery Partnership evaluation reported that service users rated good or very good staff behavior (99.3%) and quality of services (98.7%), though these were comparable with non-intervention areas (79). The urban NGO Service Delivery Program reported that 93.7% of service users reported staff spent enough time with them, 95.8% that they paid attention to their needs, and 85.7% that staff talked to them nicely (22). The rural NGO Service Delivery Program found that nine in ten service users reported that staff talked to them nicely and paid attention to their needs, similar to non-intervention areas (95).

Findings of positive patient-centered care were not consistent across the studies included in this systematic review. There were no significant differences among clinic types and sectors in communication and assessment with care-seekers among MNCH facilities in Pakistan (66). Clinical observations of maternity and childbirth care centers in India recorded that only 4% of deliveries provided people-centered, respectful care (34). In a comparison of public and private primary care providers, the quality of provider communication was lower for poor than non-poor care-seekers in government facilities, whereas no difference was observed in non-governmental facilities (72). For unmarried women obtaining abortion services in Istanbul, satisfaction with the procedure, interactions with medical personnel, and cleanliness was higher in private facilities than public facilities, with women reporting feeling judged in the latter (96).

#### 3.5.2 Good care practices, counselling, and advice

In order to provide people-centered care, the provision of counselling and advice, including education and knowledge dissemination/information access, as well as broader practices of good care, are essential. This includes ensuring continuity of care, for improved outcomes and to center the need for long-term, regular care for healthcare, e.g., antenatal care.

The evaluation of the Delivery of Improved Services for Health project, Uganda, reported that private providers were likelier to encourage people to discuss their treatment compared to government and NGO facilities, while NGO and private facilities continue to have low quality privacy during care (97). Privacy and client treatment was used to measure people-centered care in public and private facilities in a study of reproductive health services in India: 39.5% of public and 38.6% of private care seekers continued care due to client treatment, and 33.9% of public and 40.9% of private care seekers continued care due to privacy (46). An evaluation of case records in a cross-sectional study of maternal and neonatal health in Bangladesh, the proportion of facilities that used bed side screens (55%), comment boxes (32%), BCC materials (15%), receptionists (100%), and service prices displayed (15%) were used as people-centered indicators (98). Patient-centered care was rated best amongst faith-based organizations compared to other management authorities in antenatal care clinics in Kenya (71).

Moreover, an evaluation of mother's perceptions of quality growth and monitoring programs in Côte D'Ivoire reported more complains on technical procedures in government-run programs than in NGOs, and mothers reported valuing care advice from NGOs, while mission hospitals were the only facilities that reported taking child life histories as part of care (82). Relating to community-centered health practices, an intervention that promoted provider participation in public health activities at the community level led to an increase in providers attended women's organization meetings, covering healthcare topics including diarrhea treatment (26).

Good care practices also included advice given to care seekers in relation to their healthcare. The proportion of women who received prenatal care in rural Mexican communities who received advice on lactation was 92.24% in social security facilities, 92.23% in IMSS Oportunidades facilities, 91.30% in government facilities, and 71.04% in private facilities (99). A baseline assessment of hospital care in Afghanistan found that 18% of consultations involved counselling caretakers about feeding during child illness (35). A higher proportion of women attended private (43%) than public (18%) facilities in Pakistan received information regarding pregnancy, delivery, and postnatal complications, with no clinics in either sector providing education and advice for newborn care (100). Among women seeking private or public maternity care in Istanbul, there was no significant difference in overage of essential newborn care practices with the exception of immediate breastfeeding (39).

Furthermore, continuity of care was a proxy component for people-centered care, as it ensures that the needs of care-seekers were prioritized. Through the introduction of an accreditation program using reproductive health vouches in Kenya, the adjusted odds of continuous care for births was 1.5 times higher in intervention than comparison counties (101). An evaluation of a maternity quality improvement package and intervention in Uganda similarly used continuity of care as a measurement of success, though found no significant differences between intervention and control (102). Continuity of care, in the form of being able to request the same provider across ANC visits, was cited as a reason women sought care from private hospitals in Istanbul (43).

Interventions to improve counselling and advice had mixed results. An evaluation of a program to improve MNCH care in India had three factors that related to people-centered care: contacts with service providers, advice received, and services received. While quality of contact was higher in the intervention areas than comparison, less than 25% of women were given advice on breastfeeding (103). An assessment of a microfinance intervention with private sector midwives in Uganda found that despite increased proportions of people reporting satisfaction with privacy, accessibility, charges, and drug availability, these were not significantly different from the baseline (84). A further evaluation of a quality improvement package for private midwives in Uganda found no differences in the continuity of care between intervention and private clinics (59).

Structural components of care that care seekers prefer are also important. A comparison study of private and public antenatal care providers in Tanzania found that 29% of public and 35% of private facilities had toilets with water to flush, 43% of public and 78% of private clinics had waiting places, while 100% of all clinics had private examination rooms (104). Women seeking abortions in India, the requirement of sterilization in some government clinics meant women chose alternative facilities in order to avoid conditional care, though this policy has shifted (36). For other women seeking abortion care in India, negligent care and poor follow up was associated with complications at government and intermediate facilities (37). For maternity care providers in Guatemala, resource limitations and high patient load in government facilities compared to the Casa Materna created frustration among care-seekers (105).

A number (*n* = 6) of studies use counselling on contraception as a measure of QoC. An intervention evaluation of post-abortion family planning in Ghana saw an increase in women counselling from 55% at baseline to 61% at endline, with a greater proportion reporting that providers asked them about their contraceptive histories (106). Another study in Kenya that trained private nurse-midwives in post abortion care reported 81% of respondents receiving counselling for family planning post intervention (58). Of women receiving ANC in private and public hospitals in Pakistan, 13% got information on family planning at public facilities, whilst only 7% received advice from private clinics (100). Women attending prenatal care in Mexico had different advice depending on the facility type: 90.17% of women who attended social security facilities were advised on family planning, compared to 91.85% in IMSS Oportunidades facilities, 85.72% in government facilities, and 55.21% in private facilities (99). Two comparative studies of health service delivery programs in Iran found that women who attended cooperative health centers had higher knowledge about family planning than those who attended public health centers (107) and more suitable education (108).

### 3.6 Safety

Forty-five studies reported on safety as QoC (Table 10). Studies reported on a number of different indicators and components of care that relate to safe QoC: attendance by qualified healthcare professionals and supervision, clinical and case management practices, healthcare coverage, correct testing and treatment, and the healthcare environment.

**Table 10:** Quality of care measures and metrics organized by the safety domain.

Domain (*n*)	Variable measured	Metric(s) used	% of studies under the domain that measured the variable (*n*)^a^	Most common year of publication in five-year windows (*n*)
Safety (*n* = 45)	Appropriate materials	Presence of enough skilled manpower (e.g., supervision); presence of enough raw materials (e.g., blood banks close by, anesthesia, minimum desired number of BEmOC facilities per population); quality of HIV testing	15% (7)	2015–2020 (6)
	Clinical competence	“Clinical competence”; adequate provider ARI disease management practices; minimal complaints on technical procedures; technical quality; appropriate guidelines available; compliance to standards; correct treatment administered; safe MR procedures; accreditation of doctors; effective monitoring and regulation in place; number of patients with incomplete/complete abortions; number of surgical procedures; correct antibiotic administration; no sepsis cases; no ICU care; documentation of care; clinical vignettes; rate of institutional birth	52% (24)	2015–2020 (12)
	Hygiene	Facility cleanliness; infection prevention practice; effective waste disposal; provider wash hands before care; 14 essential hygiene equipment present; running water	21% (10)	2015–2020 (6)
	Unspecified		13% (6)	2015–2020 (3)

^a^ The number of data points in column 4 (*n* = 47) exceeds the number of studies that reported on this domain, as some studies examined multiple variables for safety.

#### 3.6.1 Qualified healthcare professionals

The presence of qualified or skilled healthcare professionals was a major component of safe QoC reported in studies. This included the proportion of people seeking care within medical settings, alongside the impact of training on providers of care.

A study examining the quality of free delivery care in India indicated that more than half (57%) of the deliveries were assisted by nurse or auxiliary nurse midwife, with gynecologists attending 22% and general doctors and unqualified personnel attending 10.5% of deliveries respectively (31). A piloted performance-based payment intervention in Haiti found that NGOs had a 17%–27% increase in attended deliveries, and a 13%–24% increase in immunization rates (44). Moreover, an intervention that used public-private partnerships and community health workers to improve women and child's health in Nepal reported that institutional births, which were taken as a proxy for safe care, increased from 80.7% to 92.5% (109).

In their study of the technical quality of maternal healthcare across two regions in Nigeria (110), found that safe practices varied significantly by facility type and across regions. In Enugu, private not-for-profit comprehensive emergency obstetric care facilities scored the highest on the Safe Attendance Index compared to all other facilities types—including basic emergency obstetric care units either private or public. These differences were smaller in Lagos, with private for-profit public health centers outperforming public and private not-for-profit public health centers.

Lack of skilled attendance was a measure of a lack of safe quality care. In two linked studies of abortion providers in India, the least safe abortion-related care practices were evidenced among unqualified and unskilled providers, while no government facilities were rated as highly qualified and safe abortion providers (36, 37). Through clinical observations of maternity and child health facilities in India, 59% of deliveries had unqualified personnel attending—65% in the public sector and 41% in the private sector (34). Additionally, an analysis of QoC in public and private maternity providers (30), reported that unskilled women helped attend childbirth and the running of the wards in public facilities.

Furthermore, among community midwives in Pakistan, an intervention providing competency-based trainings led to significant improvement in the quality of antenatal, labor, and delivery and postnatal care, compared to those that did not receive training (111). Similar results were found among public-private partnership led clinics, but not in public or private facilities.

#### 3.6.2 Supervision and monitoring

Supervisory and monitoring activities were also reported across studies as an indicator of safety, although there was no consistent evidence that these did lead to safer QoC. A comparative study of vaccination services in Benin, Malawi, and Georgia, used supervision and monitoring as an indicator for safety, and reported that most private facilities were accredited by regulatory bodies, though there was more variation in regulatory visits in Georgia (42% in the last year) than Benin (84% in the last year), and that supervisory visits were least regular in Benin (54% monthly or quarterly) and most regular in Malawi (78% monthly or quarterly) (45). An intervention of open health centers in Papua New Guinea reported that as a result of the emergency obstetric care training there were more supervised deliveries, and that these also increased after renovations to the health facilities (53).

The Matrika program, which aimed at improving maternal healthcare in India, reported that supervisory monitoring did not have an impact on the safe QoC at intervention facilities (56). By contrast, an intervention involving a quality improvement package with private midwives in Uganda, found that changes in safe quality care practices were only achieved in the intervention group where midwives and supervisors received training, compared to the control group and the intervention group with only midwife training (54, 102). A quality management intervention aimed at increasing contraceptive uptake in Kenya reported that supervision visits and regular discussions between providers and supervisors were linked to better quality assurance (106).

#### 3.6.3 Clinical and case management practices

Clinical and case management practices were reported in either using existing quality standards of care for medical procedures, or by reporting on the prevention of complications and secondary illnesses.

Six studies used specific quality standards to measure the safety of clinical practice (25, 26, 112–115). In their study of 64 healthcare providers that offer obstetric anesthesia in Uganda, only three facilities fulfilled the international standards for safe anesthesia practices (112). Using WHO care standards as an assessment tool, a study of ARI disease management practices in India indicated that rural private providers scored 8 out of 33 on expected disease management practices and was considered “inadequate” by the evaluators (25). Of clinics providing HIV testing and counselling through the APAIDSON program in India, 100% had concordance with EQAS HIV testing quality (113). Three hospitals providing HIV related services in Malawi were assessed against national standards for infection prevention, of which two scored over the necessary 80% (115).

Studies also used correct treatment as a measure of safety. An assessment of inpatient clinical care in public and private facilities found no differences in safe quality scores, including correct treatment and ICU care indicators (116). An evaluation of clinical management of child healthcare in Mexico found that half of the private and 7% of public GPs gave the wrong rehydration scheme in the management of diarrhea, and 64% of private GPs gave incorrect dietary advice compared to 13% of public GPs. 66% of private and 27% public GPs gave wrong antimicrobial prescriptions and in 49% of cases private GPs gave unjustified prescriptions, compared to 3% in public sector (117). A further study of care provision in Mexico among parents who had experienced the death of child indicated that 52.9% of doctors were implicated in contributing death, and private doctors were likelier to be implicated than public (118). Among newborn services in Nairobi, incorrect doses of antibiotics were prescribed to 19.4% of newborns, with gentamicin accounting for 11.7% of these, while safe equipment and drugs for mothers in the delivery ward were the weakest domains of a quality assessment of neonatal services (119, 120). An intervention that trained and promoted community health promoters in Uganda led to a 17% increase in treatment of diarrhea with oral rehydration and zinc and a 54% increase in follow-up care for ARI, malaria and sickness in children (27).

Various interventions aimed to enhance safe care practices. In the Philippines, an accreditation and insurance program involving public and private doctors revealed that insurance payments had a greater impact on the quality of private doctors than accreditation did, especially in providing safer quality recommendations (55). A Quarterly Quality Assessment of a results-based financing intervention in India demonstrated significant improvements compared to input-based funding. People were three times more likely to be treated correctly for malaria, seven times for pneumonia, and eight times for diarrhoea, though confounding effects were not fully controlled (61, 62). In a study of 31 hospitals providing MNCH care in Afghanistan, caretaker counselling resulted in 56% of caretakers providing correct responses on administering medication at home. The overall Quality of Care (QoC) score was 27.5 out of 100 (35). The Health Facility Survey evaluation indicated that more district and upazila public facilities (46%) reported quality assurance activities compared to NGO facilities (29%) and private facilities (21%) (121). Faith-based organizations in Kenya providing only antenatal care were rated moderately in safety compared to other management authorities (71).

Conducting recommended procedures, including managing and monitoring cases, was an important component of providing safe quality care. A study that assessed four growth and monitoring promotion programs in Côte D'Ivoire found that there were more complains among government-run programs than NGO programs, focused on the lack of technical procedures such as advice, investigation into the causes of growth failure, lack of immunization status checking, and no appointments given for the next monitoring program (82). An assessment of the QoC in the private sector in Uganda found that only 40.7% of women at private clinics, 28.2% at drug shops, and 16.7% at pharmacies were treated for fevers according to safe guidelines (40). In their observation of life-saving clinical practices and infection prevention in India, Sharma et al. (35) found that life-saving practices such as partograph use for monitoring labor, screening for pre-eclampsia/eclampsia, and active management of third stage labor were rarely observed.

Management practices relating to abortion care included ensuring a safe environment, provider hygiene, and safe treatment. A study of comprehensive abortion care in Nepal indicated that in government sites there was a need to improve safe management practices—such as sterilization techniques, instrument cleanliness, and waste management, in comparison to private providers (122). Furthermore, study of antenatal care provision in Istanbul, Turkey, found that 22.9% of providers at Ministry of Health hospitals, 0% at social service hospitals (SKK) and 5.5% at private hospitals washed their hands before or after examination (43). Finally, an intervention that trained staff and introduced a management tool among NGO, public, and private menstrual regulation (abortion) providers in Bangladesh found that NGOs performed 93% safe menstrual regulation procedures at endline, compared to public facilities (69%) and private facilities (33%) (51). An intervention to improve post-abortion care in Kenya through training private nurse-midwives on counselling, management of complications, procedures, resulted in reports of lower rates of incomplete abortions (58).

#### 3.6.4 Healthcare environment

The broader healthcare environment and structural aspects of the healthcare setting were identified as critical safety components. A study on emergency services in India revealed inadequate obstetric care facilities in 16 covered regions (123). Interviews with abortion recipients in Turkish facilities showed that private facilities were perceived as having better quality and cleanliness than public ones (96). Cleanliness served as a safety metric in a Ugandan intervention where loans were provided to private midwives; the intervention resulted in a significant decrease in the proportion of people attending comparison clinics due to cleanliness (84). In India, private maternity clinics lacked on-site or nearby blood banks, affecting their ability to deliver safe, quality maternity care (30). Conversely, in Pakistan, an intervention outsourcing MNCH care to NGOs indicated that contracted partners outperformed non-partners in waste disposal (66).

An assessment of facility readiness for obstetric emergency care highlighted disparities: 95% of private facilities and 78% of public facilities had highly functional operating theatres, but only 22% of private facilities had stationed ambulances, compared to 88% of higher public facilities (38).

### 3.7 Timeliness

The final component of QoC is timeliness, defined as reducing delays in providing and receiving health care. Thirty-one studies (Table 11) reported on timely quality care across three key themes: waiting times, service readiness and availability of care, and follow-up and regularity of care.

**Table 11:** Quality of care measures and metrics organized by the timeliness domain.

Domain (*n*)	Variable measured	Metric(s) used	% of studies under the domain that measured the variable (*n*)	Most common year of publication in five-year windows (*n*)
Timeliness (*n* = 31)	Wait times and service readiness	Laboratory turnaround times; number of days per week services were provided; waiting times; doctor availability; service readiness; availability of 23 h on-call pediatricians; availability of ANC services; vaccine availability; availability of clinical care and support services; weekend care; service delays; power cuts delaying operations	61% (19)	2005–2010 (6) 2015–2020 (6)
	Emergency care	Prompt response to emergencies; ambulances; the availability of an emergency crash cart (a wheeled cart stocked with equipment, supplies, and drugs for use in resuscitations); emergency referral	13% (4)	2005–2010 (2)
	Follow up visits	Home visits in the first seven postnatal days; early and regular ANC visits	16% (5)	2015–2020 (4)
	Unspecified		10% (3)	2015–2020 (3)

#### 3.7.1 Waiting times

Waiting times are significant for people seeking care, and are linked to their satisfaction with care and perceptions of QoC (49). Among women in Kenya, waiting types and patient turnover were cited as influencing the QoC and perceived professionalism at primary-level PAC services (91). Doctor availability and waiting times were critical factors in women's utilization of reproductive health services in India, with 40.7% and 38.1% of women continuing care due to availability and waiting times respectively (46). Private clinic users in Kenya reported that rapid treatment was an advantage of using private clinics for child healthcare, compared to government clinics (83). The introduction of vouchers streamlined services and reduced delays in services for people seeking care in intervention clinics in Bangladesh (70).

Evaluations of USAID funded NGO Service Delivery Program and Rural Service Delivery Partnership Program in Bangladesh found timely care critical. For the NSDP, waiting times were shorter in intervention clinics than government clinics, though longer than private clinics, while the majority of respondents felt that staff spent enough time with them (22). The RSDP evaluation survey found that waiting times were higher in government clinics than RSDP clinics, with 44.5% of users not having to wait for any services (79).

Fewer studies reported specifically on time spent in care, although this was noted in a study in Guatemala and Bangladesh as an indicator for timely quality care. A study of the partnership between the Ministry of Health, Guatemala, and non-public maternity care givers to establish a Casa Materna, had respondents report that the Case Materna offered longer stay and made the respondent feel less rushed to leave than previous hospital births (105). The evaluation of the Rural NGO Service Delivery Program, a USAID-funded intervention in Bangladesh, found that travel times and waiting times were longer at clinics in NSDP areas relative to government clinics in non-project areas, though most NSDP users said staff spent sufficient time with them (95).

#### 3.7.2 Service readiness and availability of care

Service readiness and increased availability of care could have significant impacts on the QoC received, with positive health outcomes for care-seekers. Through the Children's Heartlink First Hospital of Lanzhou University, children under five were able to access care earlier due to increased affordability, which improved mortality rates as care was more timely (124). The Zambia HIV/AIDS Service Provision Assessment Survey found that clinical care and support services were availability at 97% of facilities with little variation, while PMTCT services were available in 19% of facilities and likelier to be available at hospitals and urban health centers than rural health centers and other types of facilities (125).

Studies including a comparison between private and public sector facilities noted the variation between the two, which impacted the ability for people to seek care when they desired. A study comparing public and private abortion providers through the Nepal Health Facility Survey found that public facilities were likelier to have all required components of care than private facilities, while public facility readiness was low, no private facilities had all service readiness components for abortions (126). In a comparison of 9 cooperative health centers and 18 public centers in Iran, the cooperative health centers had significant better availability of services compared to public health centers (108).

Service readiness and availability of care was noted as particularly important for emergency care. Quality care as responsive times for emergency care in Tanzania were recognized by community, as mission hospitals gained a higher reputation for effective responses to EmOC (48). This led to faith-based organization hospitals to receive higher percentages of obstetric complication cases than government facilities. A study of eight districts in India found that 33.8% of higher public and 16.2% of private facilities had one specialist, while only 22% of private facilities had ambulances stationed for emergency care, compared to 88% of higher public facilities (38). A case study of maternity health providers in Tanzania found that no maternity home had a formal emergency transport plan, though some had established methods for referral (67).

Availability also included the time of day and day of the week that care was offered. A study of neonatal care in 266 hospitals in Brazil found that public facilities with NICU had poor 24 h availability compared to private clinics, though the latter had worst availability of medicine (127). In further studies, the QoC was impacted by the time of week, with poor quality care being reported during weekends at maternity facilities in India (34). Private facilities being open for longer and at night, compared to public clinics, was cited by MNCH care users as a reason to choose private care (89). An a three-arm intervention to improve private midwife care, the second intervention arm, which involved midwife and supervisor training, led to significant increase in days per week that services were offered compared to the first arm (training midwives only), and the control (59). Where facilities were more than two hours travel time in Afghanistan, there was an associated decrease in the odds of quality care of 2.1 compared to where hospitals were under two hours journey time (35).

#### 3.7.3 Follow-up and regularity of care

An important component of timely QoC that emerged in the systematic review was timeliness post initial care, particularly through follow up or regular care where appropriate. An intervention in private care providers of MNCH which aimed to increase tool-assisted counselling sessions for mother-child pairs found that 34% of pairs made three or more follow-up visits (of a recommended four), 54% came to 1%–2% and 12% did not attend any, with clinical staff reporting that follow-up calls was acceptable though occasionally not feasible due to inconvenient appointment times, lack of phone ownership, or non-desire of mothers (128). A training and capacity building intervention in Papua New Guinea resulted in increased availability of ANC care, including the ability for health care workers to ensure continuity of care and organize subsequent ANC visits among care seekers (53).

A number of interventions sought to increase follow up and regular care. The Living Goods and BRAC Uganda Community Health Promoter program, that provided visits, household education, referrals, and sold medicines resulted in households in CHP villages being 8.1 percentage points likelier to receive a follow-up visit one week after giving birth from any health care worker, an increase from 11.4 percentage points in the comparison group (129). In addition, households in CHP villages were 6.1 percentage points likelier to receive follow-up home visits after a child fell sick with malaria, an increase from 8.4 percentage points in the comparison group. This intervention was found a 54% increase in follow-up visits for under-five children who had malaria, ARI, or diarrhea (27). However, an intervention to support results-based financing among 21 private-not-for-profit facilities in Uganda found that after two years, the intervention had little impact on early and regular ANC visits (62).

While some studies found follow up care different across sector and facility types, this was not consistent. Women seeking abortion care in India reported that government clinics had limited follow up care and were slow in responding to post-abortion care needs, compared to non-government clinics, though the latter are frequently unaffordable (37). On the other hand, a comparative study of health service delivery of childcare in Iran found no significant difference across sector or facility type (107).

## 4 Discussion

By systematically reviewing QoC outcomes in private clinics, this article established a lack of consistency between private and public sector quality across the six QoC domains. For effectiveness, private and public sector facilities offered similar components of care that, in some cases, were ineffective regardless of the facility type (25, 26, 34, 60). Conflicting findings were noted; some studies reported more comprehensive care in the private sector, with better adherence to guidelines (40, 59), while others indicated public facilities operating more effectively in certain aspects of maternal care (28) and delivering superior newborn care (39, 52, 66), though such differences were not always statistically significant. Similar inconsistencies were observed in training, guidance, and technical QoC across facility types. In terms of equity, several studies found that poorer people received higher QoC in countries such as India (36), Indonesia (75) and Mexico (75), yet this was not the case in countries such as Java-Bali (74).

Regarding people-centered care, numerous studies indicated that private facilities generally provided superior care, encompassing both structural components and the ability of patients to seek counselling and advice (66, 70, 91), compared to public facilities (9, 117, 130). This trend wasn't universal; no significant difference existed in communication with care-seekers across clinic types in Pakistan (100). The private sector was considered “safer” in certain respects, such as having high-functioning operating theatres, while being“less safe” in others, like having fewer stationed ambulances than public facilities (36–38, 63). In matters of timeliness, patients experienced shorter waiting times in the private sector (45, 53, 91, 129). However, when assessing readiness of care and follow-up care, public facilities either outperformed private ones (27), or no statistically significant differences in timeliness between facility types were observed (107).

When viewed together, findings from this systematic review show that there is minimal link between the type of facility (private, public, NGO, faith based) and quality of MNCH care, and confirms findings from other studies that private sector care is not necessarily performing well enough to be currently considered as contributing towards MNCH and UHC goals (9, 117, 130). It is therefore important to take lessons from when and where QoC was high in any facility, and where applicable, transfer these to other types of facilities in order to promote and enable equitable MNCH outcomes in aspiration of healthcare goals.

Private sector care's variable quality in delivering MNCH services is unveiled through a nuanced analysis of the six QoC domains. This breakdown allows a comprehensive understanding of QoC, revealing the interplay among domains and their subcategories. For instance, training and guidelines contribute to technical competence, ensuring safety in clinical practice. Reducing wait times enhances efficiency and people-centered care perception. This holistic view emphasizes the interconnectedness of QoC domains and their metrics, highlighting the need to consider them collectively rather than in isolation.

Within each domain, there was significant variation between which years each subset was most frequently referred to. The most variation is found within the People-centered care domain: continuity of care was most used as a metric for this domain from 2005 to 2010, client perception of quality from 2015 to 2020, patient as an active participator in care from 2000 to 2005, quality of interactions was used the same amount from both 2000–2005 and 2010–2015, and counselling in both 2005–2010 and 2010–2015. The term “patient-centered care” pertaining to MNCH care has been found in studies dating back to 1994, with Dong et al. citing it as being widely recognized as a fundamental element of high-quality health care for patients, family members, and providers (131). Benefits span from increased quality of life, readmissions and length of hospital stay for patients; decreased stress and anxiety for family members; and improved job satisfaction, confidence, and reduced stress for providers (132–134). As patient-centered care is extremely multifaceted, it is understandable that this domain has been conceptualized slightly differently, yet remains prominent, in studies published over all five-year windows since 2000.

A 2020 literature review suggests that while the concept of people-centered care continues to be a focus of studies focusing on primary health care, the principle has not been demonstrated and absorbed on the ground, questioning the practicability and acceptability of the concept for nurses (135). Lateef et al. (136) cite that this gap is namely because of lack of training and knowledge of practitioners, which links to the training and guidelines subset in the “effectiveness” domain. Viewing these two domains together may therefore help patient-centered care achieve some of its intended benefits in practice and bridge this gap. This is consistent with the findings in our systematic review, as while the popularity of the notion of people-centered care is prominent (with 44 articles citing this domain), people-centered care was one of the few domains in which QoC was found to be higher in private facilities than public facilities (46, 82, 89).

Furthermore, under the effectiveness domain, while there was a lack of overall pattern, under the subset of performing to guidelines, private sector providers’ knowledge was higher than in other types of facilities (29, 40, 54, 59). The significance of this is twofold. Firstly, that viewing the domains at the subset level of granularity is useful for conceptualizing and understanding QoC, and secondly, that one way to improve equitable access to quality MNCH care is by ensuring adequate training and guidelines are provided specifically on providing people-centered care. A review of 28 systematic reviews published between 2011 and 2017 identified a variety of informational, educational and supportive interventions that can be used to achieve patient-centered care targeted at patients, family members, or providers (132).

It is important to recognize that many of these studies were published while QoC was becoming more clearly defined. In 2008, the extensive literature on health systems’ QoC was challenging to systematize (136). This complexity persisted in 2022, marked by a surge in interest since 2015 due to the SDGs. SDG 3.8, advocating for UHC and access to quality essential health-care services, firmly placed QoC on the global health policy agenda. The WHO, OECD, and World Bank collaborated in 2018 to publish a report and guide, emphasizing quality as integral to UHC aspirations (137). The period from 2015 to 2020 saw heightened interest in QoC domains, possibly spurred by the SDGs. Table 12 provides a summary of influential health quality definitions from various contexts between 1990 and 2018, adapted from OECD/European Observatory on Health Systems and Policies (138).

**Table 12:** Selected definitions of quality of care, 1990–2018.

Institute of Medicine (139)	Quality of care is the degree to which health services for individuals and populations increase the likelihood of desired health outcomes and are consistent with professional knowledge
Council of Europe (140)	Quality of care is the degree to which the treatment dispensed increases the patient's chances of achieving the desired results and diminishes the chances of undesirable results, having regard to the current state of knowledge
European Commission (141)	Good quality care is health care that is effective, safe and responds to the needs and preference of patients. Other dimensions of quality of care, such as efficiency, access and equity, are seen as being part of a wider debate and are being addressed in other fora.
WHO (142)	Quality health services across the world should be: • Effective: providing evidence-based health care services to those who need them. • Safe: avoiding harm to people for whom the care is intended. • People-centered: providing care that responds to individual preferences, needs and values.
	In order to realize the benefits of quality health care, health services must be timely […], equitable […], integrated […], and efficient […]

Adopted and adapted from OECD/European Observatory on Health Systems and Policies (138).

At present, QoC is currently one of the most frequently quoted principles of health policy at both international and national levels. However, there is disagreement about what the term encompasses, with definitions of “quality” differing across contexts and paradigms (6). As a secondary aim, and using the six domains of QoC as a guiding force, our systematic review intended to clarify how “quality MNCH care” has been defined in literature in order to ensure that these features are present across fragmented health systems. Ultimately, there needs to be a more concerted effort to define quality and use practical QoC measures in studies recording MNCH outcomes in different types of facilities. Understanding what QoC entails central to the success of the SDGs and the strive towards equitable health outcomes.

This systematic review was conducted as part of a larger study on private sector delivery of quality care for MNCH health in LMICs. The searches and screening were conducted together, and the findings and analysis supplement one another. For example, in the present systematic review, we decided to include both studies that focused on quality in the provision of care as well as studies that focused on quality of patients’ experiences of care. The decision to include both types of studies in the final inventory was based on our findings from one of the other papers in this study, which recognize patients’ experiences as crucial (if not a stronger determining factor) in MNCH-related decision-making than the quality of services provided (16).

This paper contributes to an emerging field of discourse on quality mixed health systems, recognizing that appropriate checks and balances are necessary to realize UHC goals. It is complemented by another paper in this systematic review series, detailing mechanisms for engaging the private sector in quality MNCH care (143). Furthermore, the WHO are carrying out broader efforts to effectively engage and govern mixed health systems, through their strategy on the governance of the private sector for UHC (144).

Several limitations affect the findings of this analysis. Due to language restrictions, we may have excluded relevant studies from Latin America and the Caribbean. Furthermore, publication bias in intervention research which tends to reject negative or neutral findings from publication may have confined the analysis and recommendations stemming from our systematic review (145). Another limitation pertains to the ways in which the studies measured quality, with numerous studies reporting “quality” data using proxy indicators such as number of services received (99). Similarly, many studies measured inputs, processes, and outputs, with only a few studies measuring outcomes and impact. Therefore, our intention of providing a holistic and comprehensive picture of QoC, beyond reductionist indicators, has been confined by the studies we were able to include in our inventory. Furthermore, our synthesis process involved subjectively coding findings into these six domains as at present studies do not have a streamlined process for measuring QoC.

## 5 Conclusions

This analysis contributes towards a need to streamline how QoC is understood and measured, to better define a dialogue around QoC that can be implemented across countries and people for equitable MNCH outcomes. One key takeaway from our study is the substantial need for further research to commit to addressing QoC in a streamlined way to help realize universal health coverage goals.

The findings in this systematic review have practical significance to both researchers and policymakers. The following recommendations are directed at researchers:

• Develop an updated guidelines with operationalized metrics for measuring QoC
• Encourage a concerted effort among researchers to adhere to these holistic and comprehensive indicators when testing QoC, rather than relying on reductionist outcome indicators
• Encourage researchers who have reported on aggregated data on QoC for MNCH on aggregated data on QoC for MNCH to conduct further analyses in which they disaggregate by public/private sector
• Encourage researchers to provide details on the type of health facilities in their samples (public sector, private sector, faith based, non-governmental, and so on)

The findings of this systematic review also have relevance for relevant policymakers, program implementers developing interventions for MNCH care, and global health practitioners more broadly. In summary, to ensure that MNCH coming from all types of facilities is of high quality and therefore ensuring that quality healthcare is evenly distributed, the following recommendations arise:

• In order to ensure effective care, making provider knowledge about components of care a priority through the dissemination of knowledge to both care-seekers and care providers. A four-week course designed to enhance QoC through clinics skills refresher course worked well in Pakistan (50), though this would need to be piloted in other contexts and disaggregated by facility type.
• To improve efficient care, lessons can be drawn from the SHOPS program (USAID's flagship initiative across sub-Saharan Africa) which led to improvements in the management of collecting blood samples and restructuring of outpatient department to reduce waiting times, through a coupon system which worked on a first come-first-serve basis (51). Vouchers reduced the proportion of women reporting service provision delays, therefore also contributing to better patient perception in terms of person-centered care.
• To improve equitable access to quality care for all SES status, accreditation and reimbursement programs can encourage poor women to access family planning and maternal and child health services at private birthing homes without financial constraints, as was the case in the Philippines (77). Importantly, such interventions that aim to address inequitable access between SES groups to quality MNCH care must be piloted in specific contexts to avoid unintended consequences, as in Uganda and India three maternal health social franchises who aimed to serve poorer groups ended up with most of their users concentrated in higher wealth quintiles (80). Notably, while equitable access to care is essential for desired health outcomes, this only performs if the care that is available to these women is of high quality itself.
• To provide better patient-centered care in terms of counselling and advice, training, guidelines, refresher courses and supervision is recommended for care providers.
• To improve safety in terms of skilled birth attendance, competency-based trainings could lead to improvement in the quality of ANC, delivery and post-natal care, which have found similar results in public-private partnership clinics, but not in public or private facilities in Pakistan (111). This links to the final point recommended to researchers above: to decipher why interventions lead to different outcomes in different types of facilities.
• To improve timeliness, vouchers to streamline services and reduce delays in services for people seeking care have been found in Bangladesh to be successful (70). As with all recommendations, this should be piloted in specific contexts, taking note of the outcomes in each type of facility and their determinants, before being scaled up.

## Data Availability

The original contributions presented in the study are included in the article/**Supplementary Material**, further inquiries can be directed to the corresponding author.
