# Supplementary material for: Private sector quality of care for maternal, new-born, and child health in low-and-middle-income countries: a secondary review
Source: Front Glob Womens Health. 2024 Apr 19;5:1369792. doi: 10.3389/fgwh.2024.1369792 (PMC11066217; doi:10.3389/fgwh.2024.1369792)
Supplement: Supplementary file 1 [file Table1.docx]

**Supplementary Annex 1: Summary table of included studies reporting outcome data on quality of care (n=110)**

| **Author, year [country]** | **Aim/objective(s)** | **Setting and population** | **Intervention description** | **Study design** | **Quality of care domains** | **Summary of findings on quality of care** | **Quality** |
| --- | --- | --- | --- | --- | --- | --- | --- |
| (Abt Associates 2015) [Malawi] | To report on four project goals on access to quality health care services | Nationwide private sector health facilities | To strengthen the enabling environment through capacity building, strengthening regulatory capacities, mapping private sector policies, and acting as broker between the ministry of health and private associations | Policy report | Efficient | Efficient: St John’s Hospital went from collecting blood samples through a small window to having a phlebotomy room. Likuni Mission Hospital used lean management activity to restructure its outpatient department and reduce patient waiting times – using a coupon to conduct a first come first served approach | Weak |
| (Abuya, Molynuex et al. 2004) [Kenya] | To describe the nature and practices of private health providers in rural Kenya, and user and health manager perspectives on quality of care offered | Private practitioners, private clinic users, and members of the district health management team in a rural area of Kenya | Delivery of quality care by the private sector | Mixed-methods | Timely, Equitable | Equitable: Clients reported that private clinics had better geographic access than government clinics  Timely: Clients reported that the main advantages offered by private clinics over government clinics are better geographic access, more reliable supplies of drugs and more rapid treatment | Weak |
| (Agha, Karim et al. 2003) [Nepal] | To examine the performance of a nurse and paramedic network, established to increase quality and utilization of reproductive healthcare services in Nepal | 64 private nurses and paramedics | Network members were provided 7 days of training on reproductive health, including family planning, A subset received additional intrauterine device (IUD) training. Intervention recipients received marketing and promotional training, referral linkages, and a quality monitor who visited monthly. | Quasi experimental | People-Centred | People-Centred: Clients at intervention or control clinics did not report higher satisfaction with client handling or with service charges. Client satisfaction with the physical look of the clinic increased from 26 to 64 percent at intervention clinics, while there was no significant change at control clinics | Weak |
| (Agha, Balal et al. 2004) [Uganda] | To assess the impact of a microfinance intervention with private sector midwives | Private midwives who were members of the Uganda Private Midwives Association for intervention (n=15) and control (n=7) | Loans provided to midwives on a revolving basis, with 15 midwives taking an average loan of $454, 11 repaying and taking second loans averaging $742.  Training of basic business skills was provided to loan recipients via the National Smallholder Business Center.  Follow up assessments of midwife performances were conducted | Quasi experimental | Equitable, Safe, People-Centred | Equitable: There was an increase in the proportion of people who visited the intervention clinics due to greater accessibility (0.44 to 0.53) - but not statistically significant. There were declines in the controls of people choosing comparison clinics for accessibility (0.57 to 0.43). But not significant  Safe: The proportion of those who attended comparison clinics due to cleanliness decreased from 0.25 to 0.05 – this perception of cleanliness metric was one of two statistically significant declines  People-Centred: There were increases in the proportion of clients who visited an intervention clinic due to the greater availability of drugs at that clinic (0.23 to 0.36), or fair charges (0.21 to 0.30), or greater privacy (0.03 to 0.10), or greater accessibility (0.44 to 0.53), or good physical outlook of the clinic (0.03 to 0.08), but these increases did not reach statistical significance. | Weak |
| (Agha 2009) [Uganda] | Examine the impact of a quality improvement package on the quality of reproductive health services provided by private midwives | 300 midwives across regional Ugandan Private Midwives Association branches | Intervention A consisted of midwives who received training in self-assessment tools and completed an action plan; Intervention B consisted of midwives who received the same but also had supervisors who received training in solving problems identified by the midwives. The control consisted of supervisors and midwives not trained in the quality improvement package | Quasi experimental | Timely, Effective, People-Centred | Effective: The average number of guidelines and job aids available at control group clinics was 4.7 at baseline and 5.4 at follow-up. The average number of guidelines and job aids available at Intervention A clinics was 3.9 at baseline and 4.5 at follow-up. The average number of guidelines and job aids available at Intervention B clinics was 4.1 at baseline and 3.9 at follow-up. The increase in the number of guidelines and job aids available at control group clinics was significantly greater than at Intervention B clinics (p=0.021). The increase in the number of guidelines and job aids available at Intervention A clinics was also significantly greater than at Intervention B clinics (p=0.027). Trends in the number of educational materials available at intervention and control group clinics did not differ significantly  Timely: The number of days per week services were provided at control group clinics was 6.8 at baseline and 6.7 at follow-up. Services were provided at Intervention A clinics 6.5 days per week at baseline and 6.6 days per week at follow- up. There was no significant difference in trends between Intervention A and control group clinics on the number of days per week services were provided. Services were provided at Intervention B clinics 6.2 days per week at baseline and 6.6 days per week at follow-up. The increase in the number of days services were provided was significantly greater at Intervention B clinics compared with control group clinics (p=0.005) and compared with Intervention A clinics (p=0.036)  People-Centred: Continuity of care scores were not significant different between intervention and control groups | Moderate |
| (Akhter and Schech 2018) [Bangladesh] | To understand why caesarean sections have become widespread among wealthier women in Dhaka | 30 women who had given birth in the preceding five years | Delivery of quality care by the private sector | Qualitative: in-depth interviews | People-Centred, | People-Centred: A perception across research participants is a preference for giving birth in private clinics or hospitals. Participants would not contemplate using a public hospital even if they could be treated by their preferred doctor: “As well, she was expecting a higher quality of care in a private hospital, and her privileged class position enabled her to make this choice. Being able to choose the place of childbirth and the medical staff is a way of displaying one’s high social class” | Weak |
| (Akwara, Alayon et al. 2003) [Uganda] | To measure changes in reproductive, maternal, and child health knowledge and behaviour in Delivery of Improved Services for Health (DISH) project districts | Health care facilities in 12 districts of Uganda | The study evaluates four primary interventions:  1. Yellow Star Program: partnered with Ministry of Health Quality Assurance Department and Health Promotion and Education Decisions to design 35 basic standards of high equality health services.  2. Adolescent-Friendly Reproductive Health Services: expansion of services to 34 health centres in 12 districts, including marketing and publicity services  3. Long-Term and Permanent Methods Marketing and Services: Staff training in seven hospitals to provide routine tubal ligation, vasectomy and Norplant services, as well as midwives and clinical officers to remove Norplant and counsel on other services.  4. Safe Motherhood Strategy: A radio and print campaign, as well as birth-planning cards, distributed to promote quality maternal care. A self-instructional manual for healthcare providers, community discussions by midwives, and community reasons persons to follow up people after antenatal care (ANC) clients. | Survey and facility observations | People-Centred | People-Centred: Providers at private facilities are much more likely than those at other facilities to encourage clients to discuss or ask questions about their treatment. Government and NGO facilities in first-phase districts are more likely than those in second-phase districts to encourage client discussion  Privacy remains an issue in many facilities, particularly NGO and private facilities, although only a slim majority of government facilities met this standard. Facilities in second-phase districts had clean waiting areas and provided private areas for physical examinations more often than those in first-phase districts. Government facilities are much more likely than others to have trained staff attending clients. Only 2 percent of NGO and PFP facilities met the training standard; One in five consultations in government facilities were rated acceptable compared with 15 percent in private and 7 percent in NGO facilities | Weak |
| (Allam, Oruganti et al. 2016) [India] | To evaluate HIV/AIDs services as part of the APAIDSON program, India | 115 patients accessing HIV testing and counselling and 115 people living with HIV in patient wards | Delivery of quality care by the private sector | Mixed-methods | Safe | Safe: Quality of HIV testing at the study sites had 100% concordance on EQAS | Weak* |
| (Anand and Sinha 2010) [India] | To establish the relationship between women’s utilization status for reproductive health services and to assess quality perceptions related to public or private health facilities | 6303 married women aged 15-39 in study regions | Delivery of quality care by the private sector | Regression analyses | Timely, Effective, People-Centred | Effective: Public facilities had an effectiveness score of 20.8% (no utilization), 12.2% (discontinuous utilization), 22.6% (initiation during follow up), 44.4% (continuous utilization)  Private facilities had an effectiveness score of 20.8% (no utilization), 12.2% (discontinuous utilization), 22.6% (initiation during follow up), 44.4% (continuous utilization)  Timely: Public facilities had a doctor availability score of 26.4% (no utilization), 12.3% (discontinuous utilization), 20.6% (initiation during follow up), 40.7% (continuous utilization)  Private facilities had a doctor availability score of 23.8% (no utilization), 12.3% (discontinuous utilization), 27.2% (initiation during follow up), 36.7% (continuous utilization)  Public facilities had a waiting time score of 25.8% (no utilization), 13.5% (discontinuous utilization), 24.5% (initiation during follow up), 36.2% (continuous utilization)  Private facilities had a waiting time score of 23.8% (no utilization), 12.3% (discontinuous utilization), 27.2% (initiation during follow up), 36.7% (continuous utilization)  People-Centred: Public facilities had a client treatment score of 24% (no utilization), 12.8% (discontinuous utilization), 23.8% (initiation during follow up), 39.5% (continuous utilization)  Private facilities had a client treatment score of 22.4% (no utilization), 12.2% (discontinuous utilization), 26.8% (initiation during follow up), 38.6% (continuous utilization)  Public facilities had a privacy score of 28.7% (no utilization), 11.9% (discontinuous utilization), 25.5% (initiation during follow up), 33.9% (continuous utilization)  Private facilities had a privacy score of 20.7% (no utilization), 12.5% (discontinuous utilization), 25.9% (initiation during follow up), 40.9% (continuous utilization) | Weak |
| (Angeles, Hutchinson et al. 2003) [Bangladesh] | To collect information on and monitor changes in the United States Agency for International Development (USAID) performance indicators since the Rural Service Delivery Partnership (RSDP) Baseline Survey and to evaluate the impact of the RSDP programme on the health of the project population | 9768 women ever married aged 10 to 49 years in RSDP program areas and 3,176 from comparison, non-programme areas | Delivery of quality care by the private sector | Cohort analytic study | People-Centred, Timely, Equitable | Equitable: The large proportion of antenatal care provided by RSDP may have helped to close the gap in antenatal care use between the rich and poor. This gap was approximately 10 percentage points smaller in RSDP areas – where 34.6 percent of women in the lowest socioeconomic quintile used antenatal care compared to 70.5 percent of women in the richest socioeconomic quintile – than in non-RSDP areas. In non-RSDP areas, rich women were more than three times as likely to use antenatal care as women in the poorest socioeconomic quintile.  Timely: The mean waiting time at RSDP clinics was 18.5 minutes, 44.5 percent of users did not have to wait for service; The mean waiting time was also slightly higher in government clinics compared to RSDP clinics in both project and non- project areas  People-Centred: The majority of users rated staff behaviour (99.3 percent) and quality of services (98.7 percent) as good or very good and almost all of them (99.4 percent) would recommend the hospital/clinics to others. Comparable levels of satisfaction with the quality of service and staff behaviour at the RSDP clinics were observed in non-RSDP areas. | Moderate |
| (Angeles, Lance et al. 2005) [Bangladesh] | To collect information about knowledge, awareness, and the use of services related to family planning and maternal and child health through the non-governmental organization (NGO) Service Delivery Program and alternatives | 5,691 women ever married aged 10 to 49 years in NGO Service Delivery Program areas and 4,201 from comparison, non-programme areas | Delivery of quality care by the private sector | Cohort analytic study | People-Centred, Timely, Equitable | Equitable: In NSDP areas, the mean travel time to NDSP satellite clinics was 7.2 minutes. The average travel time to an NDSP clinic was 13.1 minutes. Users of government hospitals/clinics also recalled longer travel  Timely: In NSDP areas, the mean travel time to NDSP satellite clinics was 7.2 minutes. The mean waiting time was 14.3 minutes; In NSDP areas, the majority of users of NSDP clinics (95.1%) reported that staff spent enough time for them during their last visit, 90.3% felt that they talked to them nicely, and 97.6% reported that they provided enough attention to their needs. The average travel time to an NDSP clinic was 13.1 minutes, while the average waiting time was 20.8 minutes; Users of government hospitals/clinics also recalled longer travel and waiting times. The average waiting time was substantially lower at private hospital/ clinics  People-Centred: 93.7% users of NSDP satellite clinics in NSDP areas reported that clinic staff spent enough time with them. About 85.7% felt that staff talked to them nicely, and 95.8% indicated that staff devoted enough attention to their needs. | Weak |
| (Angeles, Lance et al. 2006) [Bangladesh] | To measure changes in the USAID performance indicators for the NGO Service Delivery Program since mid-project evaluations in 2003. | 7,652 women ever married aged 10 to 49 years in NGO Service Delivery Program areas and 4,418 from comparison, non-programme areas | Delivery of quality care by the private sector | Cohort analytic study | People-Centred, Timely | Timely: The majority of users of NSDP static and satellite clinics said staff spent sufficient time with them. Travel times and waiting times were slightly longer for NSDP clinics in NSDP areas (relative to government clinics in non-project areas)  People-Centred: Quality of care at NSDP facilities remained high. As in 2001 and 2003, almost all users of NSDP static and satellite clinics said that staff were nice, spent a sufficient amount of time with them, and gave enough attention to their needs.  More than nine in 10 NSDP satellite clinic users in NSDP areas and government satellite clinic users in non-project areas said that staff talked to them nicely and paid sufficient attention to their needs. | Moderate |
| (Annigeri, Prosser et al. 2004) [India] | To provide suggestions for future public-private partnerships for USAID in India. | 192 urban health centres in 74 municipalities in Andhra Pradesh | The Commissioner of Family Welfare built 192 urban health centres. Intervention aimed at providing basic reproductive and child health preventive care through: service delivery, community mobilisation, and behaviour change communication. Implemented via the local urban health centre and community outreach. | Qualitative interviews and field visits | Equitable | Equitable: Found that the urban health centres primarily provided care for poorer people. In addition, found that some people who can afford to pay sought to obtain free services. This varied, as some urban health centres have eligibility criteria and others do not | Weak |
| (Anwar, Begum et al. 2016) [Bangladesh] | To explore the structural and outcome dimension of quality of services | 1343 case records from 34 facilities | Delivery of quality care by the private sector | Cross-sectional | People-Centred | People-Centred: The availability of process indicators as a proof to patient centred care were varied, bed side screen (55%), comment box (32%), BCC materials (15%), receptionist (100%) and displayed service price (15%) | Weak |
| (Arrieta, García-Prado et al. 2011) [Bolivia, Colombia, Dominican Republic, Guatemala, Nicaragua, Peru] | To compare the effective of private and public sectors in providing prenatal care | Demographic and Health Surveys across six countries | Delivery of quality care by the private sector | Regression analyses | Effective | Effective: The higher number of prenatal visits in the private sector, which is clearly above the prescribed number of visits recommended by the WHO and above the number of visits provided by public facilities, may not necessarily indicate over-utilization. If the excessive number of visits in the private sector is associated with higher quality, then the additional number of visits can be justified, and they could even be cost-effective. However, if outcomes do not improve with more prenatal visits, then those additional visits are unnecessary, implying overutilization.  Over-utilization in the private sector is not accompanied by better birth weights | Strong |
| (Baig and Shahid 2017) [Pakistan] | To explore whether pre-service, in-service, experience and supervision improve service quality and ultimately delivery | 112 community midwife (CMW) led clinics, 93 DOH facilities, 109 private facilities and 327 public private partnership led facilities | Training: Competency based trainings were used to enhance skills and performance of healthcare workers. The training approach included group based training of service providers, class room teachings, hands-on practice on mannequins and real clients in clinical settings. Standardized learning resource packages were developed based on evidence based national and international guidelines.  Quality Improvement and Patient Safety (QIPS): Jhpiego’s Standards- Based Management and Recognition– performance and quality improvement approach was adapted to produce a self-assessment checklist. The assessment was conducted bi-yearly followed by data-driven action plan. | Controlled pre-and-post analysis | Safe | Safe: Preliminary analysis indicated that significant improvement in quality of antenatal, labour and delivery and postnatal care was observed in CMW centres that received trainings (p-value<0.001) as compared to those CMW centres that did not receive trainings. Similar results were observed among PPP led facilities. Ironically, no difference in quality score was observed in public and private facilities | Weak |
| (Bakibinga, Ziraba et al. 2016) [Kenya] | To examine the perspectives of women and community health volunteers (CHVs) regarding the use and quality of maternal and child health services in public and private facilities | 849 women and girls aged 12-49  936 children [via caregivers] from Korogocho and Viwandani informal settlements, Nairobi | The intervention was entitled ‘Partnership on Maternal, Newborn and Child Health’, and included: infrastructural upgrade of selected Private Not-For-Profit (PNFP) health facilities operating in two informal settlements; building capacity for both health care providers and the sub–County Health Management Teams in Ruaraka, Kasarani and Makadara sub-counties of Nairobi, where Korogocho and Viwandani, respectively, are located; and facilitating provision of supportive supervision by the local district health authorities that are forming networks of CHVs to create demand for the health services. | Mixed-methods | Timely, People-Centred | Timely: Respondents reported that private facilities provided services faster (because of fewer clients). They also had a preference for private because these facilities were open for longer than public facilities (e.g., at night)  People-Centred: Attitudes of clinicians was referenced as a factor in positive quality of services. Respondents reported that private facilities were more courteous than public facilities. | Moderate |
| (Baliga, Ravikiran et al. 2016) [India] | To understand perceived quality of care among parents and children admitted to two medical college hospitals – one run with private partnership models and one operated by the government | Parents of patients in a public private partnership hospital (n=580) and a public health hospital (n=461) in southern India | Delivery of quality care by the private sector | Regression analysis | People-Centred, Timely | People-Centred and Timely: In PPP model hospital duration of time spent waiting for treatment and manner of other support staff were significant predictors for overall satisfaction while other domains were less influencing on parental satisfaction  Among the category of responders who had overall satisfaction (satisfaction rating >8 in the 1-10 scale) from PPP model, perceived the care as good (rating >4 in the 1-5 scale) in most domains compared to their counterparts from PH model | Weak |
| (Banerjee, Andersen et al. 2015) [India] | To describe the implementation of the Yukti Yojana programme to expand availability of safe abortion services in Bihar, India | 48 in-depth interviews of providers and key stakeholders and 16 facility evaluations | The programme was advertised through media and aimed to accredit private sector health facilities to provide abortion-related services free of charge.  A technical advisory group monitored quality of the programme, including an NGO contracted to monitory quality of services, and an organisation to provide independent data collection. Facilities that were given accreditation by a committee were contracted and reimbursed for provided abortion services. | Descriptive analysis | People-Centred | People-Centred: The client satisfaction index showed that slightly less than half the women coming for abortion services (46 %) expressed very high levels of satisfaction compared to 44 and 10 % who expressed moderate or low level of satisfaction, respectively. | Moderate |
| (Bangladesh. National Institute of Population, Training et al. 2016) [Bangladesh] | To report the results of the 2014 Health Facility Survey | 1549 health facilities | Delivery of quality care by the private sector | Quantitative findings from survey data | Safe | Safe: District and upazila public facilities (46 percent) are more likely than NGO facilities (29 percent) and private hospitals (21 percent) to report QA activities and have supporting documentation. Among the district and upazila facilities, 63 percent of DHs, 47 percent of UHCs, and 32 percent of MCWCs reported QA activities and provided documentation. In contrast, only 6 percent of union level facilities and 8 percent of CCs have documented QA activities | Moderate |
| (Barber, Bertozzi et al. 2007) [Mexico] | To evaluate prenatal care quality in rural Mexican communities | 3533 women who reported they received prenatal services between 1997-2003 | Delivery of quality care by the private sector | Regression analysis | People-Centred | People-Centred: The percentage of prenatal care procedures received by clinical settings relating to people-centred care were advice on lactation – 92.24% in social security facilities, 92.93% in IMSS Oportunidades facilities, 91.30% in government facilities, 71.04% in private facilities.  Those advised on family planning methods were 90.17% in social security facilities, 91.85% in IMSS Oportunidades facilities, 85.72% in government facilities, 55.21% in private facilities. | Strong |
| (Barber, Gertler et al. 2007) [Indonesia] | Examine quality variations by clinic setting, geographic region, and household wealth in Indonesia | Household survey (n=7629) | Delivery of quality care by the private sector | Regression analyses | Equitable | Equitable: On average, Outer Java-Bali was associated with significantly lower quality of all types of care (p ≤ 0.01). Private physicians and public health centers offered above-average child and adult curative care. Public health centers offered the highest- quality prenatal care. By clinical setting, private physicians offered above-average care for all scenarios in both regions, with the exception of prenatal care in Outer Java-Bali. Private nurses offered below-average care for all scenarios and regions. Public facilities offered above-average care in Java-Bali and below-average care in Outer Java-Bali.  There were significant wealth differences in quality available, with the poorest households having access to higher-quality prenatal care. Based on the facility reports, the poor had access to health care providers with quality scores 5.9 percentage points higher than those of providers available to the wealthiest. Based on the maternal reports, however, the poor actually received a lower level of quality. The poor received 3.5 percentage points fewer procedures compared with the wealthiest. | Strong |
| (Barber 2006) [Mexico] | To evaluate whether prenatal care quality varies by public and private clinic settings and by household wealth | Household survey (n=16125) | Delivery of quality care by the private sector | Regression analyses | Equitable | Equitable: The poorest sample quartile received significantly fewer procedures compared with the wealthiest on average and in private clinical settings (P ≤ 0.05). Linear trend tests performed to test wealth discrepancies indicate that an increase by one wealth quartile (measuring from poorest to wealthiest in the sample) is associated with a small but significant increase in procedures received on aver- age [1.3%, 95% confidence interval (CI) = 0.0–2.6; P = 0.05]. However, within private clinical settings, an increase by one wealth quartile is associated with a 5% increase in procedures received (4.7%, 95% CI = 2.42–7.1; P < 0.01). | Strong |
| (Bell, Zimmerman et al. 2018) [Nepal] | To examine abortion service provision in Nepal | Nepal Health Facility Survey 2015 | Delivery of quality care by the private sector | Descriptive statistics | Timely | Timely: Service readiness relates to MVA, MA and PAC. Public facilities more likely to have all required components, no private facility met these criteria. Public facility readiness was low, no private facilities had all service readiness components for any abortion service. | Weak |
| (Benova, Dennis et al. 2018) [Uganda] | To aid the formulation of future strategies by describing the historical development and recent levels of utilization of maternal care in Uganda, focusing on ANC and delivery care | Ugandan Demographic and Health Survey | Delivery of quality care by the private sector | Cross-sectional analyses | Effective | Effective: Coverage differed significantly between the public and the private sectors for two components: having had blood pressure and urine sample taken (both were higher among users of private providers). Overall, women who received recommended ANC reported receiving on average 4.8 of the eight components; 4.9 among public and 4.2 among private sector users (not statistically different p > 0.05). However, 9.6% of women with recommended ANC reported receiving all eight ANC care components; this proportion was significantly higher (p = 0.002) for women using the private sector (17.5%) compared to those using public sector providers (8.5%)  Overall, women reported receiving on average 2.5 of the four components (2.5 in public and 2.3 in private sector; not statistically different); with 13.0% of women delivering in health facilities receiving all four components (12.6% in public and 14.2% in private sector, difference not statistically significant) | Weak |
| (Benova, Macleod et al. 2015) [57 countries] | To examine the role of private-sector providers in the provision of appropriate delivery-care services among women who had a birth in the recall period | Demographic and Health Survey data | Delivery of quality care by the private sector | Descriptive statistics | Equitable | Equitable: In the public sector, the percentage point difference in having a SBA was 3 between the poorest and richest wealth quintiles (95% in poorest and 98% in richest) compared to a 2 percentage point difference in the private sector (97% in poorest and 99% in richest). The proportion of births to women in the poorest quintile attended by a doctor was higher in the private sector than in the public sector (63% and 45%, respectively) | Weak |
| (Bjorkman-Nykvist, Guariso et al. 2015) [Uganda] | To asses the impact of non-profit entrepreneurial models of community health delivery | 8120 households in 214 villages across 10 districts | A cluster-randomized controlled trial from 214 rural villages in 10 districts in Uganda. In treatment villages, Living Goods and BRAC Community Health Promoters conducting home visits, educating households on essential health behaviours and selling preventive and curative health products at 20-30% below prevailing retail prices were deployed over a three-year period (2011- 2013) | Randomized controlled trial | People-Centred, Safe, Effective | Effective and Safe: The Community Health Promoter program could affect mortality through a number of channels, including improved access to treatment and health services, improved quality of treatment and health services, better access and knowledge of prevention, and by influencing other actors to improve the quality of services and products that they provide/sell. They find evidence supporting all these channels, including a 17% increase in treatment of diarrhoea with ORS and zinc; an 54% increase, albeit starting from low levels, in follow-up visits for under-five children falling sick with malaria, ARI or diarrhoea and an 72% increase, again starting from a low level, in home visits in the first seven postnatal days. While the likelihood of treatment with ACTs and antibiotics are similar across assignment arms, households in treatment villages are significantly more likely to purchase ACTs, antibiotics, and ORS/zinc from CHPs  Timely: Evidence supporting the intervention, including a 17% increase in treatment of diarrhoea with ORS and zinc; an 54% increase, albeit starting from low levels, in follow-up visits for under-five children falling sick with malaria, ARI or diarrhoea and an 72% increase, again starting from a low level, in home visits in the first seven postnatal days  People-Centred: The Community Health Promoter programme impacted mortality by influencing other actors to improve the quality of services and products that they provide / sell. | Weak |
| (Bojalil, Guiscafré et al. 1998) [Mexico] | To evaluate the clinical management of acute diarrhoea and ARI in children under five in primary health care | Children under five at 62 private and 43 public GPs | Delivery of quality care by the private sector | Descriptive statistics | Safe | Safe: Half private and 7% of public GPs gave wrong rehydration scheme. Sixty-three per cent of private GPs gave incorrect dietary advice, compared to 13% of public GPs; sixty-six per cent of private GPs and 27% of public GPs made a wrong antimicrobial prescription.  In 49% of cases the prescription given by private GPs was not justified, compared to 3% prescribed by public GPs; 66% of the private and 30% of the public sector practitioners made an incorrect prescription of a drug.  Symptomatic drugs were incorrectly prescribed in 58% of the cases by private practitioners and in 20% of those treated by public sector practitioners.  Only 15% of private GPs managed correctly three of four of the evaluated aspects of diarrhoea, compared to 63% of public GPs (p<0.001). In ARI cases, 29% of private and 73% of public GPs managed correctly two of three of the evaluated aspects | Moderate |
| (Bojalil, Kirkwood et al. 2007) [Mexico] | To provide information to better implement interventions linked with the Integrated management of Childhood Illnesses programme | 75 mothers whose child (up to age 5) had died within the last 90 days | Delivery of quality care by the private sector | Analysis of "Death narratives" and competency evaluations | Safe | Safe: 52.9% of doctors were implicated in contributing to a child death with private doctors more likely to be implicated than public doctors. Overall, private doctors accounted for 1.4 times as many consultations as public doctors, but were implicated in 1.8 times the number of deaths | Weak |
| (Boller, Wyss et al. 2003) [Tanzania] | To compare the quality of antenatal care offered by public and private providers | 166 women attending public health facilities  188 women attending private health facilities | Delivery of quality care by the private sector | Regression analysis | Structural, People-Centred | People-Centred: Maintenance was generally better in private facilities. Regarding basic diagnostic tools, equipment was clearly better in the private sector. 29% of public facilities had toilets with water to flush compared to 35% of private. 43% of public facilities had waiting places for all the women compared to 78% of private. 100% of both clinics had a private examination room. 29% of public facilities had water to wash hands compared to 56% of private.  Structural: The median overall score for structural attributes of quality, out of a maximum of 72, was 51 (range 35–54) for the public and 64 (range 56–72) for the private sector. These differences were significant | Weak |
| (Carter, Ndhlovu et al. 2016) [Zambia] | To assess the feasibility of collecting geographically and temporally concurrent household and health care provider data | 355 rural and 469 urban households with at least one eligible mother of a child under 5 in Choma District, Southern Province, Zambia | Delivery of quality care by the private sector | Descriptive statistics and facility evaluations | Structural, Effective | Effective: Facilities performed the poorest on domains related to training and guidelines and knowledge. Community based agents (CBAs) performed poorly on availability of basic medicines and management capacity. Pharmacies excelled on measures of medicine availability but failed on all other measures. Shops and traditional practitioners performed poorly on all domains  Structural: Structural quality scores varied most by category of provider, and in most cases did not vary greatly within provider categories used in the aggregate linking. While there were a few providers whose scores were notably above or below others within their category, these were provider categories that did not align with common source of care categories | Strong |
| (Chakraborty and Frick 2002) [India] | To evaluate the technical quality of care for acute respiratory infections (ARI) | Mothers of children under 5 and providers of care at 40 facilities | Delivery of quality care by the private sector | Quality assessment | Safe, Effective | Effective: In comparison with WHO standards, rural private providers included in this study have inadequate ARI disease management practices. For example, the average score obtained by the 40 providers on ARI disease management was only 8.00 out of an expected score of 33. This indicates a deficit of 76% between expected standards and observed practices. Provider performance was particularly poor for critical ARI disease management practices such as checking the respiratory rate and chest in drawing  Safe: In comparison with WHO standards, rural private providers included in this study have inadequate ARI disease management practices. For example, the average score obtained by the 40 providers on ARI disease management was only 8.00 out of an expected score of 33. This indicates a deficit of 76% between expected standards and observed practices. Provider performance was particularly poor for critical ARI disease manage- ment practices such as checking the respiratory rate and chest in drawing. According to the WHO, checking the respiratory rate and chest in drawing are sensitive indicators for diagnosing pneumonia (WHO, 1991). However, among this group of providers, only 14% measured the respiratory rate and 9% checked chest in drawing. Private providers ARI disease management as “inadequate” | Weak |
| (Chakraborty, D'Souza et al. 2000) [India] | To improve the technical quality of care among private health practitioners, to test implementation feasibility and effectiveness of the intervention methods | Mothers of children under 5 | Meetings with providers, training using WHO case management guidelines | Cross-sectional survey pre and post intervention | Effective, Safe, People-Centred | Effective: The data show that before the interventions, private providers’ actions on several important case management practices for childhood illnesses were inadequate. For example, according to the WHO IMCI guidelines, checking the respiration rate in children with a cough or difficulty breathing is critical for accurately diagnosing pneumonia.  For the management of dehydration during diarrhoea, the WHO recommends the use of ORS or home-based sugar and salt solutions (WHO 1995). At baseline, private providers included in this study recommended ORS or sugar–salt solution in only 16% of cases. After the interventions, private providers’ performance on this case management guideline had risen to 48%, a 22% change.  Safe: To summarize, after participation in the VCR and INFEC- TOM, private providers’ performance on the WHO recommended case management practices for childhood illnesses was better than at baseline, with many of the individual differences achieving statistical significance. Comparison of mean overall case management scores by bivariate analysis also indicated a statistically significant difference between provider performance on non-disease-specific case management practices before and after the intervention (t = 14.29, p < 0.05).  People-Centred: Private providers participating in all the quality improvement interventions were compared with those who had only participated in the contracting visits and ongoing monitoring. In Dumka district, participation in the group information sessions added only marginally to improvements in practices, with providers participating in both interventions performing better than their counterparts in only 2 of 22 case management practices. In Bettiah, those who participated in both interventions performed better than their counterparts on only 5 of 22 case management practices.  Provider participation in public health activities at the community level, such as health education, was positively influenced by the intervention. Since the interventions were initiated, providers have begun to participate in women’s organization (mahila mandal) meetings. Topics discussed by private providers in these meetings included water and sanitation (21%) and use of oral rehydration or sugar–salt solution during diarrhoea (24%). | Weak |
| (Chemonics 2015) [Philippines] | To increase and sustain private sector provision of quality family planning and maternal health services, to increase utilization of quality family planning and maternal and child health services, and to improve the policy environment for private sector provision of services and products | Birthing homes, hospitals, provincial program managers | Engaged with PhilHealth and the Department of Health at the national and regional levels to provide technical assistance to provincial program managers, facilitate accreditation and reimbursement processes, and roll out a quality assurance packages and training documents. | Descriptive statistics of process indicators | Equitable | Equitable: Increased access to maternal health services for indigent women. The accreditation of 505 PPMs and 307 birthing homes makes professional delivery services accessible to more women. With the government’s Universal Health Care Program, more women are enrolled in the national health insurance scheme as PhilHealth members. These women are now able to access FP‐MCH services at private birthing homes without the financial constraints that previously burdened them, with PPMs implementing the no‐balance billing policy—seeking reimbursement from PhilHealth for services rendered rather than charging the client | Weak* |
| (Chen, Dai et al. 2013) [China] | To evaluate the ANC quality and compare the quality of care between public and private sectors | 1079 caregivers of children under 5 | Delivery of quality care by the private sector | Cross-sectional | Effective | Effective: More women in public than in private sector had a maternal health care booklet (74% vs 47%, P < 0.001)  Significantly fewer women in private than in public sector were weighed, underwent haemoglobin, urine, syphilis, HBV, and HIV/AIDS test, were given iron supplements or advice on syphilis, HBV, and HIV/AIDS.  When combining ANC procedures together, only 7 (1%) women reported to have received all 16 services from the public sector. No women reported to have received all 16 services from township level hospitals or the private sector | Strong |
| (Coulibaly, DeLisle et al. 2002) [Côte D’Ivore] | To evaluate mothers’ perceptions of quality growth and monitoring and promotion | 200 mothers | Delivery of quality care by the private sector | In-depth interviews and focus groups | Safe, People-Centred, Equitable | Equitable: Mothers rated the overall program as such, and the specific components separately. The proportion of respondents who declined to rate the program was twice as high in the rural than urban programs (25% vs. 12.5%, respectively). Mean scores of overall satisfaction ranged from 2.75 ± 0.3 (government-run urban program) to 3.25 ± 0.5 (mission program) on a scale of 4. The rural government program rated particularly low on technical procedures. Mothers complained that this program consisted merely of child weighing, without any complementary activities or any feedback on the weight, health, or nutritional status of the child. The civil NGO obtained a lower mark than the mission-run program, particularly for its organization.  Safe: There were more complaints on technical procedures in government-run programs than in NGO programs, particularly about the lack of advice given to them, the absence of investigation into the causes of growth failure, that immunization status was not checked, and no appointment was given for the next GMP  People-Centred: There were more complaints on technical procedures in government-run programs than in NGO programs, particularly about the lack of advice given to them, the absence of investigation into the causes of growth failure, that immunization status was not checked, and no appointment was given for the next GMP. In the NGO programs, nutritional advice was usually given in the course of group education, including the promotion of breastfeeding and the intro- duction and preparation of complementary foods. Mothers valued the advice, although they objected to the high price and amount of work involved in preparing some of the complementary foods advocated, for instance, cereal pap mixed with soybean flour and milk. In the NGO programs mothers were asked about the health of the child, but it was only at the mission where a history of the child’s diet was systematically taken, where children were examined once in a while even if they were apparently healthy, and where mothers were informed of the weight of their children. Mothers were generally unaware of the growth chart. Only in rare instances would the growth curve of a malnourished child be shown to the mother in order for her to see the weight deficit | Weak |
| (Danel and Forgia 2005) [Guatemala] | To assess the performance of Guatemala’s programme to extend coverage of basic health services and to determine the relative economic efficiency of different service delivery models | National with a target population of children under 5 and prenatal care seekers. | Delivery of quality care by the private sector | Regression analysis | People-Centred, Effective, Timely, Efficient | Effective: The quality of PNC received, as measured by tetanus toxoid vaccination and iron and folate supplementation, was also highest in mixed provider catchment areas. Rates for these indicators were higher in traditional catchment areas compared to direct provider areas.  Efficient: Economic efficiency: " Adjusting for effective coverage,79 PSSs [(proveedora de servicios de salud [health ser- vices provider])] become the least-cost provider (Q5.7 per capita), followed by HPs [health posts] (Q7.6) and ADMSSs (Q8.5). The differences among the pairs of providers are statistically significant (P<.05). Nevertheless, because of the lower effective population coverage by HPs, they provide an average of 4.0 interventions per capita, compared to 2.2 for the ADMSSs [(administradora de servicios de salud [ADMSSs])] and 2.0 for the PSSs. In short, for the population that the HPs effectively cover, they provide more services per capita than the other provider types  Equitable: Immunization rates varied considerably among the four departments, with Totonicapán having significantly worse rates  Immunization rates for all four departments together are shown for catchment areas for direct and traditional providers because they both included Totonicapán. For the departments of Quiché and Alta Verapaz, children in mixed provider catchment areas consistently have the best immunization rates. Children who live in direct provider catchment areas in Quiché and Alta Verapaz consistently have the lowest immunization rates. However, direct provider and traditional areas have very similar rates when data from all four departments are used.  Timely: More than 70 percent of women responded positively to these questions for all three types of providers. The exceptions were answers to questions about traditional providers’ waiting times and friendliness of care for children with respiratory illnesses  People-Centred: Satisfaction with care was measured using four indicators (problem resolution; provision of medicines; waiting more than one hour; and friendliness of services). More than 70 percent of women responded positively to these questions for all three types of providers. The exceptions were answers to questions about traditional providers’ waiting times and friendliness of care for children with respiratory illnesses | Weak |
| (de Azevedo Bittencourt, Queiroz Gurgel et al. 2015) [Brazil] | To analyse maternity facilities in Brazil and explore the relationship between structural adequacy of facilities | 266 hospital visits, 90 mothers interviewed | Delivery of quality care by the private sector | Regression analyses | Timely, Structural Quality | Timely: Public facilities without an NICU had poor availability of 24 hour on-call paediatricians, private sector performed worst for the availability of medicine although had better 24 hour on call | Moderate |
| (Dennis, Benova et al. 2019) [Kenya] | To understand the determinants of how early a person initiatives ANC during a pregnancy, what the factors influencing delivery locations are, and what determines whether all three recommended ANC and PNC services are received | Women aged 15-49 who had reported at least one live birth in 5 years preceding the survey | An accreditation programme using reproductive health vouchers from 2006 and 2016, in which poor women could purchase subsidized vouchers that covered the cost of four ANC visits, facility delivery, and PNC. | Case control | People-Centred | People-Centred: The proportion of births that received continuous care and completed the maternal health continuum pathway as recommended increased from 31.7% to 56.1% in voucher counties and 30.6% to 46.8% in comparison counties. In both study groups, the use of continuous, suboptimal care remained fairly constant over time, ranging from 16.3% in voucher counties in Period 1 to 22.9% in comparison counties in Period 3  Overall, the adjusted odds of continuous care use were 1.50 times higher in voucher counties than in comparison counties | Weak |
| (Diamond-Smith, Sudhinaraset et al. 2016) [Kenya and Namibia] | To describe the clinic quality of facilities in Kenya and Namibia, the perceived quality of patients exiting facilities, and determine if perceived quality is predictive of clinical quality | National Service Provision Assessment data linked to the Demographic and Health Survey | Delivery of quality care by the private sector | Regression analysis | Equitable | Equitable: Overall, there were no differences between private and public facilities in either country for Routine care or ANC, and private facilities performed better in Kenya on EmOC and EmNC (no significant differences in Namibia). There are many possible explanations for the differences in private compared to public facilities, including more resources (perhaps due to patient fees or charitable funding sources), better trained or incentivized staff, higher quality standards or expectations, etc.  However, it is important to note that despite people’s perceptions of quality being better at private facilities, there was no significant association between type of facility (private/public) and the ANC quality score. | Moderate |
| (Do and Agha 2009) [Uganda] | To evaluate the quality of three types of services – antenatal care, family planning, and postnatal care | Private midwives across Kampala and three regions (Central, Eastern and Western) were put into three intervention groups: A (n=85), B (n=89), and a comparison group (n=74) | A quality improvement packaged included: a form to review service statistics, a provider self-assessment tool, a linked action plan, and a tool to enable supervisor solutions. Intervention Group A were midwives who received one-day training on how to use the tool, supervisors were not trained. Intervention Group B consisted of midwives and supervisor training. | Pre-post-test quasi-experimental | Safe, People-Centred | Safe and People-Centred: Between baseline and follow-up, changes in quality of care did not occur in all three groups: only group B achieved significant improvements in both counselling and technical quality. The comparison group and group A had little quality improvement  People-Centred: There were no significant differences in continuity of care scores between intervention and control group clinics | Weak |
| (Do 2009) [Uganda] | To examine the agreement between providers’ self-assessment of the quality of services and the actual quality of services observed; to assess whether the use of the self-assessment tool improved this agreement | 276 midwives at baseline and 248 at follow-up | In intervention group A, midwives received a one-day training in using the self- assessment tool and completing an action plan, but their supervisors were not given training in problem solving and mobilizing external resources to assist midwives in problem solving. In intervention group B, midwives received the same training in the self-assessment tool and action plan, and their supervisors were trained in problem solving and resource mobilization. Midwives in groups A and B used the self- assessment tool monthly to assess a variety of service aspects, and then met with their supervisors to work on solutions to problems identified | Pre-post-test quasi-experimental | Safe, Effective, Observed Quality | Effective: With regard to subjective assessment of the overall quality of services, there was a general agreement at both baseline and follow-up between observed quality scores and the subjective assessment: i.e., observed quality scores were higher among providers who said their services were good. With the exception of FP technical score and counseling score, all of the differences in technical scores between the good and poor/fair overall subjective quality groups were statistically significant  Safe: Significant changes were observed for ANC technical competence: more midwives reported that improvements were needed at follow-up compared to baseline, especially among the comparison group (50% versus 26%, p<.01). | Weak |
| (Eichler, Auxila et al. 2009) [Haiti] | To assess the outcomes of scaling up a performance based payment programme among NGOs | NGO healthcare providers | Piloting of a performance-based payment scheme - The NGOs agreed on a new contract that would pay 95 percent of the budget established under the existing expenditure-based reimbursement contract and including the possibility of a bonus of as much as 10 percent of the budget. NGOs were thus assuming the risk of losing 5 percent of the agreed budget if they did not reach targets, but they stood to gain an additional 5 percent if they did | Descriptive | Effective, Safe | Effective: Results suggest that being paid based on performance is associated with a 13 to 24 percentage point increase in immunization coverage and a 17 to 27 percentage point increase in attended deliveries.  Safe: Results suggest that being paid based on performance is associated with a 13 to 24 percentage point increase in immunization coverage and a 17 to 27 percentage point increase in attended deliveries | Weak |
| (Epiu, Wabule et al. 2017) [Uganda] | To estimate the proportion of high level hospitals that meet the WFSA international standards | 64 healthcare providers that offer obstetric anaesthesia | Delivery of quality care by the private sector | Descriptive cross-sectional | Safe | Safe: Only 3 facilities fulfilled the WFSA International standards for safe anaesthesia. 61 hospitals did not. | Moderate |
| (Farahbakhsh, Sadeghi-Bazargani et al. 2012) [Iran] | To conduct a comparative observation study of health service delivery programmes over the period 2001-2002 | 1000 households, 20 clients per health facility, all physicians and professional staff in the health facilities | Delivery of quality care by the private sector | Comparative observational study with regression analyses | People-Centred, Timely | Timely: There was no significant difference between cooperative health centres and public health centres in child care follow up  People-Centred: Mothers' knowledge about the health care status of their children was 77.6% in cooperative health centres and 80.6% in PHCs. Women covered by CHCs had higher knowledge about family planning methods. There was significant difference was found between the knowledge level of women covered by CHCs about the importance of cervical examination when compared with PHCs (59 and 52 respectively) | Weak |
| (Field, Abo et al. 2018) [Papua New Guinea] | To describe the Health Program model and report the changes In service delivery in the first 2 years of implementation | 20 open health centres and 38 health workers | The intervention had three components: the first bought stakeholders together to coordinate service delivery, the second supported the provision of medical equipment and structural needs, and the third focused on delivery primary healthcare at the community level. | Mixed methods | Timely, Safe, Effective | Effective: Training for health workers included officer-in-charge training, essential obstetric care (EOC) training, basic management training for rural health workforce, provider-initiated counselling and testing for HIV. The training that health workers cited most commonly as resulting in changed practices was the EOC training  Safe: In contrast to the quantitative data indicating a decline in supervised deliveries coverage in the Health Program area, several health workers cited that there were now more supervised deliveries as a result of the EOC training. Supervised deliveries were also reported to increase after renovations to the health facility as reported by one health worker: “So the changes like building this new labour ward. So we have seen the improvements nowadays. All the ladies coming in and delivering in these ward facilities”  However, a lack of a dedicated space within other health facilities for deliveries was noted by some health workers as a barrier to supervised deliveries in the health facility: “At the moment we don’t have facilities for the deliveries that we can do in the facility. We go out to the bush or to their homes and help women deliver”  Timely: The training that health workers cited most commonly as resulting in changed practices was the EOC training. For example, one health worker changed the availability of antenatal care services: “Before we had certain days to attend to antenatal matters, but when I went for the EOC [essential obstetric care] course I was taught to attend to the mother when she comes at any time ... We had certain days [for antenatal care] but now they come in whichever time we give them for their next date of visit” | Weak |
| (Fischer, Musau et al. 2004) [Kenya] | To understand the mechanisms for increasing the sustainability of comprehensive PAC services | 200 PAC providers | The PRIME-assisted program for private nurse-midwives emphasises a comprehensive approach to primary-level PAC services in an effort to reduce maternal mortality or morbidity and decrease the chances of repeat abortion among clients. In addition to providing treatment for potentially life- threatening complications, the nurse-midwives counsel clients about FP and contraceptive options and provide or refer for methods to help clients prevent future unwanted pregnancies and practice birth spacing. The nurse-midwives also offer selected reproductive and other health services either at their health facilities or via referral to another facility accessible to the client. | Mixed-methods | People-Centred, Timely | Timely: Patient turnover and waiting times were noted as factors that influenced the quality of care and professionalism  People-Centred: Most providers scored well on availability of drugs and attitudes of staff. Quality of care and professionalism displayed by the medical staff have a bearing on choice of health facility. | Weak |
| (Gill and Carlough 2008) [15 countries] | To review literature on mission hospitals providing maternity care | Global literature | Delivery of quality care by the private sector | Literature Review | Timely, Effective | Effective: As they have gained a reputation for prompt and effective response to emergencies, FBO hospitals receive a higher percentage of obstetric complications than government facilities; This was attributed to the lack of training and the low morale of many government health workers. A comparison of obstetric services in government and mission facilities within the same districts in Malawi, Uganda, and Ghana found that all 6 mission facilities outscored their government counterparts in use of standard protocols for laboratory tests, prenatal care, pelvic and other physical assessments, and use of partograms  Timely: In Tanzania, the quality of EmOC at mission hospitals is also recognized by the community. As they have gained a reputation for prompt and effective response to emergencies, FBO hospitals receive a higher percentage of obstetric complications than government facilities | Weak |
| (Haemmerli, Santos et al. 2018) [Uganda, India] | To assess the socio-economic profile of clients of three maternal health social franchises | 2188 women | Delivery of quality care by the private sector | Descriptive Statistics | Equitable | Equitable: Although aimed to serve poorer groups, the franchise users were concentrated in higher wealth quintiles. Content of care did not vary by socio-economic status | Weak |
| (Hansen, Peters et al. 2008) [Afghanistan] | To describe the level of quality of care provided by agencies implementing basic health services in Afghanistan | 5597 exist interviews and 5719 direct observations at 617 health facilities | Delivery of quality care by the private sector | Regression analyses | Equitable, People-Centred | Equitable: For patient history and physical examinations of patients aged 5 years, the poor receive a higher level of service quality than the non-poor in non-governmental facilities, while there is no difference in government facilities  Remoteness, Facility type, provision of timely salary payments and in-service training were found not to be associated with quality  People-Centred: The quality of provider–patient communication was lower for the poor than the non-poor in government managed facilities, while there is no difference in non-governmental facilities. | Weak |
| (Health Partners International and Montrose 2014) [Uganda] | The study had three objectives: 1. To compare differences in the use of partographs between the RBF and IBF health facilities and assess whether these are associated with perinatal outcomes. 2. To compare differences in the use of partographs between the RBF and IBF health facilities and assess whether these are associated with occurrence of emergency Caesarean Sections 3. To compare differences in prescription practices between the RBF and IBF health facilities for common childhood illnesses (malaria, pneumonia and diarrhoea) and assess the overall management of sick children. | 21 faith-based, private not-for-profit health providers in Acholi sub region and ten in control area in Lango sub region | Northern Uganda Health Programme (NU Health 2011-2015) aimed to strengthen local and national mechanisms for governance and accountability and improve access to healthcare through the use of results-based financing. | Cohort analytic and regression analyses | Effective | Effective: There was no significant association between the occurrence of an emergency CS and complete monitoring during labour for mothers in the RBF region for the baseline (OR 0.17 CI 0.02-1.38) and first year of implementation (OR 0.42 CI 0.11-1.66). Similarly, there was no association between the occurrence of an emergency CS and any monitoring (including partial and complete) during the baseline (OR 0.94 CI 0.12-6.91) and first year of implementation (OR 0.57 CI 0.17-1.9) in the RBF region  Safe: Diagnosis and treatment for many conditions were stronger under RBF. While NU Health was not the only health intervention potentially influencing health outcomes in the two regions, a clinical audit showed that after two years of programme implementation and after adjusting for confounding factors, a child in the RBF region was three times more likely to be treated correctly for malaria than a child in the IBF region; almost seven times more likely to be treated correctly for pneumonia; and over eight times more likely to be treated correctly for diarrhoea. It should be noted, however, that there was also a time lag in the comparative improvement in RBF facilities, which would reiterate the fact that RBF can take a year or two to show results, but then show a marked advantage as compared to IBF | Weak |
| (Health Partners International and Montrose 2015) [Uganda] | To summarise the issues around quality of care noted throughout the life of the Northern Uganda Health programme and provide lessons for policy makers | 31 faith-based, private not-for-profit healthcare providers | Health facilities received a Quarterly Quality Assessment (QQA) and an annual assessment of staffing to assess and monitor the quality of care provided. Direct Client Verification of care received was also conducted to compare different services. | Multivariate analysis | Safe, Effective | Effective: On clinical assessments, there was an improvement among all health facilities in the RBF group from the assessment in the first round to the second round, which then declined slightly in the third round for HC3 and hospitals. In the IBF health facilities, improvement between the first and second rounds was observed only at HC2 and HC3 level of care but not in hospitals  Safe: Two years of programme implementation and after adjusting for these factors, compared to a child in the IBF region, a child in the RBF region was:  Three times more likely to be treated correctly for malaria;  Almost seven times more likely to be treated correctly for pneumonia; and,  Over eight times more likely to be treated correctly for diarrhoea. | Weak |
| (Health Partners International and Montrose 2015) [Uganda] | To explore the impact, cost, and benefits of response based funding relative to input-based funding | 31 faith-based, private not-for-profit healthcare providers | Northern Uganda Health Programme (NU Health 2011-2015) aimed to strengthen local and national mechanisms for governance and accountability and improve access to healthcare through the use of results-based financing. | Cohort analytic and regression analyses | Timely, Safe, Effective, Quality Scores | Effective and Safe: While NU Health was not the only health intervention potentially influencing health outcomes in the two regions, a clinical audit showed that after two years of programme implementation and after adjusting for confounding factors, a child in the RBF region was three times more likely to be treated correctly for malaria than a child in the IBF region; almost seven times more likely to be treated correctly for pneumonia; and over eight times more likely to be treated correctly for diarrhoea. It should be noted, however, that there was also a time lag in the comparative improvement in RBF facilities, which would reiterate the fact that RBF can take a year or two to show results, but then show a marked advantage as compared to IBF.  Timely: At the end of year two, it was found that some health practices were lagging, and so the weighting of certain quality indicators was adapted to promote improvement in these practices. These related, for example, to early and regular ANC visits  Quality Scores: Quality scores were higher in the RBF facilities. QQA scores, which were incentivised under RBF, increased from 62% to 81% from 2013-2014 in the RBF region, and from 48% to 67% in the IBF region, with the highest increases occurring at Health Centre 3 level. | Weak |
| (Hirose, Yisa et al. 2018) [Nigeria] | to assess the technical quality of maternal healthcare, in particular intrapartum care, from a health system and programmatic perspective, with particular reference to the public sector and the formal private-sector | 468 case records | Delivery of quality care by the private sector | Descriptive Statistics | Safe | Safe: In Enugu, the median Safe Attendance Index (SAI) varied significantly according to the type of facility. In the public and private not-for-profit sectors, the SAI was greatest in the highest level of care and 81.0% for private not-for-profit comprehensive emergency obstetric care (CEOC).  At the same level of care, the private not-for-profit sector tended to do better than the public sector, e.g. the median SAI for private not-for-profit BEOCs was 76.2% higher than the public BEOCs’ 66.7%. Private for-profit BEOCs fared worse than public BEOCs with a score of 52.4%. The SAIs at the PHC level were low across all the sectors. Similar trends were observed when data were stratified by mode of delivery.  In Lagos, the median SAIs varied across different types of health facilities, but to a much lesser extent than in Enugu. As in Enugu, the higher the level of care in Lagos, the higher the median SAI in the public and private not-for-profit sector. Sector differentials were minimally observed at the CEOC level in Lagos, but at the PHC level, private for-profit PHCs appeared to outperform the public and private not-for-profit sectors | Weak |
| (Huda, Ahmed et al. 2015) [Bangladesh] | To adapt and implement a set of process indicators, bsaed on the safe abortion model (SAC), to supplement the tools and indicators for monitoring emergency obstetric care interventions | 74 registered health facilities that provide menstrual regulation, legal abortion, and / or post-abortion care services in Jessore | Training of staffs from participating facilities on the new SMRAC model and introduction of the tool in all selected facilities. Regular visit of project staff to the facilities for monitoring and helping the service providers to use the SMRAC model (May-July 2009). | Quasi-experimental | Safe, Effective | Effective: By the endline, majority of the NGOs (94 %) were providing uterine evacuation with recommended technology compared to public (67 %) and private (33.3 %) facilities  Safe: NGOs performed the majority of safe MR procedures (93 % in the endline) among women receiving abortion services, followed by public facilities (69 %) and private facilities (33 %) | Weak |
| (Hulton, Matthews et al. 2007) [India] | To present evidence of a situation analysis of quality of care within institutional maternity services | 4 public providers of maternity health services | Delivery of quality care by the private sector | Case study analysis | Safe, Effective | Effective: The situation analysis reveals the routine use, at both public and private establishments, of procedures which are not evidence-based and which do not comply with the WHO’s guidelines on care of women in labour (WHO, 1996). Routine enemas, pubic shaving, episiotomies for first births were hospital policy in all hospitals  Safe: In private clinics, there were usually no blood bank on site or close, and no fulltime anesthetic cover. In public hospitals there were incidences of unskilled women helping attend childbirth and the running (cleaning and cooking) of the ward | Weak |
| (J-PAL Policy Briefcase 2019) [Uganda] | To evaluate the Community Health Promoter program by BRAC Uganda | Community health promotors in 214 villages | Comprehensive training on health education and business skills for community health promotors, in order to provide home visits, health education, basic medical advice and treatment, and referrals to nearby clinics, as well as selling health-related commodities. | Randomized controlled trial | Timely | Timely: During the first week after giving birth, households in CHP villages were 8.1 percentage points (71 percent) more likely to receive a follow-up visit from any health care worker, an increase from 11.4 percentage points in the comparison group.  Households also experienced an increase in services that were not directly incentivized. For example, households in CHP villages were 6.1 percentage points (73 percent) more likely to receive follow-up home visits after a child fell sick with malaria, an increase from 8.4 percentage points in the comparison group | Weak |
| (Jayanna, Mony et al. 2014) [India] | To assess facility readiness and provider preparedness for two obstetric emergencies | 131 primary health centres, 74 higher public and 74 private facilities | Delivery of quality care by the private sector | Descriptive statistics | Timely, Effective, Safe | Effective: Providers at higher facilities  performed better than those in the PHCs, and statistically significant differences were observed in knowledge related to uterine atony (p < 0.001), performing speculum examination in cases of PPH with contracted uterus (<0.001), knowledge related to hemoglobin investigations (<0.05), and monitoring vital signs (<0.01). Specialists performed much better than other staff. Staff nurses, who are the major care providers in these settings, were very deficient in knowledge regarding the diagnosis and management of PPH  Safe: Only 33.8% of higher public and 16.2% of private facilities had at least one specialist thus reflecting acute shortage of specialists in the region to deal with emergency care; however these facilities that had an average of 2 specialists per facility. While labour rooms were reported to be highly functional (92%) across all three types of facilities, the private facilities (95%) reported better functionality of operation theatres than public facilities (78%). Only 22% of private facilities had ambulances stationed on their campuses, as compared to 88% of higher public facilities  Timely: Only 33.8% of higher public and 16.2% of private facilities had at least one specialist thus reflecting acute shortage of specialists in the region to deal with emergency care  Only 22% of private facilities had ambulances stationed on their campuses, as compared to 88% of higher public facilities | Weak |
| (Johnson and Cheng 2014) [18 countries] | To analyse the role played by private commercial health providers in providijg HIV testing | DHS and AIDS Indicators Surveys | Delivery of quality care by the private sector | Regression analyses and descriptive statistics | Equitable | Equitable: Wealth quintile was variously related to whether a person was offered an HIV test at ANC check-up. This was not consistent across countries. | Weak |
| (Karki, Ojha et al. 2009) [Nepal] | To conduct a baseline survey on existing government approved comprehensive abortion care services | Abortion care services providers in Nepal, including providers from MSI (Marie Stopes International) Choices and Family Planning Association of Nepal (FPAN) | Delivery of quality care by the private sector | Healthcare centre observations | Safe | Safe: Infection prevention practice in terms of sterilization techniques, instrument cleanliness, surface cleanliness and waste management needed lot of improvement at Government sites in comparison to private sites. Dhadingbesi hospital has been sterilizing the instrument and gloves in Momo pot, Mechi Zonal | Weak* |
| (Khan, Owais et al. 2017) [Pakistan] | To explore how intended intervention components were implemented and experienced by private care providers and clients | 16 intervention clinics and 16 control clinics | A contextualised intervention was developed through a programme-led technical working group. The intervention and trial design are fully described in the published trial protocol. The core intervention was a structured clinic-based, quarterly tool-assisted counselling session for mother–child pairs. Public–private partnership and community advocate activities were undertaken in both arms of the trial. | Mixed-methods | Timely | Timely: in the review of 1242 child intervention records – 34% of mother-child pairs made three or more follow-up visits (max recommendation = 4 visits), 54% came for one or two and 12% did not attend any. Clinical staff calling to remind follow-up was found by staff and families to be acceptable, with occasional issues of phones not in operation, non-adherence of mothers, or clinic timing for appointment not being convenient | Weak |
| (Kohnke, Mukherjee et al. 2017) [China] | To understand how international NPOs enable the long-term delivery of surgical care in underserved communities | 15 key individuals from Children's Heartlink and partner organisations; data from 11 observation years | Children's Heart-Link intervention provides education, technical support, diagnostic and treatment capabilities, and medical equipment to partner organisations, as well as skill development and resources for medical staff. | Mixed-methods | Timely | Timely: Age was positively associated with mortality – with increased affordability meant mean age of patients decreased and the delivery of CHD surgical care to patients at a younger age was done in a timely manner | Weak |
| (Kojima, Bristow et al. 2015) [Haiti] | To reintroduce newborn male circumcision (for non-medical reasons) in Haiti | 2 obstetricians and 7 nurses | Staff were trained on the technique, common complications and management, outcome survey data collection. Training included practice surgeries with newborns. Nurses were trained to ensure participant flow, preparation and education. | Descriptive study | Safe | Safe: The nurse-collected survey indicated that 100% of the procedures followed the Pollock technique protocol | Weak |
| (Lance, Angeles et al. 2012) [Bangladesh] | BSSFP had four objectives: (1) to develop a franchise model; (2) to recover 70 percent  of operational costs by the end of the project; (3) to increase and expand quality service volume; and (4) to  ensure that 30 percent of all health services provided are service contacts targeted towards the poor who  are unable or only partially able to pay | 1) a network of 323 static clinics (193 of which were urban and the remaining 130 rural); 2) 8,800 satellite (outreach) clinics; and 3) 6,000-plus community mobilizing personnel known as  Community Service Providers (CSPs) | The BSSFP, running from 2007 to 2011, aimed to maintain and expand the availability of family planning and maternal–child health services in a manner that reduces reliance on USAID funding for recurrent costs. A defining feature of the BSSFP was attaining cost-recovery as it was conceived at a time when funding for USAID/Bangladesh was expected to decline drastically. The program charged user fees for all services except for  LAPM of contraception and Tuberculosis treatment. At the same time, the participating NGOs had to make  provisions for serving the poor who were unable, or partially able, to pay by offering them subsidized or free  services | Difference in differences | Equitable | Equitable: In rural areas, poorest quintile experienced greatest increase in coverage of ANC – from 27% in 2008 to 37% in 2011. In 2008, the richest quintile was 2.7 times more likely than the poorest to seek ANC from MTP, 2011 this ratio had improved to 1.9. A similar improvement in equity was observed in comparison areas. In urban areas, Over a quarter of all women in urban project areas receiving at least one ANC service had done so from a Smiling Sun provider, although the exact market share declined from roughly 27 to 24 percent between 2008 and 2011. This decline is attributable to a decline in utilization of Smiling Sun satellite clinics from 10.6 to 7.1 percent. However t-test results suggest that the decline was not statistically significant. | Strong |
| (Lee, Madhavan et al. 2016) [Kenya] | To assess the levels and heterogeneity of antenatal care in Kenya | 695 ANC facilities | Delivery of quality care by the private sector | Secondary data quality assessment | People-Centred, Efficient, Safe | Efficient, Safe, People-Centred: Within management authorities, facilities run by faith-based organizations performed consistently well in terms of efficiency, accessibility, and acceptability/patient-centeredness and moderately well on effectiveness and safety | Weak |
| (Levin, Munthali et al. 2019) [Benin, Malawi, Georgia] | To understand the role of private sector provision and financing of vaccination services | In Benin, 295 vaccination clients across 35  private for-profit, 9 faith-based, 6, NGO, and 10 public facilities.  In Malawi, 310 vaccination clients across 16 private for-profit, 21 faith-based, 5 NGO, and 11 public facilities.  In Georgia, 301 vaccination clients across 44 private for-profit facilities, 3 private maternity facilities, and 3 private hospitals. | Delivery of quality care by the private sector | Case studies | Effective, Safe, Timely | Effective: In Malawi and Georgia, most facilities offered the full range of NIP vaccines. In Benin, 88% of facilities offered vaccines given to infants before six months of age, and 70% of facilities offered vaccines that are given after six months, such as measles containing ones. In Benin and Georgia, maternities offer only vaccines required for pregnant women and newborn infants, and a few facilities offer only non-NIP vaccines  Safe: Most private facilities were accredited by regulatory bodies in their countries. There was more variation in the frequency of regulatory visits and supervision. The most frequent regulatory visits were reported in Benin (84% in last year) and the least in Georgia (42% in last year). Health providers reported they were supervised most frequently in Malawi (78% monthly or quarterly) and the least often in Benin (54% monthly or quarterly).  Timely: Most private facilities were accredited by regulatory bodies in their countries. There was more variation in the frequency of regulatory visits and supervision. The most frequent regulatory visits were reported in Benin (84% in last year) and the least in Georgia (42% in last year). Health providers reported they were supervised most frequently in Malawi (78% monthly or quarterly) and the least often in Benin (54% monthly or quarterly). The majority of private facilities reported that they stored vaccines, ranging from 60% in Benin to 98% in Georgia. Among the private facilities that stored vaccines, most had cold-chain equipment that met the national standards (i.e., equipment was either a WHO pre-qualified brand or was a brand approved by the MoH). The percentage of facilities that did not meet standards was lowest in Benin (17%) and highest in Malawi (29%) | Weak |
| (Lind, Edward et al. 2011) [Afghanistan] | To provide a baseline measure for hospital quality of care | 31 hospitals | Delivery of quality care by the private sector | Regression analyses | People-Centred, Timely, Effective, Equitable, Safe | Effective: Hospital training not significant when controlling for hospital type and patient sex. Care provided by a physician had a 2.9 times greater odds of better quality as compared with other providers (95% CI: 1.2 –6.7, P ¼ 0.01). Other hospital capacity and management variables were not significant.  Equitable: Even after adjustment for hospital type and patient sex, the odds of quality care provided by a female provider compared with a male was 5.8 times higher (95% CI: 1.9 –17.7, P ¼ 0.002).  There was no difference in the odds of high quality care being received by the poor and non-poor in MOPH facilities. However, in the hospitals managed by NGOs, the poorest patients had a 10.4 times greater odds of receiving high quality care than the non-poor did (95% CI: 1.4 –75.2, P ¼ 0.02). The poor are more likely to receive better quality care at a NGO-managed hospital than hospitals managed by the MOPH (OR: 15.2, 95% CI: 1.2 –200.1, P ¼ 0.039)  Safe: Results of the quality of care assessments are summarized in Table 3. Assessing danger signs was variable, with 45% assessed for the ability to drink or breastfeed, 42% for vomiting and 20.1% for convulsions. Diarrhoea was queried in 65% of children, fever in 77.5% and cough in 59.3% with 36.2% assessed for three symptoms. 56% of the children weighed, had their weight checked against a growth chart. The caretakers were counselled about feeding during illness in only 18% of consultations, but 87% of children requiring vaccination were sent for immunization. In the exit interview, 56% of the caretakers were able to provide correct responses on how to administer medication at home. The overall quality of care median score was low: 27.5, on a 100-point scale.  Timely: Being less than two hours travel time to the hospital was associated with 2.1 times the odds of receiving higher quality care  People-Centred: The caretakers were counselled about feeding during illness in 18% of consultations; 87% of children requiring vaccination were sent for immunization. In the exit interview, 56% of the caretakers were able to provide correct responses on how to administer medication at home. The overall quality of care median score was low: 27.5/100 | Weak |
| (MacFarlane, O'Neil et al. 2017) [Turkey] | To document both married and unmarried women’s experiences obtaining abortion services in Istanbul | 14 people who had obtained abortions | Delivery of quality care by the private sector | Qualitative: in-depth interviews and case studies | Safe, People-Centred | Safe: Women at private facilities reporting being satisfied with quality and cleanliness, whereas women in public facilities did not report this  People-Centred: Almost all of the women who obtained abortion care in the private sector reported being satisfied with the care they received including the background information about the procedure, pain management, quality and cleanliness of the facility, and interactions with medical personnel. In contrast, women who obtained their abortion care at a public hospital were generally dissatisfied. As exemplified by Dilan's story, these women described feeling judged by public health service personnel, lacking privacy and receiving inadequate pain management | Moderate |
| (Mahar, Kumar et al. 2012) [Pakistan] | To assess the quantity and quality of information, education and communication during antenatal care in public and private hospitals of Bahawalpur | Pregnant women attending obstetric outpatient departments at a private hospital (n=108) and a public hospital (n=108) | Delivery of quality care by the private sector | Cross-sectional | People-Centred | People-Centred: A higher proportion of private (43%) than public (18%) clients received information regarding danger signs of pregnancy, delivery and postnatal. 13% of women got information for family planning in public facility but only 7% in private facility. None of antenatal clients in both settings received any kind of education and advice for care of baby after birth or breast feeding in both facilities | Moderate |
| (Maru, Maru et al. 2017) [Nepal] | To describe the impact of the care reforms to improve women’s and children’s health | Healthcare providers in Achham district, rural Nepal | A public-private partnership that included community health workers serving villages, open-source electronic health records to monitor care and link community health worker data to hospital data | Controlled pre-and-post analysis | Safe | Safe: The rate of institutional birth (proxy for safety) increased from 80.7% to 92.5% - statistically significant. Completion of antenatal care increased from 83.4% to 89.8% | Moderate |
| (Mbonye, Buregyeya et al. 2016) [Uganda] | To assess quality of care in the private sector for patients seeking care in this outlet | 57 parishes with 242 private facilities | Delivery of quality care by the private sector | Regression analyses | Safe, Effective | Effective: Treatment of pregnant women with fever according to the guidelines was 40.7 % at private clinics, 28.2 % at drug shops and 16.7 % at pharmacies. The following factors were associated with correct treatment given to pregnant women with fever: the type of facility (drug shop), P = 0.04; knowledge of people vulnerable to malaria, P = 0.02; and availability of a malaria treatment guideline, P = 0.03  Safe: Treatment of pregnant women with fever according to the guidelines was 40.7 % at private clinics, 28.2 % at drug shops and 16.7 % at pharmacies. A big proportion, 45.9 % of drug shops prescribed SP as first-line treatment for malaria to pregnant women; while 2.9 % prescribed ACT. The majority of providers (>75 %) at all private facilities prescribed SP for intermittent preventive treatment (IPTp) but artemisinin-based combination therapy (ACT) was also prescribed: 8.3, 6.9 and 8.3 % respectively at drug shops, private clinics and pharmacies for prevention of malaria in pregnancy  The following factors were associated with correct treatment given to pregnant women with fever: the type of facility (drug shop), P = 0.04; knowledge of people vulnerable to malaria, P = 0.02; and availability of a malaria treatment guideline, P = 0.03 | Weak |
| (McIntosh, Grabowski et al. 2015) [Lesotho] | To compare measures of capacity, utilization, clinical quality, and patient outcomes before and after the implementation of a health care public-private partnership | 36 key informant interviews with healthcare providers and ministry workers; observational data from healthcare facilities. | Delivery of quality care by the private sector | Mixed-methods | Timely, Effective | Effective: Only one emergency crash cart was available at the government- managed hospital in the casualty unit, and it was not accessible within four minutes' travel time from any of the four medical wards. At the PPP managed hospital, each medical ward and the casualty unit had an assigned crash cart, and all were accessible within one minute's travel time from patient rooms. On average, percent of the carts were fully equipped. Nursing staff members did not routinely triage patients in the casualty unit at the government managed hospital. Instead, a nonclinical administrative staff member used his or her judgment to prioritize patients' care needs. In contrast, percent of patients were triaged by nursing staff members within five minutes of arrival in the casualty unit at the PPP-managed hospital.  International best practices include having thrombolytic agents available for patients with myocardial infarction and ischemic stroke. The government-managed network did not stock thrombolytic agents. In contrast, the PPP managed network stocked recombinant tissue plasminogen activator for stroke and streptokinase for myocardial infarction.  Timely: The average turnaround time for laboratory tests at the PPP-managed network was forty-nine minutes. The baseline evaluation of the government-managed network could not assess this measure because relevant data were not collected in laboratory records | Weak |
| (Mendez and Associates 2014) [Georgia] | To evaluate the flexibility, effectiveness, impact, contribution, satisfaction and sustainability of an MNCH facility intervention | 56 health facilities providing maternal, newborn and child health throughout Georgia | The SUSTAIN intervention was intended to extend and sustain MNCH services through capacity building of networks of private facilities and providers, as well as adding new technical approaches including management of third stage labour, preeclampsia and eclampsia, postpartum haemorrhage, pregnancy-induced hypertension, and neonatal resuscitation. | Quasi-experimental | Effective | Effective: Government officials, health facility managers, and representatives of OB/GYN professional networks (who are also facility managers, practitioners and trainers) were unanimous in their praise for SUSTAIN’s positive impact on MNCH and FP health services at target sites through their support for introducing and institutionalizing evidence-based health interventions.  Interviews conducted by the ET at family medicine centres confirmed that providers were quite knowledgeable and effective in providing contraception counselling and advice on appropriate contraceptive selection; These positive assessments were corroborated in the survey that the ET conducted with health professionals, in which 51 respondents from SUSTAIN’s participating medical institutions, vs 39 from non-participating facilities, reported that effective newborn care is available at their facilities (Chi-Square 4.83, p<.03) | Weak |
| (Ministry of Health (MOH) [Zambia], Central Statistical Office [Zambia] et al. 2006) [Zambia] | Zambia HIV/AIDS Service Provision Assessment Survey 2005 | 430 facilities | Delivery of quality care by the private sector | Facility survey | Timely | Timely: Clinical care and support services are available in almost all facilities (97 percent), with little variation by type of facility, managing authority, and province  PMTCT services are available in one-fifth of the facilities (19 percent). The services are more likely to be available at hospitals (62 percent) and urban health centres (47 percent) than in rural health centres and other types of facilities. Only 8 percent of all facilities in Luapula and Northern provinces offer PMTCT services | Weak |
| (Mohanan, Babiarz et al. 2016) [India] | To evaluate the impact of the World Health Partners Sky programme | 36,315 children under five in twelve districts across the state of Bihar in 2011; 31,635 in 2014 | SkyHealth telemedical facilities were established in villages with internet connectivity, to allow patient consultations and remote assessments of certain conditions. Providers who have facilities with telemedicine technology were franchised alongside partnerships with SkyCare providers and rural healthcare providers. All received training on basic service delivering, access to marketing services, and a predictable supply of brand-name adequate-quality drugs | Case-Control | Effective | Effective: No effect on improving appropriate treatment for childhood diarrhoea or pneumonia. | Weak |
| (Mohanan, Giardili et al. 2017) [India] | Investigate the effectiveness of the World Health Partners’ Sky Program | 395 providers at baseline and 415 at follow up | SkyHealth telemedical facilities were established in villages with internet connectivity, to allow patient consultations and remote assessments of certain conditions. Providers who have facilities with telemedicine technology were franchised alongside partnerships with SkyCare providers and rural healthcare providers. All received training on basic service delivering, access to marketing services, and a predictable supply of brand-name adequate-quality drugs | Quasi-experimental difference in difference | Effective | Effective: At follow-up, providers in implementation areas were seven percentage points more likely to prescribe the correct treatment in response to the pneumonia vignette than those in non-implementation areas (intergroup difference: 0.070; 95% CI: 0.015 to 0.129) and seven percentage points less likely to prescribe a harmful treatment (intergroup difference: –0.070; 95% CI: –0.127 to –0.011). These estimates were comparable to point estimates in the difference-in-difference analysis though the latter were not statistically significant | Weak |
| (Mony, Krishnamurthy et al. 2013) [India] | To study the availability and distribution of emergency obstetric care services | 444 government and 422 private health facilities | Delivery of quality care by the private sector | Cross-sectional | Safe | Safe: Among the 16 taluks without adequate EmOC/CEmOC facilities, it was seen that 15 (94%) did not also have the minimum desired number of 4 BEmOC facilities per 500,000 population. It was also noted that 70% of BemOC facilities were in the private sector  None of the 42 taluks had an adequate number of EmOC or BEmOC facilities in the government sector. The private sector however, contributed substantial amount of EmOC services in 15 taluks. Within this entire region, a subset of about a dozen contiguous taluks with insufficient number of EmOC and BEmOC facilities in the government sector were spatially clustered in northeastern Karnataka across the districts of Raichur, Yadgir and Gulbarga | Weak |
| (Murphy, Gathara et al. 2018) [Kenya] | To describe the provision of and access to inpatient neonatal services within Nairobi City County, and to explore access and evaluate readiness of public, private not-for-profit, and private-for-profit sector facilities | 31 facilities in Nairobi City County that provide inpatient neonatal care | Delivery of quality care by the private sector | Cross-sectional | Safe | Safe: Safe delivery equipment and drugs for mothers on the delivery ward was the weakest domain (average availability score of 67.8% in all sectors [public score = 80.4%; mission = 78.8%; private = 62.0%]) | Weak |
| (Murphy, Gathara et al. 2018) [Kenya] | To report on the quality of the process of care delivered to small and sick inpatient newborns across diverse facility settings in Nairobi | 33 facilities in Nairobi City County that provide inpatient neonatal care | Delivery of quality care by the private sector | Retrospective review of medical records | Safe | Safe: Almost two-thirds (63.4%) of the population were prescribed antibiotics, of whom the majority (92.6%) were prescribed both gentamicin and penicillin, suggesting good adherence to recommended first-line treatment across sectors. A large proportion (62.5%) of newborns who had no admission diagnosis of severe infection (n = 821) were nonetheless prescribed antibiotics. Incorrect doses were prescribed to 19.4% of newborns. An overdose of gentamicin was the most common error (11.7%) | Weak |
| (Nelson, Corbett et al. 2002) [Kenya] | To assess the PRIME post-abortion care programme in Kenya | 155 nurse midwives at 120 facilities across six districts of the three pilot project provinces | An intervention aimed at training private nurse-midwives, focused on 13 key components: introduction and clarification of values, client-provider interaction and counselling, management of complications from unsafe or incomplete abortion, MVA procedures, infection prevention, pain management, postabortion family planning counselling and method provision, STI/HIV management, record keeping, legal aspects of providing PAC services, peer supervision, community outreach, performing practical procedures. | Mixed-methods | People-Centred, Effective, Safe | Effective: During phase two, PRIME’s Nairobi office took on a more extensive role in providing support after training and managed to visit 82% of the trained nurse-midwives at least twice. While providers reported that those visits were very helpful in reinforcing skills, ensuring that infection prevention procedures were being followed, and checking on supplies, PRIME did not have adequate staff to provide sufficient post-training support  Safe: As nurse-midwife Milka Mathea of the Jamii Medical Clinic in Namanaga says, “PAC is helping very much. It has improved the management of clients, not only those with incomplete abortion but also others. Generally, the cases of clients coming with incomplete abortion have gone down. I visited the government health centre here and I also learned that the abortion cases are fewer there”  People-Centred: 81% of people receiving PAC received counselling for family planning | Weak |
| (Nikniyaz, Farahbakhsh et al. 2006) [Iran] | To implement and evaluate a new model for transferring state service delivery governance to non-governmental groups | 9 cooperative health centres and 18 public health centres | Services at government facilities were contracted out to the private sector | Case-control | Timely, People-Centred | Timely: Cooperative health centres had significant better indicators of availability of services than public health centres  People-Centred: Cooperative health centres had better indicators than public health centres on friendly attitude of personnel and giving sufficient and suitable education | Weak |
| (Pathfinder International 2016) [Bangladesh] | To explore performance-based grants implementation | 25 NGOS and 388 clinics | In 2012, Pathfinder began implementation of the NGO Health Service Delivery Project—a five-year, USAID- and DFID-funded project that aims to strengthen NGO capacity to deliver high-quality family planning and reproductive, maternal, newborn, and child health services to the country’s poor and underserved populations. Complementary to capacity building activities, and to catalyse improved NGO performance, Pathfinder  implemented performance-based grants. | Mixed methods | Equitable | Equitable: The percent of services provided to those who qualify as poor increased from 35 percent in Year 1 to 42 percent in Year 3. | Weak |
| (Penn-Kekana, Powell-Jackson et al. 2018) [India] | To report evaluation findings and assess the impact of the Matrika social franchise programme in Uttar Pradesh | 365 SkyCare providers, 50 SkyHealth centres, 8 franchise clinics, 58 private providers, 50 private facilities, and 2,149 accredited social health activists | The intervention aimed to improve maternal health through reducing deaths from postpartum haemorrhage by establishing the Sky social franchise network. This engaged private providers at three levels:  1. Skycare providers: informal rural healthcare providers, who were trained to encourage women to use the services in the network and facilitate phone consultations.  2. SkyHealth centres: engaged to provide ANC consultation using telemedicine.  3. Franchise Clinics: private hospitals engaged to deliver emergency obstetric care.  Clinical training and regular quality improvement visits were conducted with SkyHealth centres and Franchise Clinics, and village led information activities were designed to engage women. | Mixed-methods | Effective, Safe | Effective: All Sky providers reported receiving training. Implementation of the supervision was, however, patchy, with only 45% of providers receiving a visit in the previous  6 months.  However, other data suggest that monitoring and supervision visits were unlikely to have improved quality of care in Sky facilities. Qualitative responses were mixed about the adequacy of visits from WHP staff to support clinics in the SkyHealth facilities. While they acknowledged that franchisor staff visited them regularly, they said that generally these visits did not concern quality—rather WHP staff came to monitor data collection (i.e., names of maternal health patients who attended the clinic).  Safe: Data suggest that monitoring and supervision visits were unlikely to have improved quality of care in Sky facilities. Qualitative responses were mixed about the adequacy of visits from WHP staff to support clinics in the SkyHealth facilities. While they acknowledged that franchisor staff visited them regularly, they said that generally these visits did not concern quality—rather WHP staff came to monitor data collection (i.e., names of maternal health patients who attended the clinic) | Strong |
| (Powell-Jackson, Macleod et al. 2015) [46 countries]] | To examine the role of the private sector in the provision on ANC in LMICs | Demographic and Health Survey data | Delivery of quality care by the private sector | Regression analyses | Effective | Effective: A large proportion of women given ANC at home received few of the individual components, with a mean score of 0.28. While differences between public, private commercial and private not-for-profit exist, they were not substantial. The private not-for-profit sector has the highest ANC score (0.71), followed by private commercial (0.63) and public providers (0.59). Clearly, there is substantial room for improvement across all sectors. There was a large spread in ANC quality, irrespective of provider type, with a long tail suggesting that a large pro- portion of women received poor quality of care  Wealthier women consistently received a higher pro- portion of ANC components during pregnancy. Second, the gap between wealth quintiles in the content of care was greater amongst private commercial providers than those in the public or private not-for-profit sectors, per- haps reflecting the diversity of providers operating in the private commercial sector. Only in one region, South/ South-East Asia, was this not the case. Third, the private not-for-profit sector consistently provided the highest quality of care, with the least variation between wealth groups | Strong |
| (Powell-Jackson, Penn-Kekena et al. 2018) [India] | Presents findings from an impact and process evaluation of the Matrika programme | 7054 women who gad recently given birth | The Matrika programme had three components: (1) establish the Sky social franchise network of private health-care providers and functional referral centres;  (2) strengthen capacity of, and linkages between, rural private and public sector health-care providers to offer high-quality services; and (3) improve community awareness, demand, and linkages with maternal health services among rural populations. The intervention was implemented in three districts of Uttar Pradesh by World Health Partners (the franchisor) in partnership with Pathfinder International,  between 2013 and 2016. | Household survey | Effective | Effective: Most SkyCare providers had limited experience working in the area of maternal health | Weak* |
| (Quimbo, Peabody et al. 2008) [Philippines] | To measure the quality of paediatric care provided by private and public doctors working at the district hospital level | 30 district-level hospitals | National level health provider accreditation by a national heath insurance program | Mixed-methods | Safe, Effective | Vignettes require the doctor to answer open-ended questions about a typical paediatric patient. The physician responses are scored and used to examine the clinical decisions made by physicians in five domains of care: (i) taking a medical history, (ii) performing a physical exam, (iii) ordering tests, (iv) making a diagnosis and (v) prescribing a treatment plan. Each physician answered three vignettes, one for diarrhoea, one for pneumonia, and a third one on a common dermatologic condition.  Effective: Accreditation was associated with improved answers to evaluation vignettes  Safe: The coefficients suggest that insurance payments potentially affect quality for private doctors more than accreditation. A private doctor receiving PhilHealth payments has an average vignette score 8 percentage points higher, while an accredited doctor’s average vignette score is on the margin 6 percentage points higher than those without PhilHealth accreditation. The actual amount of the payment has no influence on quality in the models |  |
| (Rahman, Rob et al. 2009) [Bangladesh] | To develop a voucher distribution system for women using maternal health services, identifying private and NGO facilities to provide quality services, increase capacity of service providers and improve utilisation | A survey of providers from 3 clinics and 23 fieldworkers  A survey of pregnant women/mothers (n=436) and in-depth interviews with women (n=15) | The intervention provided a 3.5-day orientation for service providers, capacity building of service providers and fieldworkers including three-week training in partnership with Population Council, health facility strengthening to enable the provision of quality services, flipcharts and educational materials to create awareness of the intervention activities amongst communities. | Pre-and-post intervention | Efficient, People-Centred, Timely | Efficient: Information about where to go for pregnancy-related complications also increased significantly, as did the proportion of women requested to make a follow-up visit. Use of educational materials increased tremendously, although the proportion remained low at less than one third. The proportion of women claiming that service providers delayed providing services significantly reduced after introduction of the vouchers.  Timely: The proportion of women claiming that service providers delayed providing services significantly reduced after introduction of the vouchers.  People-Centred: Three quarters of women reported being treated in a friendly manner at baseline, which increased to 96% at endline. Information about pregnancy-related complications increased significantly | Weak |
| (Ramachandar and Pelto 2002) [India] | To report on abortion services and the role of government village health nurses in assisting women to obtain abortions | 42 village health nurses from 10 sterilisation/MTP camps in different primary health centre locations | Delivery of quality care by the private sector | Qualitative interviews | Safe, Effective, People-Centred, Timely, Equitable | Effective: Unqualified abortion providers were also well known to VHNs, women and government health personnel. They had not received medical training but had acquired their skills from apprenticeship and participation in medical settings, and in some cases from training in homeopathic and siddha (traditional) medicine. Most of them were practising medicine, including doing abortions, quite openly  Safe: The operating surgeons and medical doctors deputed to the camps appeared to be quite concerned about maintaining adequate levels of quality of care, particularly in the actual surgical procedures. Nonetheless, MTP operations were carried out.  In many cases the PHC medical officer’s consulting room had been converted into a temporary ‘‘operating theatre’’. As operating theatres, these were in many ways substandard as there was a lack of blood transfusion facilities, they were generally poorly lighted and many lacked running water. Although cleanliness was difficult to maintain, PHC personnel appeared to expend considerable effort to clean and disinfect the operating tables, and to fumigate the operating rooms. However, the sites usually lacked beds for post-operative recovery, and patients had to lie on mats on the floor. Toilet facilities were generally extremely poor. In one case they observed a major power cut that stopped surgical operations for several hours as the back-up generator was not working. Lack of personnel in relation to the number of cases also added to the stress.  Timely: A major power cut was observed as stopping surgical operations for several hours as the back-up generator was not working. Lack of personnel in relation to the number of cases led to reports of increased stress  People-Centred: There were a lack of beds and poor sanitation facilities, as well as a lack of privacy. women who wanted an abortion had to accept a sterilisation as a precondition, with rare exceptions. Married women who did not want to be sterilised would therefore not resort to the government health facilities, though recent changes in government policies have altered this practice | Weak |
| (Ramachandar and Pelto 2004) [India] | To report on abortion services and the role of government village health nurses in assisting women to obtain abortions | 97 married women who had had abortions within the previous six months and 18 village health nurses | Delivery of quality care by the private sector | Qualitative interviews | Safe, People-Centred, Timely, Effective | Effective: Their average rankings ranged from 1.33 to 2.71 on the five-point scale. All of these providers had medical degrees, and most were gynaecologists. Almost all of them worked in private hospitals with 15–30 beds, modern equipment, staffed by specialised medical doctors including gynaecologists, anaesthetists, surgeons and radiologists. These facilities provided childbirth services, family planning and other reproductive health services. Most had X-ray machines, and 12 had ultrasonography equipment.  Equitable: Ability to access quality care was predicated by many women on their finances and capacity to pay fees.  Some respondents indicated that the chose care as the doctor did not discriminate among patients: The doctor is extremely polite and does not discriminate among patients. I like this doctor. I went to her for the first time for my childbirth. She is very impressive and gentle in treating people  Safe: Nineteen of the 36 abortion facilities fell into the category of highly qualified and safe, but none of the government facilities were included in this top category. The bottom end of the spectrum of abortion services included a medical doctor, a government facility, a pharmacist and several unqualified, untrained practitioners. Some of these providers had very small clinics in congested marketplaces or in crowded interior lanes. Except for two providers in this group, all were using allopathic methods, particularly dilatation and curettage (D&C) with medications such as prostaglandin gel and antibiotics. The facilities were considered unhygienic by the VHNs, who said the practitioners lacked both qualifications and skills, and had minimal medical experience or training.  Timely: Respondents reported waiting for a long time at government hospitals, or developing complications due to delays, and preferring private clinics for timeliness  People-Centred: Respondents reported negligent care and poor follow up that led to complications at government and intermediate facilities, as well as abusive behaviour. | Weak |
| (Rannan-Eliya, Wijemanne et al. 2015) [Sri Lanka] | To assess quality of clinical care | 1027 public and 944 private primary care outpatients | Delivery of quality care by the private sector | Significance tests | Effective | Effective: The overall quality aggregate was the same in both sectors (65%). Performance was mixed for aggregate scores by patient condition, with the public sector performing better for patients with diarrhoea, cough and asthma, while the private sector performed better for patients with hypertension, diabetes and URTI. However, none of these differences were statistically significant, with the exception of diabetes (P<0.01). The public sector performed better than the private sector in the domains of history taking (72 vs 65%) and investigation and management (72 vs 63%), but only the latter was statistically significant (P < 0.05). The public sector performed much better in patient examination (86 vs 69%; P < 0.001). A much larger opposite difference was seen in patient education, with private sector patients far more likely to receive information and education on their diagnosed condition and its management than public sector patients (57 vs 12%; P < 0.001).  Equitable: Those who were near Coimbatore city took their cases to the District Central Hospital, particularly if the women were from low-income households and could not afford to pay the fees in the private sector | Moderate |
| (Rannan-Eliya, Wijemanne et al. 2015) [Sri Lanka] | To assess quality of inpatient clinical care | 10 public hospitals and 66 private practitioner clinics | Delivery of quality care by the private sector | Significance tests | Safe, Overall Quality Aggregate | Safe: No significant differences in quality scores that for correct antibiotic, no sepsis, and ICU care indicators  Overall Quality Aggregate: The public sector performed significantly better than the private sector in the overall quality aggregate (77 vs 69%, respectively). Performance was similar in both sectors for the management of AMI (68%) and childbirth (89%), and slightly better in the private sector for management of asthma (63 vs 69%), although this was not significant. | Moderate |
| (Ridde, Diarra et al. 2009) [Niger] | Process evaluation of the first stage of an intervention done in Niger | Facilities that provide child and maternity care | HELP (Hilfe zur Selbsthilfe e.V.) intervened to organize an intervention aimed at abolishing user fees in two districts to increase service utilization among children under five (curative care and medical evacuation) and pregnant women (prenatal care, deliveries, and medical evacuation). Each of these districts (Mayahi and Tera, 1 000 km apart) has approximately 500000 inhabitants, a district hospital, and around 20 health centres | Mixed-methods | People-Centred | People-Centred: Regarding impacts on their professional practice, more than 80% of the workers stated that they felt personally affected by the implementation of the abolition. 64% stated that this had positive impacts on how they treated patients (improved quality). However, a majority of staff complained of the added administrative and clinical workload | Weak |
| (Rolfe, Leshabari et al. 2008) [Tanzania] | Presents findings on the drivers and inhibitors on the development of non-government provision | Maternity home healthcare providers | Delivery of quality care by the private sector | Case Study | People-Centred, Effective, Equitable, Timely | Effective: One study of antenatal care in Dar es Salaam (Boller et al. 2003) highlighted that technical quality of care is related to the cadre of staff providing the care, and found that 80% of antenatal care in their sample of public facilities was provided by MCH Aides, with only a two-year basic training. The technical quality of care was basic at the maternity homes, but it was similar to that offered by equivalent government facilities. Shortages of basic drugs and equipment were common to both. In the public sector, these were caused by irregular supplies from medical stores, in the private sector by insufficient capital to pre-purchase from commercial sources and lack of access to discounted supplies from government medical stores  Equitable: Differences in technical quality care can be compounded in rural areas by high vacancy rates and low motivation in staff. They found that some of the private maternity homes also were staffed by lower cadres of staff such as MCH Aides when the owner was absent. Such situations tended to occur in the cases where the owner- manager had other professional commitments elsewhere.  Timely: None of the maternity homes had a formal emergency transport plan, but all facilities reported some established method for emergency referral. Transport was much more readily available for those in peri- urban settings—in most cases using public transport (taxi or bus)—and far more limited in rural conditions. The costs of referral were significant and in all cases borne by the client, although some maternity homes reported lending money in emergency cases. Whilst referral for complications was often difficult to accomplish quickly, it was just as difficult for equivalent local government facilities which also lacked their own motor transport, and expected the referred patient to bear the costs of transfer  People-Centred: Personal interactions have important implications for quality of care in pregnancy and particularly in childbirth, but these are often neglected in government facilities. Verbal and sometimes physical abuse by midwives in the public sector featured frequently and consistently in women’s accounts of their care from all the case districts, and it was reported in user focus groups to be a major deterrent to seeking care at the government facilities | Weak |
| (Ross, Mankad et al. 2005) [India] | To improve access and use of quality services | Case study | The Aga Khan Health System, India (AKHS,I), through the Gujarat Health Systems Development Project (GHSDP) aims to support interventions designed to build institutional capacity, strengthen partnerships among key stakeholders, and document and disseminate best practices at local, national and international levels. | Document review, Presentations, Interviews, Site Visits/Observations | Effective | Effective: The physical infrastructure of the HCs was clean and they were well maintained. Staff did report any shortages of drugs, supplies, or IEC materials. Most of the HCs had ample IEC materials, appropriate for the population, displayed in the waiting rooms for clients. The HCs were well organized, infection practices seemed good and registers were well kept. | Weak |
| (Sathar, Singh et al. 2013) [Pakistan] | The research focuses on two major questions: What is the current coverage and quality of post‐ abortion care in the public and private sector? And secondly, how has this changed over the last decade? | 102 health professionals | Delivery of quality care by the private sector | Cross-sectional | Equitable | Equitable: In the under‐served areas studied, there were no such services available at a convenient distance. Access to services is a real barrier to being able to get post‐ abortion complication care, as treatment is only available in towns and cities; Service providers reported that women usually seek treatment of their post‐abortion complications in the private sector with only a few poor women going to government hospitals. According to service providers, the reasons for women frequenting the private sector providers rather than public sector providers are a) the rude behaviour of doctors and other staff at public facilities; b) lack of proper facilities/medicines in the public hospitals; c) the availability of private facilities in the community and the inconvenience of often distantly located government hospitals | Strong |
| (Save the Children 2007) [Malawi] | To provide technical assistance and sub-grants to 15 Malawian non-government organisations (NGOs) to build their capacity to scale up HIV related services. | 15 NGOs | Fifteen local partner NGOs were supported between 2003 and 2007. Support included technical assistance in the development of proposals and budgets, capacity building, management information system improvement, resource mobilisation, community mobilisation training, and behaviour change programmes. | Mixed-methods | Safe | Safe: Three hospitals were assessed against the national standards for infection prevention. Of these, two achieved over 80% of the national standard in all areas assessed. | Weak |
| (Schooley, Mundt et al. 2009) [Guatemala] | To document and assess the validity of anecdotal evidence of the quality of care at the Casa Materna, Guatemala | 21 clients and traditional birth attendants at the Casa Materna, 17 female advocates / promoters of Casa Materna services, and 12 male advocates | As part of the Project Concern International / Guatemala Ministry of Health intervention to reduce maternal mortality, the Casa Materna was established to provide specific maternal, newborn, and child health (MNCH) services through trained providers and increased quality care. Community educators were used to encourage women to utilise these services. | Qualitative: in-depth interviews, focus group discussions and case histories | People-Centred, Timely | Timely: Respondents indicated the difference in experience due to time allowed post birth: “The difference between my two previous hospital births and this one [at Casa Materna] was the quality of care I received and the fact that I could stay at the Casa Materna in this safe place after the birth to recover and be with my baby, whereas at the hospital, I felt rushed in and out and pressured to get well and leave.”  People-Centred: Women and men reported that experiences of resource limitations and high patient load created frustration at Ministry of Health hospitals compared to Casa Materna | Strong |
| (Sharan, Ahmed et al. 2012) [Malawi] | To examine the quality of the maternal health system and the probability of maternal survival at public and private health facilities, including mostly faith-based and a few private-for-proﬁt health facilities | 86 facilities (36 hospitals and 50 healthcare centres) from 10 districts | Delivery of quality care by the private sector | Regression analyses | Effective | Effective: On almost all indicators of health system quality, the private health facilities fared better than the public facilities. Shortages of doctors and Clinical Ofﬁcers existed at both types of facilities, however, private facilities performed better in spite of human resource constraints. Shortages of drugs and equipment were more common in public facilities. Providers were more overworked and less satisﬁed at public facilities. Private facilities were more likely to have a blood bank, laboratory and ambulance onsite and offer caesarean deliveries. Public health facilities were more likely to have experienced problems in handling maternity patient load | Weak* |
| (Sharma, Powell-Jackson et al. 2017) [India] | To describe and investigate the quality of care provided routinely for uncomplicated labour and childbirth in maternity facilities in Uttar Pradesh | 29 private and 30 public maternity facilities in Uttar Pradesh | Delivery of quality care by the private sector | Clinical observations | Effective, Safe, Equitable | Safe and Effective: Quality of essential care during labour and childbirth was found to be deficient (mean: 35.7%) across the entire sample of facilities. Overall, 45.0% of recommended clinical practices were completed among women giving birth in the private sector compared with 33.3% in the public sector (P = 0.01). Private-sector clients received 40.0% of the recommended obstetric care practices and 51.0% of the recommended neonatal care practices – compared with 28.3% (P = 0.01) and 39.0% (P = 0.02), respectively, in the public sector  Equitable: They found no association between use of qualified personnel, facility caseload or the woman’s age, caste, parity, referral status or socioeconomic status and the overall quality of care at the time of birth. However, compared with admission on a weekday, admission during the weekends was associated with a quality of care score that was three percentage points lower | Weak |
| (Sharma, Powell-Jackson et al. 2018) [India] | To describe and investigate quality of care provided in maternity facilities | Clinical observations of maternity and childbirth care | Delivery of quality care by the private sector | Clinical observations | Effective, Safe, People-Centred, Timely | Effective: Overall, 45% of recommended clinical practices were completed among women giving birth in the private sector compared with 33% in the public sector (P=0.01)  Overall, 45% of recommended clinical practices were completed among women giving birth in the private sector compared with 33% in the public sector (P=0.01)  Private-sector clients received 40% of the recommended obstetric care practices and 51% of the recommended neonatal care practices – compared with 28% (P=0.01) and 39% (P=0.02), respectively, in the public sector  Unqualified personnel (dais, other helpers, ASHAs, cleaners) were observed attending to 59% of all deliveries, 65% in the public sector and 41% in the private sector  A number of life-saving clinical practices such as partograph use for monitoring labour, screening for pre-eclampsia/eclampsia and active management of the third stage of labour were rarely observed  The provision of women-centred, respectful maternity care practices was observed in just 4% of deliveries  Poorer quality care was provided during weekends at maternity facilities Private facilities provided marginally better care than public facilities  Timely: Poorer quality care was provided during weekends at maternity facilities. Private facilities provided marginally better care than public facilities  People-Centred: The provision of women-centred, respectful maternity care practices was observed in 4% of deliveries | Weak |
| (Sieverding, Briegleb et al. 2015) [Ghana and Kenya] | To understand experiences with clinical social franchising in three large networks affiliated with INGOs in Ghana and Kenya | 23 providers in BlueStar Ghana. 24 providers in Kenyan franchises (10 from Amua, 14 from Tunza)  21 clients from BlueStar, Ghana. 26 clients from franchises in Kenya (7 Amua facilities, 19 Tunza facilities) | Delivery of quality care by the private sector | Qualitative: in depth-interviews | Effective, People-Centred | Effective: Clients described having confidence in the franchise staff’s ability to help them or their children get better. They valued that franchise staff conducted tests and procedures, and prescribed quality and effective medicine, which was especially important to clients when it came to their sick children.  People-Centred: Clients reported high levels of satisfaction with their visit to the franchised provider for a range of reasons that were closely related to their reasons for choosing to seek care at the facility. These reasons included the perceived quality of medical care received, how polite, friendly and caring the providers and other staff were, short waiting times and facility cleanliness. A number of clients highlighted aspects of the process of receiving care as important factors in their experience. For instance, as a result of the positive relationship clients had with franchise staff, a few clients said that they felt comfortable discussing problems and asking questions of the provider | Strong |
| (Singh, Ahmed et al. 2017) [India] | To evaluate effects of ICDS and RCH program exposure, assessed with respect to coverage, intensity and quality of service provision by Anganwadi workers (AWWs) and Auxiliary nurse midwives (ANMs) on infant breastfeeding and complementary feeding practices | 512 pregnant women in intervention, 468 in control | Intervention program/ Enhanced Integrated Nutrition and Health Program (INHP II)  CARE-India, under its second phase of INHP umbrella adopted a demonstration and replication approach for scaling up the successful practices through government systems, including;  capacity building through technical inputs and training; behaviour change communication through increased equitable and improved coverage along with more frequent contacts; strengthening key systems (eg. supply chain management, and information management); and building community ownership and action. | Comparative cohort study | People-Centred | People-Centred: The quality of contact was defined by the contacts with the service providers and advice received and/or services received during that contact as reported by the mother/child caregiver.  Approximately 30%, 60% and 50% of the study participants in the intervention district reported being contacted by an AWW at the 1st, 3rd and 6-month evaluation visits; but, only 21%, 23% and 6% of the women, respectively, received any breastfeeding advice in the comparison district. In the comparison district, <12% of the participants had any contact with the AWW and <2% reported receiving breastfeeding advice. The quality of contact was significantly higher in the intervention than the comparison area at all three time periods, although less than a quarter of study participants reported being advised regarding breastfeeding practices | Moderate |
| (Tilford and Amjad 2016) [Pakistan] | The five overarching questions addressed in the FSA are:  1. To what extent did the project accomplish and/or contribute to the results  (goals/objectives) stated in the Strategic Work Plan?  2. What were the key strategies and factors, including management issues and policy  environment, that contributed to what worked or did not work?  3. Which elements of the project have been or are likely to be sustained or expanded?  4. What are stakeholder perspectives on the overall project implementation, the policy forums,  and the Learning Agenda implementation?  5. Working around strengthening community-based maternal and newborn healthcare  provision, to what extent has the project been successful? | 95 private sector community midwives | Mercy Corps’ assistance to address some of the underlying issues in the CMW (community midwives) program and the result was a joint project with two purposes: 1) to demonstrate a health impact within three target districts (Quetta, Gwadar and Kech) and 2) to test interventions and provide key lessons for developing an improved CMW program model that the DoH could replicate across the province. | Mixed methods | Effective | Effective: Enhancing quality of care through a clinical skills refresher course: This four-week course was one of the biggest successes of the project. According to those interviewed during the qualitative research, it clearly improved the quality of care the CMWs offer and bolstered their confidence in their skills. The PNC has endorsed the course and it is slated to become standard throughout Pakistan, a lasting contribution from the project  The CMWs supported by the project scored significantly higher with 96.6% providing services with “high competency” compared to 34.5% of CMWs from non- project areas | Weak |
| (Turan, Bulut et al. 2006) [Turkey] | To understand the quality of antenatal and childbirth care at three hospitals in Istanbul | 176 antenatal observations: 43 at the Ministry of Health hospital, 48 at the Social Security Organisation, and 75 at the private hospital | Delivery of quality care by the private sector | Mixed-methods | Safe, Effective, People-Centred | Effective: Women using services at the private hospital reported receiving significantly more information than women using services at the public hospitals about health during pregnancy and fetal development. Observers of antenatal care visits reported lower levels of information provision and counselling than did the clients. According to their observations, only 31 percent of women received any information and counselling at the MOH hospital, compared with 23 percent at the SSK hospital and 44 percent at the private hospital (c2 = 6.18; p = 0.045)  Safe: Proportions of providers who washed hands before or after exam: 22.9 (Ministry of Health Hospital), 0 (SSK Hospital), 5.5 (Private Hospital)  People-Centred: Women at private hospitals were able to see the same doctor each visit for better continuity of care. Women indicated that they were willing to spent extra money to receive more time and attention at a doctor’s private office, while using SSK services elsewhere. The mean duration of ANC visit, based on observational data, was 11 minutes at the Ministry of Health hospital, four minutes at the SSK hospital, and seven minutes at the private hospital. Women reported receiving significant more information at private hospitals than those at public hospitals. Observations reported lower levels of information and counselling than women themselves | Strong |
| (Tyagi, Hanson et al. 2018) [India] | To i) measure compliance for hand hygiene practices in newborn care units and labour rooms, and ii) identify the variations in hand hygiene practices by type, level and the load of the facility | 85 hospitals (public and private) | Delivery of quality care by the private sector | Observations | Effective | Effective: In only 23% of the contacts, hand-hygiene compliance was followed as per the standards with marked differences between public (12%) and private facilities (44%), p < 0.001 | Weak |
| (Victora, Matijasevich et al. 2010) [Brazil] | To document socio-economic and racial/ethnic inequalities in procedures performed during ANC | 4244 mothers | Delivery of quality care by the private sector | Cohort study | Equitable | Equitable: Found that the differences in quality of care in public and private sectors are more marked than differences due to socio-economic status or skin colour, and that variations according to income and skin colour were about attendance than discrimination | Weak |
| (Vora, Saiyed et al. 2018) [India] | To determine the quality of free delivery care and examine the difference between public and accredited private sector facilities | 1,616 women of reproductive age | Delivery of quality care by the private sector | Regression analysis | Safe, People-Centred, Effective | Effective: 34% conducted a non-evidence based episiotomy  Safe: More than half of the deliveries were assisted by nurse/auxiliary nurse midwife lady health visitor (57%) while gynaecologists conducted 22% deliveries. General doctors and unqualified personnel delivered 10.5% of mothers each.  People-Centred: Quality of care indicators, such as mothers being allowed to eat or change positions during labour, were significantly better in private than free delivery care. | Weak |
| (Waiswa, Akuze et al. 2015) [Uganda] | To explore the determinants of the use of quality of public and private maternity care | Public, private for-profit, and not-for-profit providers of childbirth and newborn care | As part of the Uganda Newborn Study (UNEST), they engaged public as well as private for-profit and not-for-profit providers through sensitisation, training, and supervision around childbirth and new- born care. UNEST aimed at improving newborn survival through a community-based intervention using home visits by volunteers linked to health facilities. The intervention included a health system strengthening component and improving linkages between the community-based intervention and the health facilities. | Pre- and post-intervention | People-Centred, Effective | Effective: Babies born in public health facilities were more likely to receive more individual newborn care practices compared to their private health facility counterparts. Whereas 42.8% of babies born in public facilities received at least eight essential newborn care practices, only 27.5% in private facilities received the same number. Nearly all (98%) babies born in public health facilities received at least three practices, compared to 95% amongst those in private health facilities  People-Centred: With the exception of immediate breastfeeding, the coverage of individual essential newborn care practices was higher but not significantly different in public sector facilities compared to private facilities | Moderate |
| (Wendot, Scott et al. 2018) [Kenya] | To assess whether post abortion family planning (PAFP) and long-acting reversible contraceptive uptake increased after a quality management intervention in private clinics | 12 clinics providing safe abortion or post abortion care in Western Kenya | A quality management intervention aimed to increase the uptake of highly effective methods of contraception following abortions. Providers attended a one-day orientation covering: a discussion on PAFP, counselling PAFP, training on a job aide given to service providers, values clarification, re-orientation on data reporting for PAFP. A one-page guide to PAFP was provided and a checklist for safe abortion provision. Franchised service providers received monthly supervision visits. | Pre-and-post intervention analysis | People-Centred, Safe | Safe: Several providers noted that they had seen a change in the structure of their supervision visits and now regularly discussed quality issues including FP provision with their supervisor. Providers characterised the visits as providing encouragement and quality assurance  People-Centred: Sixty-one percent of women reported that the provider counselled them on ways to prevent pregnancy at post-intervention, compared to 55% at baseline. A greater proportion of women reported that  the provider asked them which FP methods they had  used before and whether they had experienced problems with these methods at post-intervention. The mean number of methods women were counselled on increased from 2.9 to 3.3 | Weak |
| (Zaidi, Riaz et al. 2015) [Pakistan] | An assessment of contracted facilities and government managed facilitiN/es | Two contracted-out rural health centres and four matching government-managed rural health centres | Contracting out of MNCH services since 2008 to national NGOs, including provision of facility-based routine and basic emergency obstetric and newborn care (BEmONC) services | Controlled clinical trial | Effective, Safe, People-Centred | Effective: Rural health centres (RHCs) scored higher in terms of functionality as compared to control RHCs, having supplies and equipment in accordance with MNCH program guidelines for RHC, presence of waste disposal mechanism, and availability of Health Management Information System records. Contracted RHCs did not fare better than control RHCs with respect to staff capacity judged in terms of training and knowledge scores. Similarly, there was also no overall difference across contracted and control RHCs in the technical process of service provision.  Safe: Contracted RHCs performed better than control RHCs in three out of five BSC domains and had a higher composite score. They also had better staff satisfaction levels regarding satisfaction with facility work environment and supervisory visits. Contracted RHCs also scored higher in terms of functionality as compared to control RHCs, having supplies and equipment in accordance with MNCH program guidelines for RHC, presence of waste disposal mechanism, and availability of Health Management Information System records.  People-Centred: antenatal assessment and communication to mothers on danger signs and newborn care were comparably similar between contracted RHCs and control RHCs | Moderate |

* As noted in our manuscript, we used two quality assessment tools for quantitative (EPHPP 2010) and qualitative research (Walsh and Downe 2006, Solnes Miltenburg, Roggeveen et al. 2013). Both tools were applied to mixed-methods studies. Certain studies, reports, and abstracts lacked the information required to use these tools. For example, some reports lacked a methodology section required for us to make a comprehensive quality. Due to impact of the missing information on the quality assessment process, these particular studies have been categorised in the table above as “weak.” However, the original study or subsequent publications may provide additional information that warrants a different rating for the study.

Abt Associates (2015). Malawi program profile. Strengthening Health Outcomes through the Private Sector, Bethesda, Maryland, SHOPS, 2015 Aug.**:** [32] p.

Abuya, T. O., C. S. Molynuex, A. S. Orago, S. Were and V. Marsh (2004). "Quality of care provided to febrile children presenting in rural private clinics on the Kenyan coast." African health sciences **4**(3): 160-170.

Agha, S. (2009). The impact of a quality improvement package on the quality of reproductive health services delivered by private providers in Uganda, Bethesda, Maryland, Abt Associates, Private Sector Partnerships-One [PSP-One], 2009 Jul.**:** [38] p.

Agha, S., A. Balal and F. Ogojo-Okello (2004). "The impact of a microfinance program on client perceptions of the quality of care provided by private sector midwives in Uganda." Health Services Research **39**(6p2): 2081-2100.

Agha, S., A. Karim, A. Balal and S. Sosler (2003). A quasi-experimental study to assess the performance of a reproductive health franchise in Nepal. Country Research Series. Washington, D.C., USAID/Commercial Market Strategies Project**:** 38 p.

Akhter, S. and S. Schech (2018). "Choosing caesareans? The perceptions and experiences of childbirth among mothers from higher socio-economic households in Dhaka." Health Care for Women International **39**(11): 1177-1192.

Akwara, P., S. Alayon, S. Barry, C. Lettenmaier and V. David (2003). Uganda Delivery of Improved Services for Health (DISH) facility survey 2002, Chapel Hill, North Carolina, University of North Carolina at Chapel Hill, Carolina Population Center, MEASURE Evaluation Project, 2003 May.**:** [125] p.

Allam, R. R., G. Oruganti, C. Uthappa, N. Simhachalam, J. Rajesh and V. Yeldandi (2016). "APAIDSON program evaluation of the largest private public partnership consortium for HIV/AIDS care and treatment in India." International Journal of Infectious Diseases **45**(SUPPL. 1): 215.

Anand, S. and R. K. Sinha (2010). "Quality differentials and reproductive health service utilisation determinants in India." International Journal of Health Care Quality Assurance **23**(8): 718-729.

Angeles, G., P. Hutchinson and M. S. Khan (2003). 2001 Rural Service Delivery Partnership Evaluation Survey. Household survey report, Chapel Hill, North Carolina, University of North Carolina at Chapel Hill, Carolina Population Center [CPC], MEASURE Evaluation, 2003 Feb.**:** [182] p.

Angeles, G., P. Lance, P. Hutchinson, S. N. Mitra and S. Islam (2005). 2003 Urban NGO Service Delivery Program (NSDP) evaluation survey, [Chapel Hill, North Carolina], University of North Carolina at Chapel Hill, Carolina Population Center [CPC], MEASURE Evaluation, 2005 Mar.**:** [193] p.

Angeles, G., P. Lance and M. S. Khan (2006). 2005 Rural NGO Service Delivery Program (NSDP) evaluation survey, Chapel Hill, North Carolina, University of North Carolina at Chapel Hill, Carolina Population Center [CPC], MEASURE Evaluation, 2006 Aug.**:** [186] p.

Annigeri, V. B., L. Prosser, J. Reynolds and R. Roy (2004). An assessment of public-private partnership opportunities in India, Washington, D.C., LTG Associates, Population Technical Assistance Project [POPTECH], 2004 Nov.**:** [71] p.

Anwar, I., T. Begum, A. Rahman, H. Nababan and R. Islam (2016). "Quality of Maternal and Neonatal Health (MNH) care in for profit private sectors in urban Bangladesh." European Journal of Public Health **26**: 264-264.

Arrieta, A., A. García-Prado and J. Guillén (2011). "The Private Health Care Sector and the Provision of Prenatal Care Services in Latin America." World Development **39**(4): 579-587.

Baig, K. and F. Shahid (2017). "Training healthcare workers to improve the quality of maternal, newborn & child healthcare services in marginalized settings." International Journal for Quality in Health Care **29**(Supplement 1): 41-42.

Bakibinga, P., A. K. Ziraba, R. Ettarh, E. Kamande, T. Egondi and C. Kyobutungi (2016). "Use of private and public health facilities for essential maternal and child health services in Nairobi City informal settlements: Perspectives of women and community health volunteers." African Population Studies **30**(3): 3113-3123.

Baliga, B. S., S. R. Ravikiran, S. S. Rao, A. Coutinho and A. Jain (2016). "Public-Private Partnership in Health Care: A Comparative Cross-sectional Study of Perceived Quality of Care Among Parents of Children Admitted in Two Government District-hospitals, Southern India." J Clin Diagn Res **10**(2): Sc05-09.

Banerjee, S. K., K. L. Andersen, D. Navin and G. Mathias (2015). "Expanding availability of safe abortion services through private sector accreditation: a case study of the Yukti Yojana program in Bihar, India." Reproductive Health **12**: 1-11.

Bangladesh. National Institute of Population, R., Training, H. Bangladesh. Ministry of, W. Family, C. Associates for, R. Population and I. C. F. I. D. Program (2016). Bangladesh Health Facility Survey 2014. Final report, Dhaka, Bangladesh, NIPORT, 2015 Apr.**:** 276 p.

Barber, S. L. (2006). "Public and private prenatal care providers in urban Mexico: how does their quality compare?" International Journal for Quality in Health Care **18**(4): 306-313.

Barber, S. L., S. M. Bertozzi and P. J. Gertler (2007). "Variations in prenatal care quality for the rural poor in Mexico." Health Affairs **26**(3): w310-323.

Barber, S. L., P. J. Gertler and P. Harimurti (2007). "Differences in access to high-quality outpatient care in Indonesia." Health Affairs **26**(3): w352-366.

Bell, S. O., L. Zimmerman, Y. Choi and M. J. Hindin (2018). "Legal but limited? Abortion service availability and readiness assessment in Nepal." Health Policy and Planning **33**(1): 99-106.

Benova, L., M. L. Dennis, I. L. Lange, O. M. R. Campbell, P. Waiswa, M. Haemmerli, Y. Fernandez, K. Kerber, J. E. Lawn, A. C. Santos, F. Matovu, D. Macleod, C. Goodman, L. Penn-Kekana, F. Ssengooba and C. A. Lynch (2018). "Two decades of antenatal and delivery care in Uganda: a cross-sectional study using Demographic and Health Surveys." BMC Health Services Research **18**(1): N.PAG-N.PAG.

Benova, L., D. Macleod, K. Footman, F. Cavallaro, C. A. Lynch and O. M. R. Campbell (2015). "Role of the private sector in childbirth care: cross-sectional survey evidence from 57 low- and middle-income countries using Demographic and Health Surveys." Tropical Medicine & International Health **20**(12): 1657-1673.

Bjorkman-Nykvist, M., A. Guariso, J. Svensson and D. Yanagizawa-Drott. (2015). "Abstract: Evaluating the impact of the Living Goods entrepreneurial model of community health delivery in Uganda: A cluster-randomized controlled trial." Retrieved 4 Feb 2020, from <https://healthmarketinnovations.org/sites/default/les/Abstract_CHP2014.pdf>.

Bojalil, R., H. Guiscafré, P. Espinosa, H. Martínez, M. Palafox, G. Romero and G. Gutiérrez (1998). "The quality of private and public primary health care management of children with diarrhoea and acute respiratory infections in Tlaxcala, Mexico." Health policy and planning **13**(3): 323-331.

Bojalil, R., B. R. Kirkwood, M. Bobak and H. Guiscafre (2007). "The relative contribution of case management and inadequate care-seeking behaviour to childhood deaths from diarrhoea and acute respiratory infections in Hidalgo, Mexico." Tropical Medicine and International Health **12**(12): 1545-1552.

Boller, C., K. Wyss, M. Deo and M. Tanner (2003). "Quality and comparison of antenatal care in public and private providers in the Unitd Republic of Tanzania." World Health Organization. Bulletin of the World Health Organization **81**(2): 116-122.

Carter, E., M. Ndhlovu, E. Nkhama, M. Munos, J. Katz and T. P. Eisele (2016). "Linking household and point-of-care data to estimate coverage of appropriate management of childhood illness in Southern Province, Zambia." American Journal of Tropical Medicine and Hygiene **95**(5 Supplement 1): 262-263.

Chakraborty, S., S. A. D'Souza and R. S. Northrup (2000). "Improving private practitioner care of sick children: testing new approaches in rural Bihar." Health Policy & Planning **15**(4): 400-407.

Chakraborty, S. and K. Frick (2002). "Factors influencing private health providers' technical quality of care for acute respiratory infections among under-five children in rural West Bengal, India." Social Science & Medicine **55**(9): 1579-1587.

Chemonics, I. (2015). Private Sector Mobilization for Family Health -- Phase 2 (PRISM2) Project. Final report, [Manila], Philippines, Chemonics International, 2015 Jan.**:** 52 p.

Chen, L. I., Y. Dai, Y. Zhang, Q. Wu, D. Rudan, V. Saftic, M. H. M. M. T. van Velthoven, J. Su, Z. Tan and R. W. Scherpbier (2013). "A comparison between antenatal care quality in public and private sector in Rural Hebei, China." Croatian Medical Journal **54**(2): 146-156.

Coulibaly, F., H. DeLisle and S. Haddad (2002). "Mothers perception of quality of growth monitoring and promotion programs: A qualitative study in Cote d'Ivoire." Ecology of Food and Nutrition **41**(6): 475-500.

Danel, I. and G. Forgia (2005). "Contracting for basic health care in rural Guatemala - Comparison of the performance of three delivery models." Health Systems Innovations in Central America: Lessons and Impact of New Approaches: 49-88.

de Azevedo Bittencourt, S. D., R. Queiroz Gurgel, M. A. da Silva Menezes, L. S. Bastos and M. do Carmo Leal (2015). "Neonatal care in Brazil: hospital structure and adequacy according to newborn obstetric risk." Paediatr Int Child Health **35**(3): 206-212.

Dennis, M. L., L. Benova, T. Abuya, M. Quartagno, B. Bellows and O. M. R. Campbell (2019). "Initiation and continuity of maternal healthcare: examining the role of vouchers and user-fee removal on maternal health service use in Kenya." Health Policy & Planning **34**(2): 120-131.

Diamond-Smith, N., M. Sudhinaraset and D. Montagu (2016). "Clinical and perceived quality of care for maternal, neonatal and antenatal care in Kenya and Namibia: the service provision assessment." Reproductive Health **13**: 1-13.

Do, M. (2009). Can private sector providers accurately assess the quality of services they provide? Evidence from private midwives in Uganda, Bethesda, Maryland, Abt Associates, Private Sector Partnerships-One [PSP-One], 2009 Sep.**:** [28] p.

Do, M. and S. Agha (2009). Differences in the quality of reproductive health services provided by private midwives in Uganda. MD, Private Sector Partnerships-One project, Abt Associates Inc.**:** 24 p.

Eichler, R., P. Auxila, U. Antoine and B. Desmangles (2009). Haiti: going to scale with a performance incentive model. Performance incentives for global health: potential and pitfalls. R. Eichler, R. Levine and the Performance-Based Incentives Working Group. Washington, DC, USA, Center for Global Development**:** 165-188.

Epiu, I., A. Wabule, A. Kambugu, H. Mayanja-Kizza, J. V. B. Tindimwebwa and G. Dubowitz (2017). "Key bottlenecks to the provision of safe obstetric anaesthesia in low- income countries; a cross-sectional survey of 64 hospitals in Uganda." BMC Pregnancy Childbirth **17**(1): 387.

Farahbakhsh, M., H. Sadeghi-Bazargani, A. Nikniaz, J. S. Tabrizi, A. Zakeri and S. Azami (2012). "Iran's Experience of Health Cooperatives as a Public-Private Partnership Model in Primary Health Care: A Comparative Study in East Azerbaijan." Health Promot Perspect **2**(2): 287-298.

Field, E., D. Abo, L. Samiak, M. Vila, G. Dove, A. Rosewell and S. Nathan (2018). "A Partnership Model for Improving Service Delivery in Remote Papua New Guinea: A Mixed Methods Evaluation." Int J Health Policy Manag **7**(10): 923-933.

Fischer, E., S. N. Musau and M. Corbett (2004). Kenya postabortion care sustainability study: a focus on private nurse-midwives, Chapel Hill, North Carolina, IntraHealth International, PRIME, 2004.**:** 74 p.

Gill, Z. and M. Carlough (2008). "Do mission hospitals have a role in achieving Millennium Development Goal 5?" International Journal of Gynecology & Obstetrics **102**(2): 198-202.

Haemmerli, M., A. Santos, L. Penn-Kekana, I. Lange, F. Matovu, L. Benova, K. L. M. Wong and C. Goodman (2018). "How equitable is social franchising? Case studies of three maternal healthcare franchises in Uganda and India." Health Policy & Planning **33**(3): 411-419.

Hansen, P. M., D. H. Peters, A. Edward, S. Gupta, A. Arur, H. Niayesh, G. Burnham, P. M. Hansen, D. H. Peters, A. Edward, S. Gupta, A. Arur, H. Niayesh and G. Burnham (2008). "Determinants of primary care service quality in Afghanistan." International Journal for Quality in Health Care **20**(6): 375-383.

Health Partners International and Montrose (2014). NU Health. Clinical audit of maternal and child health care services in the context of results based financing in Northern Uganda. Kampala (Uganda), NU Health Programme**:** 44 p.

Health Partners International and Montrose (2015). NU Health. Results based financing with non-state providers: insights from a controlled trail in Northern Uganda. Technical Annexes. Kampala (Uganda), NU Health Programme**:** 32 p.

Health Partners International and Montrose (2015). NU Health. Results based financing with non-state providers: insights from a controlled trial in Northern Uganda. Kampala (Uganda), NU Health Programme**:** 22 p.

Hirose, A., I. O. Yisa, A. Aminu, N. Afolabi, M. Olasunmbo, G. Oluka, K. Muhammad and J. Hussein (2018). "Technical quality of delivery care in private- and public-sector health facilities in Enugu and Lagos States, Nigeria." Health Policy & Planning **33**(5): 666-674.

Huda, F. A., A. Ahmed, E. R. Ford and H. B. Johnston (2015). "Strengthening health systems capacity to monitor and evaluate programmes targeted at reducing abortion-related maternal mortality in Jessore district, Bangladesh." BMC health services research **15**: 426.

Hulton, L. A., Z. Matthews and R. W. Stones (2007). "Applying a framework for assessing the quality of maternal health services in urban India." Social Science & Medicine **64**(10): 2083-2095.

J-PAL Policy Briefcase (2019). in the business of saving lives. Cambridge, MA, USA, Abdul Latif Jameel Poverty Action Lab.

Jayanna, K., P. Mony, B. M. Ramesh, A. Thomas, A. Gaikwad, H. L. Mohan, J. F. Blanchard, S. Moses and L. Avery (2014). "Assessment of facility readiness and provider preparedness for dealing with postpartum haemorrhage and pre-eclampsia/eclampsia in public and private health facilities of northern Karnataka, India: a cross-sectional study." Bmc Pregnancy and Childbirth **14**.

Johnson, D. and X. Cheng (2014). "The role of private health providers in HIV testing: analysis of data from 18 countries." Int J Equity Health **13**: 36.

Karki, C., M. Ojha and R. T. Rayamajhi (2009). "Baseline survey on functioning of abortion services in government approved CAC centers in three pilot districts of Nepal." Kathmandu Univ Med J (KUMJ) **7**(25): 31-39.

Khan, M. A., S. S. Owais, S. Ishaq, J. Walley, H. J. Khan, C. Blacklock, M. A. Khan and M. W. Azeem (2017). "Process evaluation of integrated early child development care at private clinics in poor urban Pakistan: a mixed methods study." BJGP Open **1**(3): bjgpopen17X101073.

Kohnke, E. J., U. K. Mukherjee and K. K. Sinha (2017). "Delivering Long-Term Surgical Care in Underserved Communities: The Enabling Role of International NPOs as Partners." Production and Operations Management **26**(6): 1092-1119.

Kojima, N., C. C. Bristow, N. Pollock, P. Crouse, H. Theodore, J. Bonhomme, C. F. Gaston, J. G. Devieux, J. W. Pape and J. D. Klausner (2015). "Rapid Training and Implementation of the Pollock Technique, a Safe, Effective Newborn Circumcision Procedure, in a Low-Resource Setting." Glob Pediatr Health **2**: 2333794x15589114.

Lance, P., G. Angeles and N. Kamal (2012). Bangladesh. Smiling Sun Franchise Program (BSSFP) impact evaluation report, Chapel Hill, North Carolina, University of North Carolina at Chapel Hill, Carolina Population Center, MEASURE Evaluation, 2012 Dec.**:** [121] p.

Lee, E., S. Madhavan and S. Bauhoff (2016). "Levels and variations in the quality of facility-based antenatal care in Kenya: evidence from the 2010 service provision assessment." Health Policy & Planning **31**(6): 777-784.

Levin, A., S. Munthali, V. Vodungbo, N. Rukhadze, K. Maitra, T. Ashagari and L. Brenzel (2019). "Scope and magnitude of private sector financing and provision of immunization in Benin, Malawi and Georgia." Vaccine **37**(27): 3568-3575.

Lind, A., A. Edward, P. Bonhoure, L. Mustafa, P. Hansen, G. Burnham, D. H. Peters, A. Lind, A. Edward, P. Bonhoure, L. Mustafa, P. Hansen, G. Burnham and D. H. Peters (2011). "Quality of outpatient hospital care for children under 5 years in Afghanistan." International Journal for Quality in Health Care **23**(2): 108-116.

MacFarlane, K. A., M. L. O'Neil, D. Tekdemir, A. M. Foster and M. L. O'Neil (2017). ""It was as if society didn't want a woman to get an abortion": a qualitative study in Istanbul, Turkey." Contraception **95**(2): 154-160.

Mahar, B., R. Kumar, N. Rizvi, H. A. Bahalkani, M. Haq and J. Soomro (2012). "Quantity and quality of information, education and communication during antenatal visit at private and public sector hospitals of Bahawalpur, Pakistan." Journal of Ayub Medical College, Abbottabad : JAMC **24**(3-4): 71-74.

Maru, D., S. Maru, I. Nirola, J. Gonzalez-Smith, A. Thoumi, P. Nepal, P. Chaudary, I. Basnett, K. Udayakumar and M. McClellan (2017). "Accountable Care Reforms Improve Women's And Children's Health In Nepal." Health Affairs **36**(11): 1965-1972.

Mbonye, A. K., E. Buregyeya, E. Rutebemberwa, S. E. Clarke, S. Lal, K. S. Hansen, P. Magnussen and P. Larussa (2016). "Treatment and prevention of malaria in pregnancy in the private health sector in Uganda: Implications for patient safety." Malaria Journal **15**(1): 212.

McIntosh, N., A. Grabowski, B. Jack, E. L. Nkabane-Nkholongo and T. Vian (2015). "GLOBAL. A Public-Private Partnership Improves Clinical Performance In A Hospital Network In Lesotho." Health Affairs **34**(6): 954-962.

Mendez, E. and Associates (2014). End-of-project performance evaluation of USAID / Caucasus Sustaining Family Planning and Maternal and Child Health (SUSTAIN) project in Georgia. Final report, Bethesda, Maryland, Mendez, England and Associates, 2014 Dec 12.**:** [91] p.

Ministry of Health (MOH) [Zambia], Central Statistical Office [Zambia] and ORC Macro (2006). Zambia HIV/AIDS Service Provision Assessment Survey 2005. Calverton, Maryland, USA, Ministry of Health, Central Statistical Office, and ORC Macro.

Mohanan, M., K. S. Babiarz, J. D. Goldhaber-Fiebert, G. Miller and M. Vera-Hernández (2016). "Effect Of A Large-Scale Social Franchising And Telemedicine Program On Childhood Diarrhea And Pneumonia Outcomes In India." Health Aff (Millwood) **35**(10): 1800-1809.

Mohanan, M., S. Giardili, V. Das, T. L. Rabin, S. S. Raj, J. I. Schwartz, A. Seth, J. D. Goldhaber-Fiebert, G. Miller and M. Vera-Hernandez (2017). "Evaluation of a social franchising and telemedicine programme and the care provided for childhood diarrhoea and pneumonia, Bihar, India." Bulletin of the World Health Organization **95**(5): 343-352.

Mony, P. K., J. Krishnamurthy, A. Thomas, K. Sankar, B. M. Ramesh, S. Moses, J. Blanchard and L. Avery (2013). "Availability and Distribution of Emergency Obstetric Care Services in Karnataka State, South India: Access and Equity Considerations." PLoS ONE **8**(5): e64126.

Murphy, G. A. V., D. Gathara, N. Abuya, J. Mwachiro, S. Ochola, R. Ayisi and M. English (2018). "What capacity exists to provide essential inpatient care to small and sick newborns in a high mortality urban setting? - A cross-sectional study in Nairobi City County, Kenya." PLoS ONE **13**(4): e0196585.

Murphy, G. A. V., D. Gathara, J. Mwachiro, N. Abuya, J. Aluvaala, M. English, S. Ochola, R. Ayisi, A. Wasunna, F. Were, C. Mutinda, B. Maina, C. Mutiso, D. Githanga, D. Kimutai, R. Musoke, R. Ochieng, W. Macharia, R. Nyamai and G. on behalf of the Health Services that Deliver for Newborns Expert (2018). "Effective coverage of essential inpatient care for small and sick newborns in a high mortality urban setting: a cross-sectional study in Nairobi City County, Kenya." BMC Medicine **16**(1): 72.

Nelson, D., M. Corbett, F. Githiori, R. Mason Jr, P. Muhuhu, R. Mulindi and F. Yumkella (2002). The right provider for the right place: private nurse-midwives offering primary-level post-abortion care in Kenya. PRIME II Dispatch. **4**.

Nikniyaz, A., M. Farahbakhsh, K. Ashjaei, D. Tabrizi, H. Sadeghi-Bazargani and A. Zakeri (2006). "Maternity and child health care services delivered by public health centers compared to health cooperatives: Iran's experience." Journal of Medical Sciences **6**(3): 352-358.

Pathfinder International (2016). Implementing performance-based grants for improved NGO performance in Bangladesh, Watertown, Massachusetts, Pathfinder International, 2016 Jul.**:** [8] p.

Penn-Kekana, L., T. Powell-Jackson, M. Haemmerli, I. L. Lange, G. Sharma, C. Goodman, V. Dutt, K. Singh, V. Shukla, A. Mahapatra and S. Singh (2018). "Process evaluation of a social franchising model to improve maternal health: evidence from a multi-methods study in Uttar Pradesh, India." Implementation Science **13**(1): N.PAG-N.PAG.

Powell-Jackson, T., D. Macleod, L. Benova, C. Lynch and O. M. R. Campbell (2015). "The role of the private sector in the provision of antenatal care: a study of Demographic and Health Surveys from 46 low- and middle-income countries." Tropical Medicine & International Health **20**(2): 230-239.

Powell-Jackson, T., L. Penn-Kekena, S. Tougher, M. Haemmerli, V. Dutt, I. Lange, A. Mahapatra, G. Sharma, K. Singh, S. Singh, V. Shukla, S. Pereira, K. Haldar, P. Kumar and C. Goodman (2018). SOCIAL FRANCHISING FOR MATERNAL HEALTH IN INDIA: Findings from an impact and process evaluation Maternal Healthcare Markets Evaluation Team Policy Brief. London, London School of Hygiene & Tropical Medicine.

Quimbo, S. A., J. W. Peabody, R. Shimkhada, K. Woo and O. Solon (2008). "Should we have confidence if a physician is accredited? A study of the relative impacts of accreditation and insurance payments on quality of care in the Philippines." Social Science & Medicine **67**(4): 505-510.

Rahman, M., U. Rob and T. Kibria (2009). Implementation of maternal health financial scheme in rural Bangladesh.

Ramachandar, L. and P. J. Pelto (2002). "The role of village health nurses in mediating abortions in rural Tamil Nadu, India." Reprod Health Matters **10**(19): 64-75.

Ramachandar, L. and P. J. Pelto (2004). "Abortion Providers and Safety of Abortion: A Community-Based Study in a Rural District of Tamil Nadu, India." Reproductive Health Matters **12**(24, Supplement): 138-146.

Rannan-Eliya, R. P., N. Wijemanne, I. K. Liyanage, S. Dalpatadu, S. de Alwis, S. Amarasinghe and S. Shanthikumar (2015). "Quality of inpatient care in public and private hospitals in Sri Lanka." Health Policy & Planning **30**: i46-i58.

Rannan-Eliya, R. P., N. Wijemanne, I. K. Liyanage, J. Jayanthan, S. Dalpatadu, S. Amarasinghe and C. Anuranga (2015). "The quality of outpatient primary care in public and private sectors in Sri Lanka-how well do patient perceptions match reality and what are the implications?" Health Policy and Planning **30**: i59-i74.

Ridde, V., A. Diarra, V. Ridde and A. Diarra (2009). "A process evaluation of user fees abolition for pregnant women and children under five years in two districts in Niger (West Africa)." BMC Health Services Research **9**: 89-89.

Rolfe, B., S. Leshabari, F. Rutta and S. F. Murray (2008). "The crisis in human resources for health care and the potential of a 'retired' workforce: case study of the independent midwifery sector in Tanzania." Health Policy & Planning **23**(2): 137-149.

Ross, S. R., D. Mankad, N. Hajeebhoy and J. Tomaro (2005). Final evaluation: Gujarat Health System Development Project (GHSDP), Sidhpur and Junagadh, India. Aga Khan Foundation U.S.A (Grant Agreement / Award No. FAO-A-00-98-00078-00). October 25, 2004 – December 31, 2004, Washington, D.C., Aga Khan Foundation U.S.A, [2005]. [79] p.

Sathar, Z., S. Singh, Z. Shah, G. Rashida and I. Kamran (2013). Post-abortion care in Pakistan: A national study. Islamabad, Population Council**:** 140 p.

Save the Children (2007). Umoyo Newtork Capacity Building for Quality HIV / AIDS Services Project: Final Report, Save the Children.

Schooley, J., C. Mundt, P. Wagner, J. Fullerton and M. O'Donnell (2009). "Factors influencing health care-seeking behaviours among Mayan women in Guatemala." Midwifery **25**(4): 411-421.

Sharan, M., S. Ahmed, A. Malata and K. Rogo (2012). "The quality of public and private maternal health systems in malawi [NOTE: just an abstract of conference presentation - no full PDF]." International Journal of Gynecology and Obstetrics **119**(SUPPL. 3): S479-S480.

Sharma, G., T. Powell-Jackson, K. Haldar, J. Bradley and V. Filippi (2017). "Quality of routine essential care during childbirth: clinical observations of uncomplicated births in Uttar Pradesh, India." World Health Organization. Bulletin of the World Health Organization **95**(6): 419-429.

Sharma, G., T. Powell-Jackson, K. Haldar, J. Bradley and V. Filippi (2018). Quality of care during childbirth in Uttar Pradesh, India. Maternal Healthcare Markets Evaluation Team Policy Brief. London, London School of Hygiene & Tropical Medicine.

Sieverding, M., C. Briegleb and D. Montagu (2015). "User experiences with clinical social franchising: qualitative insights from providers and clients in Ghana and Kenya." BMC Health Services Research **15**(1): 49.

Singh, V., S. Ahmed, M. L. Dreyfuss, U. Kiran, D. N. Chaudhery, V. K. Srivastava, R. C. Ahuja, A. H. Baqui, G. L. Darmstadt, M. Santosham and K. P. West, Jr. (2017). "Non-governmental organization facilitation of a community-based nutrition and health program: Effect on program exposure and associated infant feeding practices in rural India." PLoS One **12**(9): e0183316.

Tilford, K. and S. Amjad (2016). Final evaluation of the Saving Mothers and Newborns in Communities Project. A focused strategic assessment of a community midwives program in three districts of Balochistan province, Pakistan. CSHGP Cooperative Agreement Number: AID-OAA-A-12-00093. Testing interventions to strengthen a private-sector community midwives program to improve maternal and newborn health status in underserved areas of Balochistan province, Pakistan, [Washington, D.C.], United States Agency for International Development [USAID], 2016 Nov.**:** 388 p.

Turan, J. M., A. Bulut, H. Nalbant, N. Ortayli and A. A. Akalin (2006). "The quality of hospital-based antenatal care in Istanbul." Stud Fam Plann **37**(1): 49-60.

Tyagi, M., C. Hanson, J. Schellenberg, S. Chamarty and S. Singh (2018). "Hand hygiene in hospitals: an observational study in hospitals from two southern states of India." BMC Public Health **18**(1): N.PAG-N.PAG.

Victora, C. G., A. Matijasevich, M. Silveira, I. Santos, A. J. Barros and F. C. Barros (2010). "Socio-economic and ethnic group inequities in antenatal care quality in the public and private sector in Brazil." Health Policy & Planning **25**(4): 253-261.

Vora, K. S., S. L. Saiyed and D. V. Mavalankar (2018). "Quality of Free Delivery Care among Poor Mothers in Gujarat, India: A Community-Based Study." Indian Journal of Community Medicine **43**(3): 224-228.

Waiswa, P., J. Akuze, S. Peterson, K. Kerber, M. Tetui, B. C. Forsberg and C. Hanson (2015). "Differences in essential newborn care at birth between private and public health facilities in eastern Uganda." Global Health Action **8**: 1-9.

Wendot, S., R. H. Scott, I. Nafula, I. Theuri, E. Ikiugu and K. Footman (2018). "Evaluating the impact of a quality management intervention on post-abortion contraceptive uptake in private sector clinics in western Kenya: a pre- and post-intervention study." Reprod Health **15**(1): 10.

Zaidi, S., A. Riaz, F. Rabbani, S. I. Azam, S. N. Imran, N. A. Pradhan and G. N. Khan (2015). "Can contracted out health facilities improve access, equity, and quality of maternal and newborn health services? Evidence from Pakistan." Health Research Policy & Systems **13**(1): 54-54.
