# Supplementary figures and images for: Private sector quality of care for maternal, new-born, and child health in low-and-middle-income countries: a secondary review
Source: Front Glob Womens Health. 2024 Apr 19;5:1369792. doi: 10.3389/fgwh.2024.1369792 (PMC11066217; doi:10.3389/fgwh.2024.1369792)

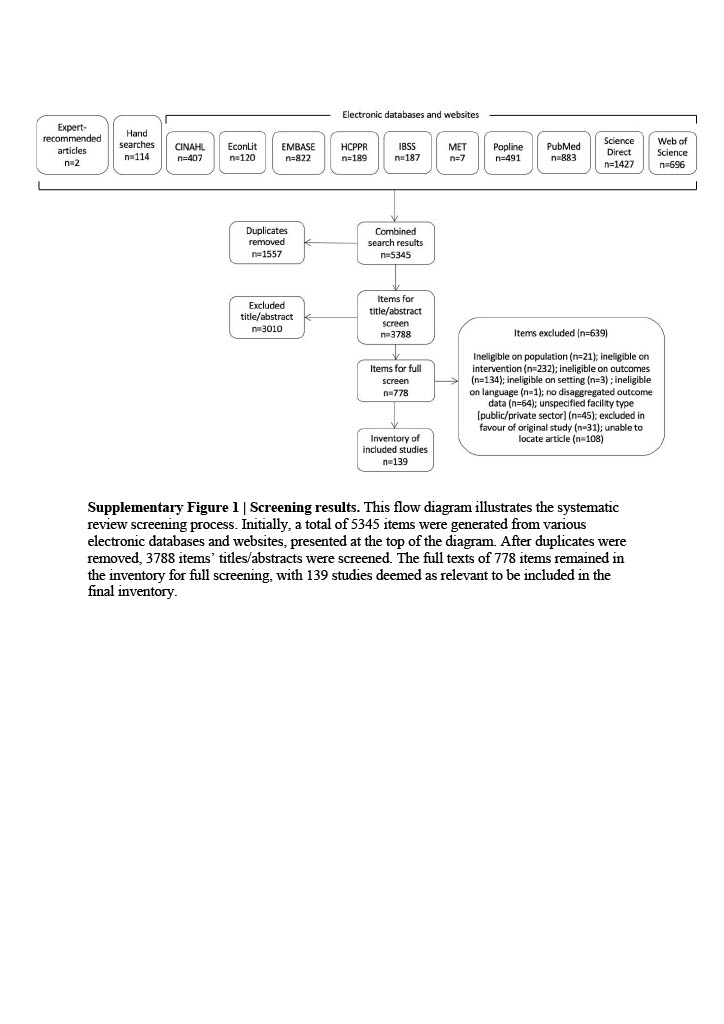

Supplement: Supplementary file 2 [file Image1.jpg]
